# Co-exposure to urban particulate matter and aircraft noise adversely impacts the cerebro-pulmonary-cardiovascular axis in mice

**DOI:** 10.1016/j.redox.2022.102580

**Published:** 2022-12-18

**Authors:** Marin Kuntic, Ivana Kuntic, Roopesh Krishnankutty, Adrian Gericke, Matthias Oelze, Tristan Junglas, Maria Teresa Bayo Jimenez, Paul Stamm, Margaret Nandudu, Omar Hahad, Karin Keppeler, Steffen Daub, Ksenija Vujacic-Mirski, Sanela Rajlic, Lea Strohm, Henning Ubbens, Qi Tang, Subao Jiang, Yue Ruan, Kenneth G. Macleod, Sebastian Steven, Thomas Berkemeier, Ulrich Pöschl, Jos Lelieveld, Hartmut Kleinert, Alex von Kriegsheim, Andreas Daiber, Thomas Münzel

**Affiliations:** a University Medical Center Mainz, Department for Cardiology 1, Molecular Cardiology, Langenbeckstr. 1, 55131, Mainz, Germany; b Institute of Genetics and Cancer, University of Edinburgh, UK; c Department of Ophthalmology, University Medical Center of the Johannes Gutenberg-University Mainz, Langenbeckstr. 1, 55131, Mainz, Germany; d German Center for Cardiovascular Research (DZHK), Partner Site Rhine-Main, Mainz, Germany; e Department of Cardiothoracic and Vascular Surgery, University Medical Center of the Johannes Gutenberg-University Mainz, Langenbeckstr. 1, 55131, Mainz, Germany; f Max Planck Institute for Chemistry, Multiphase Chemistry Department, Mainz, Germany; g Max Planck Institute for Chemistry, Atmospheric Chemistry Department, Mainz, Germany; h University Medical Center Mainz, Department for Pharmacology, Langenbeckstr. 1, 55131, Mainz, Germany

**Keywords:** Environmental risk factors, Traffic noise exposure, Air pollution, Oxidative stress, Inflammation, Cardiovascular risk

## Abstract

Worldwide, up to 8.8 million excess deaths/year have been attributed to air pollution, mainly due to the exposure to fine particulate matter (PM). Traffic-related noise is an additional contributor to global mortality and morbidity. Both health risk factors substantially contribute to cardiovascular, metabolic and neuropsychiatric sequelae. Studies on the combined exposure are rare and urgently needed because of frequent co-occurrence of both risk factors in urban and industrial settings. To study the synergistic effects of PM and noise, we used an exposure system equipped with aerosol generator and loud-speakers, where C57BL/6 mice were acutely exposed for 3d to either ambient PM (NIST particles) and/or noise (aircraft landing and take-off events). The combination of both stressors caused endothelial dysfunction, increased blood pressure, oxidative stress and inflammation. An additive impairment of endothelial function was observed in isolated aortic rings and even more pronounced in cerebral and retinal arterioles. The increase in oxidative stress and inflammation markers together with RNA sequencing data indicate that noise particularly affects the brain and PM the lungs. The combination of both stressors has additive adverse effects on the cardiovascular system that are based on PM-induced systemic inflammation and noise-triggered stress hormone signaling. We demonstrate an additive upregulation of ACE-2 in the lung, suggesting that there may be an increased vulnerability to COVID-19 infection. The data warrant further mechanistic studies to characterize the propagation of primary target tissue damage (lung, brain) to remote organs such as aorta and heart by combined noise and PM exposure.

## Highlights

• Traffic noise and particulate matter (PM) always co-localize in urban environments.
• New prototype exposure system for combined exposure of mice to traffic noise and PM.
• First mechanistic insights in multi-organ damage by combined exposure to noise and PM.
• Characterization of brain damage by noise, lung damage by PM and vascular/systemic damage by combination.
• Evaluation of multi-organ gene expression by RNA sequencing and cytokine profiles by array technique.

## 1 Introduction

The Lancet Commission on Pollution and Health reports that air pollution is currently the largest global environmental cause of disease and excess mortality. Diseases caused by pollution caused an estimated 9 million premature deaths/year worldwide [1]. The WHO also estimates that up to 12.6 million global deaths/year are due to living in unhealthy environments [2,3]. The Global Burden of Disease (GBD) Study 2019 updated these estimates, indicating that environmental factors (with air pollution ranking as the fourth most important global risk factor) facilitate the development of chronic non-communicable diseases (NCD) and significantly contribute to global mortality [4,5]. Data from the GBD study indicate that pollution was responsible for 268 million disability-adjusted life years (DALYs) [6].

Ambient air pollution, in the form of fine particulate matter (i.e. particles with a diameter <2.5 μm [PM2.5]) and reactive gases (such as O_3_ and ^•^NO_2_) markedly contribute to cardiovascular and cerebrovascular disease and excess mortality [[7], [8], [9]]. The association of PM2.5 with cardiovascular morbidity/mortality is substantial (see guidelines of EU/US cardiovascular societies [10,11]). For example, the ESCAPE project established a significant (13%) increase in non-fatal acute coronary events per 5 μg/m^3^ elevation in long-term exposure to PM2.5 [12]. In 2020, an excess mortality rate of about 8.8 million deaths/year was attributed to air pollution (mostly of cardiovascular origin), which reflects 15% of the annual global deaths from all causes [13,14].

In addition to the well-established health risks of air pollution [15], environmental noise is another transport-related health risk factor [[16], [17], [18], [19]]. Despite growing evidence that links traffic noise to cardiovascular morbidity/mortality, ambient noise is neither mentioned in the GBD Study [20] nor in the report “Health at a Glance: Europe 2018” [21]. The WHO environmental noise guidelines for the EU region stated that traffic noise can affect cardiometabolic disease but strongly advocated additional high-quality longitudinal studies [22]. The pooled relative risk for ischemic heart disease of 1.08 per 10 dB(A) increase in noise exposure is striking [22]. Traffic noise is also a potential risk factor for mental disease and cognitive impairment, especially in children [23], as well as for depression, anxiety disorders [24] and dementia particularly related to Alzheimer’s disease [25]. The mechanistic explanation for these epidemiological adverse health effects of noise may be the “cerebral” link between noise stimulus, vascular inflammation and adverse cardiovascular events, which are obviously based on amygdalar activation upon exposure to transportation noise [26].

According to the WHO, at least 1.6 million healthy life years are lost annually from traffic noise in Western Europe [27]. Studies on air pollution and noise exposure indicate that pathophysiological pathways causing health side effects are similar, as indicated by additive cardiovascular damage and risk [28,29]. It is well established that environmental stressors are co-located, e.g., in urban and industrial areas, and accordingly combined exposure studies are highly warranted [30]. In addition, air and noise pollution have many of the same sources, such as heavy industry, aircraft, railways and road vehicles. Research suggests that the social cost of noise and air pollution in the EU — including excess death and disease — could be more than 1 trillion EUR [31], e.g. greatly exceeding social cost of alcohol use (∼ 125 billion EUR) [32] and smoking (544 billion EUR) [33]. This is supported by the statement “The control of ambient air pollution in the United States has yielded about $30 in benefits for every $1 invested” [1].

Despite the urgent need for multi-exposure models, most studies on combined effects of noise and air pollution thus far rely on mathematical rather than experimental models, warranting studies under controlled laboratory conditions. Only a few studies have addressed the mutual effects of traffic noise and air pollution due to their interconnected nature [29,[34], [35], [36], [37]]. Here, we report the first study on the adverse health effects of single versus combined exposure of mice to aircraft noise and airborne particulate matter with special emphasis on the damage affecting the cerebro-pulmonary-cardiovascular axis. To address this issue, we employed a unique, custom-built exposure system equipped with an aerosol generator (collision nebulizer) for exposure of urban PM2.5 suspensions from the National Institute of Standards and Technology (NIST) and loud-speakers to produce transportation noise (aircraft noise).

Specifically, we addressed the following questions:

1) What is the ideal suspension of PM (NIST) to study the effects of air pollution? Ideally, it should contain a high fraction of PM with a diameter of 2.5 μm and lead to oxidative stress, inflammation and endothelial dysfunction, representing the main adverse effects of PM [8].
2) What are the health effects of individual exposures to noise and air pollution?
3) Are there additional health effects from the co-exposure to noise and air pollution related to the cardiovascular and cerebral system and the lungs?
4) What is the impact of co-exposure to both environmental stressors on the expression of genes in various affected organs?

## 2 Methods

### 2.1 Particulate matter and noise exposure system

The system for the combined PM and noise exposure is illustrated in suppl. Fig. S1. The system was acquired from TSE Systems GmbH (Hochtaunuskreis, Germany), and consists of a PM source (collision nebulizer), a large drying column, an exposure chamber, a PM monitoring device, numerous filters and mass flow controllers. The exposure chamber is equipped with two loudspeakers for noise exposure. The system is operated through two separate proprietary software programs installed on a Windows PC, where one operates the PM monitoring device and the other operates the airflow through the system. The most important parts of the system are described in full detail in the Extended Methods in the online supplement. We used PM from the National Institute of Standards and Technology (NIST; Gaithersburg, MD, USA).

### 2.2 Particulate matter and noise exposure protocol

**Fig. 1:**
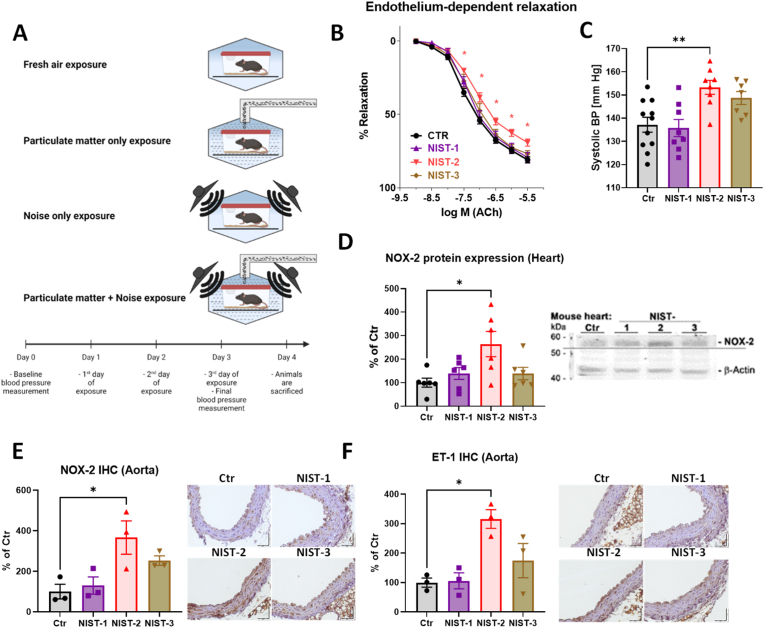
*Mouse exposure protocol and testing of different preparations of fine particulate matter (NIST PM).* Timeline and schematic illustration of the exposure protocol for both PM and noise (A). The vascular function was measured in isolated aortic rings (B). The endothelium-dependent relaxation in presence of acetylcholine (ACh) was significantly impaired for the NIST2 PM preparation, but not for the other NIST preparations. The endothelium-independent relaxation in presence of nitro-glycerine (GTN) was not changed after the exposure to any of the NIST PM preparations. The blood pressure of the exposed mice was measured by the non-invasive tail cuff method (C). Systolic blood pressure was significantly increased for the NIST2 PM preparation, but not for other preparations, although NIST3 showed an increase by trend. The Western blot (WB) of the heart tissue proteins, showed a significant increase of NOX-2 in the NIST2 group, but not in the other NIST PM preparation groups (D). Representative original blots are shown besides the quantification. Immunohistochemical staining revealed that NOX-2 and endothelin-1 protein was also significantly upregulated by PM (NIST2) exposure, whereas only a minor trend of increase was seen with NIST3 and no effect was observed with NIST1. Representative immunohistochemical staining images are shown besides the quantification. Data are presented as mean ± SEM and individual values are shown where possible. The statistics was performed from n = 11–19 (B), 7–11 (C), 6 (D), 3 (E,F) independent experiments. The statistical significance of p < 0.05 is represented with an asterisk (*).

All animals were treated in accordance with the Guide for the Care and Use of Laboratory Animals as adopted by the U.S. National Institutes of Health and approval was granted by the Ethics Committee of the University Medical Center Mainz and the Landesuntersuchungsamt Rheinland-Pfalz (Koblenz, Germany; permit number: 23 177-07/G 16-1-055 and 20-1-055). For the main study, a total of 172 male C57BL/6J mice were exposed to either PM only (n = 47), noise only (n = 40), combination of PM and noise (n = 42) or to fresh air and no noise (n = 43) (see treatment protocol and equipment in Fig. 1A). For the pilot study, a total of 34 male C57BL/6J mice were exposed to either clean air (11), NIST1 (8), NIST2 (8) or NIST3 (7). While mice were in the exposure chamber, being exposed to PM only or PM and noise, they had access to water, but not to food, as ingestion of PM was not desired. For the exposure to NIST particles only, mice were exposed for 6 h per day for 3 days. The target concentration of NIST particles in the chamber was 200 μg/m^3^, as explained in the supplement, which is justified by the short exposure protocol and real world particulate matter concentrations reaching up to 200 μg/m^3^ in highly polluted cities [38]. During the noise only exposure, mice were exposed to the same conditions as the mice exposed to PM only. The aircraft noise was a 2 h long noise pattern of 69 aircraft noise events with a duration of 43 s each and a maximum sound pressure level of 85 dB(A) and an average sound pressure level of 72 dB(A), that was recorded at the Düsseldorf airport. Mice were exposed to this noise recording on a loop for 9 ± 3 h per day for 3 days. For simultaneous exposure of mice to PM and noise, a combined protocol was used for 6 h per day, for 3 days. All exposures occurred during the daytime – mouse sleeping phase. In a pilot study, we compared 3 different NIST PM mixtures, NIST1 (SRM1650b), NIST2 (SRM1648a) and NIST3 (SRM2786), in order to identify the particles with the most reproducible detrimental adverse health effects. The detailed description of these particles is provided in the Extended Methods in the online supplement and their particle size distribution is shown in suppl. Fig. S2.

NIST2 particles were identified as the most detrimental urban particulate matter mixture in our head-to-head comparisons. These were urban particles collected in St. Louis, Missouri during 12 months in 1976 and 1977. These particles contain toxic and redox-active metalloids as well as metals (e.g. Sb, As, Cd, Ce, Co, Cr, Co, Fe, Pb, Mn, Hg, Ni, Rb, Sr, Ti, V, Cs, La), a number of highly toxic (nitro) polycyclic aromatic hydrocarbons (PAHs) such as pyrenes and anthracenes as well as several polychlorinated biphenyls (PCBs) and chlorinated pesticides (see SRM1648a datasheet in the supplement). The content of biogenic (e.g. endotoxins) and anthropogenic contaminations (e.g. metals, PAHs, PCBs) on the particles, partially taken-up in urban space or generated by photochemical reactions during particle atmospheric aging, seems to represent a major determinant of their biological toxicity [39]. The metal load of particles is obviously a major contributor to cardiopulmonary damage in animals [40]. The size distribution of particles is from 0.3 to 100 μm with 10% of particle volume below a particle diameter of 1.35 μm, 50% of particle volume below a particle diameter of 5.85 μm and 90% of particle volume below a particle diameter of 30.1 μm. More detailed information on mass concentration of particles of different sizes in the exposure chamber are provided in the online supplement.

### 2.3 Other parameters

All other methodological details, e.g., for blood pressure measurements by tail cuff, vasoreactivity studies by isometric tension technique, immunohistochemistry, Western blotting, RT-PCR, envisaging of reactive oxygen species (ROS) formation by fluorescent dihydroethidium microtopography, cytokine array, RNA sequencing and bioinformatical analysis, are provided in the Extended Methods in the online supplement and also in our previous work related to environmental risk factors [[41], [42], [43]].

## 3 Results

### 3.1 Effect of different particulate matter preparations

We have developed an exposure protocol to study the effects of noise on the cardiovascular system of mice [41,42]. We first devised a PM exposure protocol for use when investigating the combined effects of noise and PM. Three different PM preparations from the National Institute of Standards and Technology (NIST) were tested in a pilot study. Only the NIST2 PM preparation (urban PM, containing mainly particles with a diameter of 2.5 μm) impaired endothelium-dependent aortic relaxation, increased blood pressure (Fig. 1B and C) and increased the expression of phagocytic NADPH oxidase (NOX-2) (Fig. 1D and E). Interestingly, the blood pressure increase by trend by NIST3 was not accompanied by NOX-2 protein expression upregulation, which may point towards a NOX-2 independent blood pressure increase in our acute exposure model. The aortic expression of the vasoconstrictor endothelin-1 was also increased after exposure to NIST2 PM (Fig. 1F). The other NIST PM preparations we tested did not exhibit comparable changes in any of the determined parameters. Accordingly, all subsequent co-exposure studies were conducted with the NIST2 PM preparation.

### 3.2 Pulmonary oxidative stress and inflammation

**Fig. 2:**
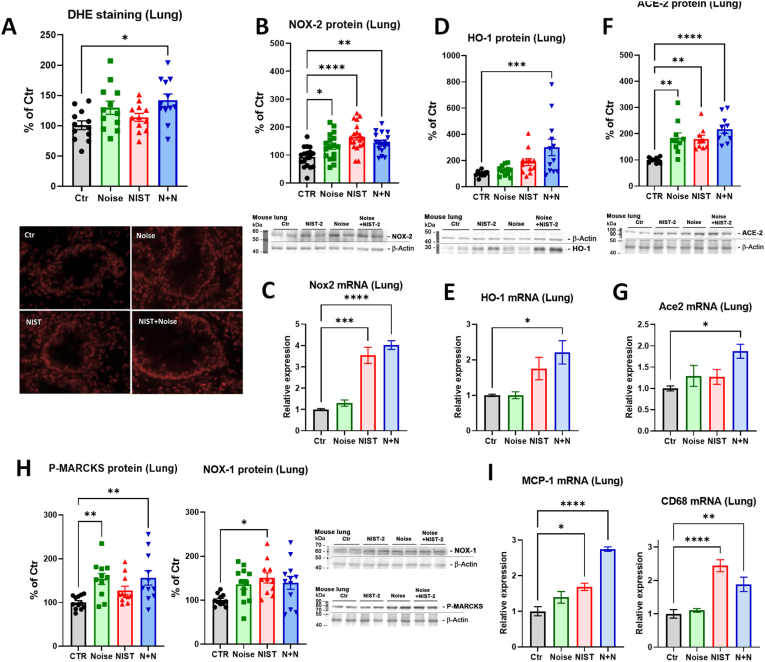
*Effects of PM and noise exposure on oxidative stress in the lungs.* Dihydroethidium fluorescence microtopography in isolated lung sections showed a significant increase in pulmonary oxidative stress for all exposure groups, which was significant for the combined NIST2 PM and noise exposure (A). Representative fluorescence microscope images are shown below the quantification. Protein expression of phagocytic NADPH oxidase subunit, NOX-2, was significantly increased in the in the lung tissue of all three treatment groups, whereas mRNA expression was significantly upregulated by NIST2 PM and the combined exposure (B, C). A clear additive increase was observed for the heme oxygenase-1 (HO-1) in both protein expression and mRNA expression (D, E) Angiotensin converting enzyme 2 (ACE-2) protein and mRNA expression was also additively increased (F, G). Protein expression of phosphorylated myristoylated alanine-rich C-kinase substrate (P-MARCKS) and endothelial NADPH oxidase subunit, Nox1, was upregulated in almost all exposure groups (H), whereas monocyte chemoattractant protein-1 (MCP-1) mRNA expression showed an additive increase and CD68 mRNA expression was exclusively determined by NIST2 PM exposure in the lung (I). Representative original blots are shown besides the quantification. Data are presented as mean ± SEM and individual values are shown. The statistics was performed from n = 12 (A), 12 (B), 3 (C), 17–19 (D), 3 (E), 9–10 (F), 8 (G), 11–12 (H), and 3–6 (I) independent experiments. The statistical significance of p < 0.05 is represented with (*), p < 0.01 is represented with (**), p < 0.001 is represented with (***), and p < 0.0001 is represented with (****).

Previous studies demonstrated that PM exposure increased pulmonary oxidative stress [[44], [45], [46]]. In this study, we exposed animals to both NIST2 PM and to noise to determine distinct and synergistic effects. Oxidative stress in the lung tended to increase in all exposure groups but was significantly increased only in the PM + noise mice (Fig. 2A), which was in line with a significantly increased expression of NOX-2 protein in all exposure groups (Fig. 2B). A more pronounced antioxidant response envisaged by heme oxygenase-1 upregulation was only observed in the PM + noise group (Fig. 2D). Upregulation of *Nox2* and *Hmox1* mRNA was mostly driven by PM exposure alone and was highest in the combined exposure group (Fig. 2C, E). Interestingly, the protein and mRNA expression of pulmonary ACE-2, which represents the docking and entry mechanism for severe acute respiratory syndrome coronavirus 2 (SARS-CoV-2) infection [47] and also plays an important role in vascular health and disease [48], showed an additive increase by PM + noise (Fig. 2F and G). Other markers such as P-MARCKS, a read-out of protein kinase C activity, and Nox1 showed higher protein expression levels in all three exposure groups, whereas the marker of inflammation MCP-1 was additively upregulated by PM + noise exposure and the monocyte/macrophage marker CD68 was mostly regulated by PM exposure alone (Fig. 2H and I).

### 3.3 Effects on blood pressure, aortic vascular function and oxidative stress

**Fig. 3:**
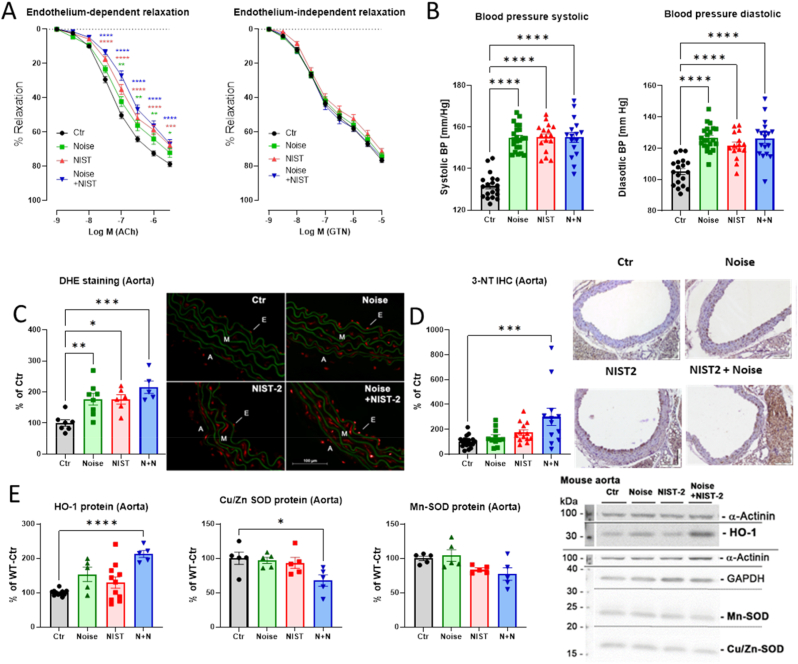
*Effects of PM and noise exposure on aortic conductance vessel function and vascular oxidative stress.* Vascular function was measured in isolated aortic rings (A). The endothelium-dependent relaxation by acetylcholine (ACh) was significantly impaired for all of the treatment groups, however, showing a minor additive impairment in the combined NIST2 PM and noise exposure group. The endothelium-independent relaxation in the presence of nitroglycerin (GTN) did not differ in any of the exposure groups. The systolic and diastolic blood pressure, measured by the tail cuff method, were both significantly increased in all of the exposure groups (B). Dihydroethidium fluorescence microtopography in isolated aortic sections showed a significant increase in oxidative stress in all of the exposure groups, with the combined NIST2 PM and noise exposure showing a minor synergistic effect (C). Representative fluorescence microscope images are shown besides the quantification. The presence of 3-nitrotyrosine positive proteins was measured by immunohistochemistry staining of isolated aortic sections (D). Representative immunohistochemical staining images are shown besides the quantification. The protein expression of the an antioxidant enzymes heme oxygenase-1 (HO-1), Cu,Zn- and Mn-superoxide dismutase (Cu,Zn-SOD and Mn-SOD) in aortic tissue was measured by Western blot (D). Representative original blots are shown besides the quantification. Data are presented as mean ± SEM and individual values are shown where possible. The statistics was performed from n = 19–47 (A), 16–22 (B), 5–8 (C), 11–19 (D), and 5–11 (E) independent experiments. The statistical significance of p < 0.05 is represented with (*), p < 0.01 is represented with (**), p < 0.001 is represented with (***), and p < 0.0001 is represented with (****).

We previously established that exposure to noise alone increased systolic and diastolic blood pressure and endothelial dysfunction in mice [41,42]. The endothelium-dependent relaxation of isolated aortic sections showed an additive impairment upon exposure to PM and noise (Fig. 3A). The endothelium-independent relaxation of isolated aortic sections in the presence of NTG was not modified by any of those exposures. All three exposure groups showed a significant increase in both systolic and diastolic blood pressure, with no additive effects after co-exposure (Fig. 3B). Vascular oxidative stress (as determined by fluorescence of oxidized dihydroethidium products) was increased by the single exposures and showed a trend of additive augmentation in the PM + noise exposed mice (Fig. 3C). Of note, the enhanced ROS formation in aorta seemed to originate from a combination of mitochondrial ROS sources and NADPH oxidase enzymes (suppl. Fig. S3). 3-nitrotyrosine-positive proteins, a marker of nitro-oxidative stress, were significantly higher in aortic tissue of the PM + noise exposed mice than in the aorta of control mice (Fig. 3D). The oxidative stress response protein heme oxygenase-1 was upregulated in an additive manner, whereas the antioxidant enzymes Cu,Zn- and Mn-superoxide dismutase were down-regulated additively in the PM + noise exposure group (Fig. 3E).

### 3.4 Cerebral adverse effects

**Fig. 4:**
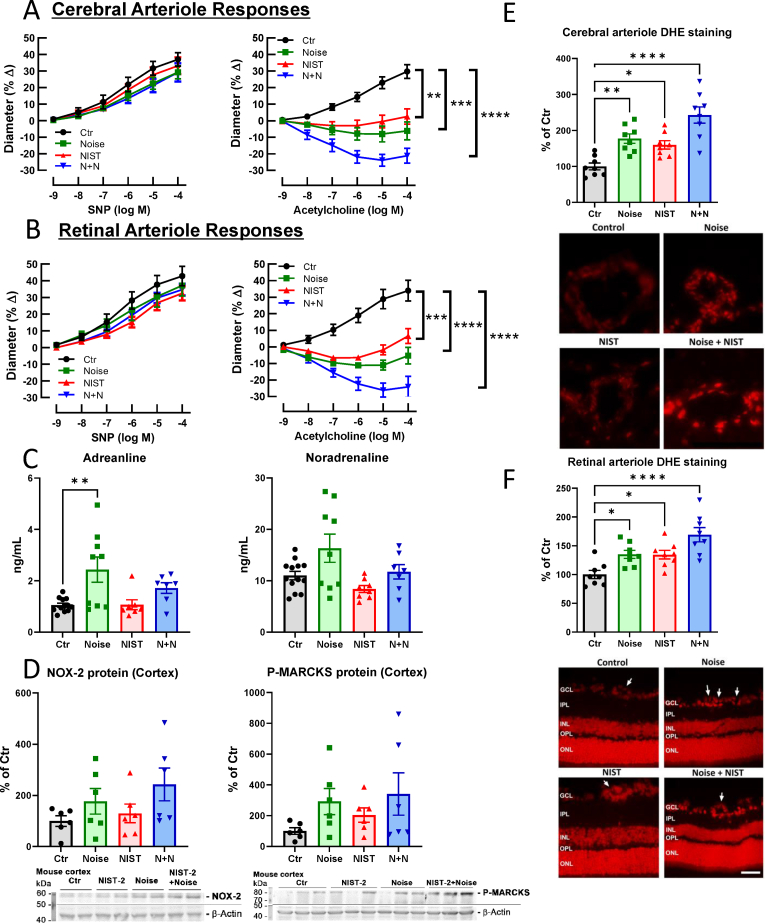
*Effects of PM and noise exposure on arteriolar vascular function and oxidative stress in the brain and stress response hormones.* Vascular function was assessed in cerebral arterioles (A) and in retinal arterioles (B). The arteriole vasodilation was assessed by treatment with the nitric oxide donor sodium nitroprusside (SNP) and the endothelium-dependent vasodilator and endogenous eNOS activator acetylcholine (ACh). Endothelial function (ACh-response) was impaired by the single exposures and additively in the PM-noise combined exposure. The endothelium-independent vasoreactivity to SNP was not affected in any exposure group (for vasoconstriction by U46619 see suppl. Fig. S4). The stress hormones, adrenaline and noradrenaline, were increased in plasma of the noise-exposed animals (C). Protein expression of phosphorylated myristoylated alanine-rich C-kinase substrate (P-MARCKS) and phagocytic NADPH oxidase subunit, NOX-2, was upregulated mostly in the noise-exposed mice (D). Dihydroethidium fluorescence microtopography in sections of cerebral (E), and retinal (F) arterioles (the arrows point to the vascular cross-sections in the retina) showed an increase all exposure groups with displaying additive effect of noise and PM. Representative fluorescence microscope images are shown below the quantification. Representative original blots are shown besides the quantification. Data are presented as mean ± SEM and individual values are shown where possible. The statistics was performed from n = 8 (A), 6 (B), 7–12 (C), 6 (D) and 7 (E, F) independent experiments. The statistical significance of p < 0.05 is represented with (*), p < 0.01 is represented with (**), p < 0.001 is represented with (***), and p < 0.0001 is represented with (****); GCL: ganglion cell layer; IPL: inner plexiform layer; INL: inner nuclear layer; OPL: outer plexiform layer; ONL: outer nuclear layer. The scale bar at (E) is 30 μm and at (F) 50 μm.

We previously demonstrated impairment of cerebral and retinal microvascular function by noise only exposure of mice [49,50]. With the combination of exposures used in this study, we report that endothelium-dependent relaxation of cerebral and retinal arterioles was impaired in an additive manner by PM + noise exposure (Fig. 4A and 4B). Of note, the single exposure to either PM or noise alone induced a significant endothelial dysfunction, but to a lower degree than the combined exposure. In contrast, vasoconstriction by the thromboxane A2 receptor agonist U46619 as well as endothelium-independent vasodilation by sodium nitroprusside, a nitric oxide donor, was not affected by any of the exposures (Fig. 4A and 4B, suppl. Fig. S4). The circulating levels of the stress hormones adrenaline and noradrenaline, as a read-out of sympathetic activation, were elevated most significantly in the noise group and by trend in the PM + noise group; this is likely related to the neuronal perception of noise and the down-stream activation of endocrinal stress responses (Fig. 4C). Likewise, protein expression levels of NOX-2 and P-MARCKS were mostly upregulated by noise alone with a trend of a synergistic increase in the combined exposure group (Fig. 4D). The impaired cerebral and retinal vascular function was accompanied by increased cerebral oxidative stress in all exposure groups with a trend of additive effects in the PM + noise group (Fig. 4E and F).

### 3.5 Changes in circulating cytokines and the cerebral, pulmonary and aortic transcriptome by PM (NIST2) and noise exposure

**Fig. 5:**
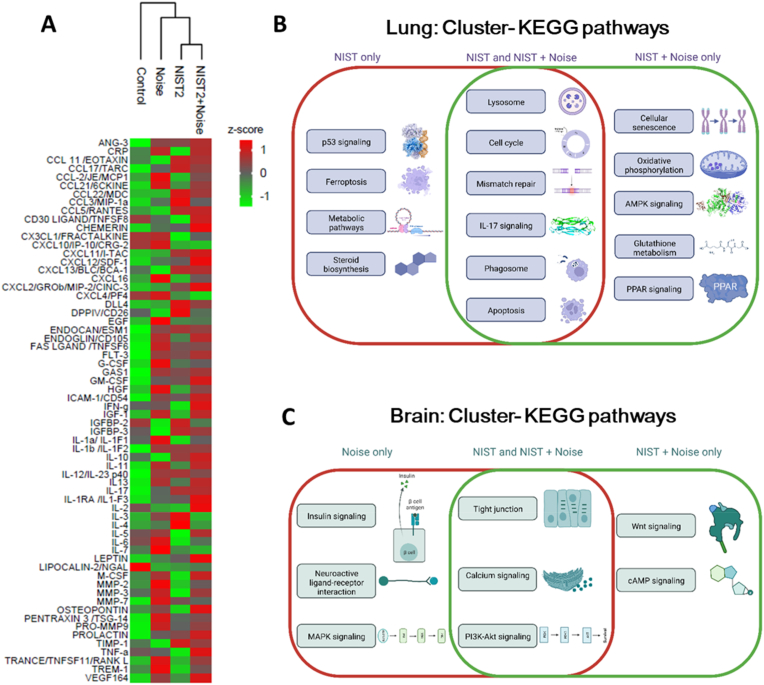
*Circulating cytokine levels and transcriptomic insight into the combined effects of PM and noise exposure.* Heatmap of cytokine profiles identified (A). Heatmap colours indicate scaled cytokine data for the defined conditions (branching of the cluster dendrogram). The intensities were z-scored and are displayed as colours ranging from green to red as shown in the key (n = 3). The most enriched KEGG pathways identified by functional enrichment analysis from an increased cluster with the effect of PM as well as the combined effect of PM + Noise in lung tissue (B, n = 5) and effect of Noise and the combined effect of PM + Noise in the brain (C, n = 5). The overviews of changes in the KEGG pathways are based on protein-protein interaction (PPI) network analyses as described in the online supplement. *P* < 0.05 was considered statistically significant for KEGG analysis. Full detail heat maps and the PPI network analyses of RNA sequencing data are presented in suppl. Figs. S9-S14. Images in B and C were created with BioRender.com. (For interpretation of the references to colour in this figure legend, the reader is referred to the Web version of this article.)

We performed a cytokine array in the plasma of exposed mice to measure the impact of PM and noise on systemic inflammation. The heat map shows a substantial additive upregulation of an appreciable part of the cytokines and chemokines by the combined PM and noise exposure (Fig. 5A). A more detailed quantitative analysis indicated that in addition to the additively regulated cytokines and chemokines, some were only regulated by noise or PM (suppl. Fig. S5). Transcriptome analysis revealed substantial regulation of gene expression by PM and noise exposure and the combination of both in the lung, brain and aorta (see volcano plots in suppl. Figure S6, S7 and S8). We identified significant regulation of specific pathways in the lung, centered around apoptosis, DNA damage, cell cycle, inflammation, lysosome and phagosome as well as antioxidant/metabolic stress response (AMPK, PPAR), mostly by PM but also some additive effects in response to the combined exposure (Fig. 5B). In the brain, the noise exposure alone and the combined exposure showed a significant overlap of the activated pathways, such as changes in the tight junction, and calcium and PI3K-Akt signalling (Fig. 5C).

Detailed cluster and pathway analysis can be found in the online supplement. In the lung, the gene regulatory effects were mostly determined by PM exposure and centered around p53 signaling, steroid biosynthesis, ferroptosis and metabolic pathways for PM alone. Regulation of apoptosis, lysosome, cell cycle, phagosome and mismatch repair and IL-17 signaling were found in the PM alone and PM + noise exposure groups. Changes in oxidative phosphorylation, cellular senescence, glutathione metabolism and antioxidant/metabolic stress response (AMPK, PPAR) were only observed in the PM + noise exposure group (suppl. Figs. S9 and S10). Gene regulatory effects in the brain were mostly determined by noise exposure and centered around neuroactive ligand-receptor interaction, insulin and MAPK signaling for noise alone. PI3K and calcium signaling, tight junction regulation were found in the noise and PM + noise exposure groups. Wnt and cAMP signaling were only observed in the PM + noise exposure group (suppl. Figs. S11 and S12). Noise or PM exposure elicited remarkably distinct gene regulatory effects in the aorta that were centered around PI3K/MAPK/Wnt/Ras/TNF signaling, regulation of lysosome and endocytosis for PM alone whereas regulation of mTOR/HIF-1/PPAR/AMPK signaling, peroxisome, TCA cycle, apoptosis and oxidative phosphorylation were found in the noise alone exposure group suppl. Figs. S13 and S14). Interestingly, only marginal parts of these changes in gene regulation by the single exposures occurred in the combined exposure group.

## 4 Discussion

Our study yields a novel co-exposure model of noise and ambient PM pollution. NIST2 particles were chosen since the exposure to this PM preparation causes significant endothelial dysfunction, oxidative stress and inflammation comparable to that observed with single exposure to aircraft noise [41,42]. Our results revealed that there are in part synergistic negative effects of co-exposure on pulmonary, cardiovascular and cerebral tissues of the exposed mice. The observed increases in in blood pressure were comparable in all exposure groups, while aortic vascular function showed an additive impairment in response to the co-exposure of both environmental stressors. The increase in oxidative stress in pulmonary, vascular and cerebral tissues was associated with increased NOX-2 expression and with the activation of the antioxidant defense, as envisaged by the parallel increase in HO-1 expression. Pulmonary inflammation was also increased, as well as the expression of ACE-2, a known receptor for SARS-CoV-2 virus, but also involved in general processes of cardiovascular health and disease. The RNA sequencing data indicate that noise primarily induces stress responses affecting the brain, while the PM primarily targets the lung, where it causes oxidative damage and inflammation.

### 4.1 Pathophysiology of health side effects in response to a single exposure to noise or air pollution

It is believed that the cognitive perception of noise triggers cortical activation and the release of stress hormones leading to high blood pressure, increased vascular and cerebral inflammation and oxidative stress that in the long turn can enhance cardiovascular risk factors such as diabetes, high cholesterol levels, activation of blood coagulation factors and hypertension that subsequently manifests as cardiovascular disease (CVD, e.g. myocardial infarction, heart failure, persistent hypertension, arrhythmia and stroke) [18,51]. Recent studies indicated a “cerebral” link between noise stimulus, vascular inflammation and adverse cardiovascular events [26] where transportation noise was associated with increased amygdala activity (part of the limbic system, and in general involved in stress perception and control of emotions), vascular inflammation and major adverse cardiovascular events (MACE) [26,52]. Field studies of transportation noise (aircraft and railway) revealed that noise exposure during a single night induces vascular (endothelial) dysfunction, a subclinical parameter for atherosclerosis [53,54], which was partially improved by the acute application of vitamin C (2g p.o.) suggesting an involvement of oxidative stress in inducing endothelial dysfunction [53,54]. Targeted proteomic analysis detected plasma proteins within redox, pro-thrombotic and pro-inflammatory pathways that were significantly impacted in noise-exposed subjects versus controls [54]. Mice exposed to continuous aircraft noise developed high blood pressure, increases in stress hormone levels, and in vascular and cerebral oxidative stress (mainly caused by the phagocytic NADPH oxidase (NOX-2) and by an uncoupled nitric oxide synthase and by increased inflammation caused via immune cell infiltration) phenomena that were not observed in response to comparable sound pressure level of white noise [41]. Of note, aircraft noise-induced vascular and cerebral damage was almost completely prevented by *Nox2* knockout [55] and by heme oxygenase-1 activation [56], pointing to the crucial role inflammatory cells and of oxidative stress in mediating noise-induced cardiovascular and cerebral side effects.

Oxidative stress is also a hall-mark and trigger of the adverse vascular health effects of air pollution [8,57]. Ultrafine (nanometer-sized) particles can directly access the brain via olfactory nerves and cause hypertension by stimulating the HPA axis or mechanoreceptors within the lungs [8]. Larger particles will reach the lungs and the distal parts, the bronchioles, thereby causing inflammation, and depending on the size of the particles, they can transmigrate through the lung epithelium into the bloodstream, and stimulate inflammation in blood vessels and thus trigger the atherosclerotic process by inducing endothelial dysfunction [8].

Receptors, such as the transient receptor potential cation channel subfamily A member 1 (TRPA1) receptors, in airway sensory neurons can also sense environmental toxicants and aerogenic oxidants, resulting in neurogenic inflammation [58]. Activation of the sympathetic nervous system and hypothalamic inflammation occurs in response to PM2.5 exposure with potentiation of blood pressure increases [59]. In general, numerous animal studies have demonstrated an important effect of particulate matter on ROS pathways, while reduction of ROS sources ameliorate blood pressure increases, endothelial dysfunction, impaired ^•^NO bioavailability, endothelial cell activation (adhesion molecule expression) and inflammation [8,57]. Importantly, in chronic PM exposure models most cardiovascular complications were prevented in *Nox2* knockout mice [60] and *p47phox* knockout mice [61]. These pathophysiological mechanisms are also shared in part by other air pollutants such as ozone, nitrogen dioxide and sulfur dioxide with significant health effects as previously reviewed [8,62].

In summary, noise and air pollution share a number of pathophysiological aspects, e.g. the dominant role for reactive oxygen species and inflammation in causing endothelial dysfunction and high blood pressure. We exposed our animals in an experimental approach separately as well as simultaneously to these environmental stressors because noise and air pollution share a number of urban/industrial sources.

### 4.2 Effects of noise/air pollution co-exposure on different organs

Exposure to PM is known to increase pulmonary oxidative stress [[44], [45], [46]] while the effects of transportation noise on respiratory health are rarely studied [63]. This study and others show that noise influences the respiratory system through annoyance and increase in stress hormone release, rather than directly affecting the lung as shown for air pollution [64]. Here, we studied the effects of exposing animals to either NIST2 PM and noise alone or in combination, where oxidative stress tended to increase in all exposure groups, but was significantly enhanced in the co-exposure group only. This fits to the observation that the protein expression of the phagocytic NADPH oxidase (NOX-2) was most pronounced in the combined exposure group only. Upregulation of *Nox2* and *Hmox1* mRNA was mostly driven by PM exposure alone and was highest in the combined exposure group. MCP-1 was also additively upregulated by co-exposure, indicating additive effects of the combination of both stressors on inflammatory processes. Importantly, the protein and mRNA expressions of pulmonary ACE-2, which represents the docking and entry mechanism for severe acute respiratory syndrome coronavirus 2 (SARS-CoV-2) infection [47], showed an additive increase by the co-exposure to PM and noise. This observation can explain at least in part why air pollution is an important cofactor that increases the risk of mortality from COVID-19 as shown by epidemiological studies and atmospheric exposure modeling [65,66]. A recent study has demonstrated ACE-2 expression in lung epithelial cells in response to PM exposure of mice and was suggested to contribute to acute lung injury in this model [67]. In addition to being a receptor for SARS-CoV-2 virus, ACE-2 plays an important role in vascular health and disease [48]. Polymorphism of the ACE-2 D-allele was associated with higher risk of essential hypertension and higher ACE-2 plasma levels were indicative of patients at higher cardiovascular risk [48,68,69]. The role of ACE-2 in the cardiovascular system is somewhat controversial as it is known to stabilize atherosclerotic plaques and lower blood pressure through the conversion of Ang II (vasoconstrictor) into Ang-(1–7) [[70], [71], [72]].

We have previously reported that exposure to noise alone increased systolic and diastolic blood pressure via stimulating the release of neurohormones and by activating the HPA-axis [41,42]. Single exposure to PM2.5 increases blood pressure in mice by activating Toll-like receptor 3 [73]. All three exposure groups showed significant increases in both systolic and diastolic blood pressures, but with no additive effects in the setting of co-exposure. Although we observed additional negative effects on endothelial function, this did not lead to an additional increase in blood pressure. We have to consider that the noise exposure already strongly increased the blood pressure, which may reflect a ceiling effect, so that the addition of air pollution may not result in a further increase. In addition, in case of a sudden increase in blood pressure we encounter multiple counter-regulatory systems in order to prevent further uncontrolled increase of blood pressure. Therefore, it is pretty unusual to observe a really high blood pressure in mice without additional pharmacological intervention or genetic modification respectively, explaining why there is not an additional increase in the case of co-exposure to noise and PM2.5.

In contrast, endothelial dysfunction of conductance (aorta) and resistance vessels (cerebral and retinal arterioles) showed a substantial additive negative effect on vascular function, while vasodilation to the endothelium independent ^•^NO donor sodium nitroprusside was not modified. Vascular oxidative stress as determined by fluorescence of oxidized dihydroethidium products was increased by the single exposures and showed a trend of additive augmentation in the co-exposure group along with higher nitrotyrosine levels. In addition, the oxidative stress response protein, heme oxygenase-1, was upregulated in an additive manner and the expression levels of the antioxidant enzymes Cu,Zn- and Mn-superoxide dismutase were lower in an additive manner. Thus taken together, a higher degree of oxidative stress may be at least in part responsible for the severe degree of endothelial dysfunction in the co-exposure group.

Transportation noise exerts its detrimental health effects to a large extent via the brain, where it initiates stress responses by activating direct and indirect pathways accompanied by increases in circulating catecholamine and cortisol concentrations [74]. Accordingly, human studies demonstrated increased saliva cortisol in noise-exposed populations [75], which is associated with impaired cognitive performance [23], and with neuropsychiatric diseases including depression and anxiety disorders [24] as well as dementia [25]. Particulate matter has also been shown to increase the risk for neuropsychiatric disorders [76] via a direct uptake of (ultra)fine particles via the olfactory nerves contributing to hypertension by stimulating the HPA axis or mechanoreceptors within the lungs [8]. The BREATHE project revealed impairment of cognitive development secondary to exposure to air pollutants [77]. There is emerging evidence for a causal association between PM2.5 pollution and attention-deficit or hyperactivity disorders in children, neurodegenerative diseases, including dementia and stroke [1]. With the present study, we found moderate impairment of cerebral and retinal microvascular function by noise or PM alone, whereas the combined exposure caused substantial additive impairment of microvascular function that was more pronounced than in the large conductance vessel, the thoracic aorta. Microvascular oxidative stress also showed a step-wise increase by single exposure to noise or PM and clear additive exacerbation in response to co-exposure to noise and PM. In contrast, the observed increases in sympathetic stress hormones adrenaline and noradrenaline were almost exclusively mediated by noise exposure and the cerebral (whole brain) NOX-2 expression and activation was almost exclusively noise-dependent. Thus, especially the cerebral microvasculature may be highly sensitive to additive damage by co-exposure to various environmental stressors. This is also supported by the highly detrimental effects of noise alone on the peripheral microvasculature [49].

Previously, we demonstrated significant changes of genes in part responsible for the regulation of vascular function, circadian rhythm, vascular remodeling, and cell death using comparative Illumina sequencing of transcriptomes of aortic tissues from aircraft noise-treated animals [41]. The transcriptomic analysis of murine tissues in our study indicated that if performed as a single exposure, noise and pollution stress respectively induced disparate responses. Importantly, our data also revealed that a co-exposure to both stressors did not induce simple, additive combination responses. We observed that the transcriptional changes in response to both stressors typically generated a non-linear response in combined exposures, including synergy, ablation or the induction of genes unrelated to either, individual stress response.

The lung represents one organ for which we identified possible synergistic negative interactions. We detected a robust induction of pathways related to a DNA-damage response upon PM exposure as exemplified by the activation of p53 signaling, mismatch repair, cell cycle changes and apoptosis. In addition, phagocytosis and lysosomal activation are likely to be induced to clear small particles lodged in the lung tissue. PM clearly induces oxidative stress and inflammation (ferroptosis, IL-17), but also appears to induce metabolic responses. We detected altered signaling in the AMPK pathway, one of the major regulators of cellular energy metabolism. Accordingly, we observed that metabolic pathways downstream of AMPK signaling were also regulated by PM. It included both PPAR signaling, which is a crucial regulator of lipid metabolism, as well as the induction of proteins which are involved in oxidative phosphorylation, or the TCA cycle. In addition, we have identified that glutathione synthesis is induced as a response that is likely due to heightened levels of oxidative stress. Intriguingly, the induction of these PM-induced stress pathways is further exaggerated by noise stress, despite the fact that noise exposure alone causes a nearly completely independent transcriptional response. Transcriptomic analysis in the brain, on the other hand, indicated that the combination of noise and PM exposure elicited a transcriptional response that was comparable to the noise-stress response only, with many of the PM-only dependent transcriptional responses being absent in the combination treatment.

Overall, stressors induced multiple signaling pathways that are of relevance for cardiovascular and neurological diseases. In the brain, the most prominent GO cluster induced by noise or co-exposure included ion transport, *trans*-synaptic signaling and “behavior”, all of which are intimately linked to neuropathological pathways. In the aorta, noise-only exposure triggered the expression of a hypoxic signature. We detected that numerous genes reported to be induced by the hypoxia inducible factor (HIF)-driven transcription were upregulated. This included the induction of genes in the glycolytic pathway, and the downregulation of genes involved in the oxygen-dependent production of ATP, the TCA cycle, lipolysis and the mitochondrial electron transport chain. Suggesting that noise stress may affect the metabolic state of the cardiovascular system, in a similar way to hypoxia. Remarkably, the combination of PM + noise reduced this response, for which we currently have no explanation.

## 5 Conclusions and clinical implications

**Fig. 6:**
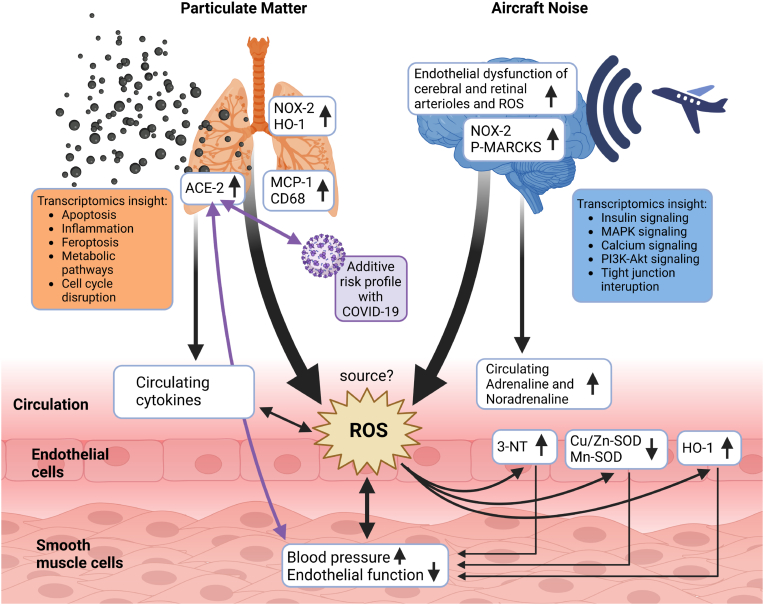
*Proposed pathomechanisms triggered by noise, PM or combined exposure.* While PM confers most of its detrimental effects via damage of the lung (inflammation and oxidative stress), noise initiates systemic adverse health effects primarily by causing neuronal activation, cerebral oxidative stress and stress hormone release. The primary target organ damage by PM and noise converges at the cardiovascular level showing additive exacerbation of some functional (endothelial dysfunction) and biochemical markers (oxidative stress by 3-NT, SODs, HO-1). The source of reactive oxygen species (ROS) in the vasculature was not yet entirely identified but may be a mixture of different NADPH oxidase enzymes and mitochondrial ROS sources. The elevated ACE-2 expression in the lungs of PM and noise co-exposed mice may not only increase the risk and severity of COVID-19 infections but also have general effects on cardiovascular health. Scheme was created using Biorender program.

Previous animal studies have shown additive/synergistic adverse cardiovascular health effects via exaggerated oxidative stress by noise and pre-existing arterial hypertension [78] as well as partially additive or opposite effects on metabolic parameters by altered light exposure and PM [79]. No other study thus far investigated noise and PM co-exposure adverse health effects on a mechanistic basis. Noise and air pollution coexist in many urban/industrial environments [80,81], and therefore should be studied using co-exposure models. Our findings indicate that by investigating one individual stressor at a time, we may significantly underestimate the health risks since noise and air pollution have apparent additive health effects on the cardiovascular system and the brain. Noise and air pollution appear to cause adverse health effects via two main mechanisms. While noise is causing psychological stress, high blood pressure and a strong activation of the neurohormonal system, PM exposure leads primarily to lung damage, mostly mediated by pulmonary inflammation and oxidative stress, which may be propagated to the circulation. The combination of both stressors can lead to additive damage in remote organs and increase the risk and severity of common non-communicable diseases such as diabetes, ischemic heart disease, myocardial infarction, stroke and neurodegeneration (see Fig. 6 for an overview). Of note, the co-exposure to noise and PM leads to an upregulation of ACE-2 (which is associated with an enhanced risk for a severe COVID-19 infection [47] and cardiovascular health effects in general [48]). Future controlled clinical and preclinical co-exposure studies to different environmental risk factors may help to develop a deeper understanding of the underlying mechanisms of the mutual interactions of environmental stressors on health side effects. To date, just few examples exist in the literature demonstrating a cumulative risk of cardiovascular disease and death by co-exposure, e.g. to nocturnal light exposure and air pollution (PM2.5) [82] as well as cumulative risk of diabetes and related cardiovascular complications by road noise and air pollution (ultrafine particles, PM2.5, PM10) [83]. These findings are strongly suggestive of additive/synergistic adverse cardiovascular health effects by environmental stressors that typically co-occur in large cities and urban/industrial settings [30], with a significant contribution to the disease burden and health care costs that may even exceed the most pessimistic scenarios [84].

## Funding

A.D. and T.M. were supported by vascular biology research grants from the 10.13039/501100008454Boehringer Ingelheim Foundation for the collaborative research group “Novel and neglected cardiovascular risk factors: molecular mechanisms and therapeutics” and through continuous research support from Foundation Heart of Mainz (mostly dedicated to the purchase of the exposure system). M.K., I.K., M.T.B.J., K.V.-M. and S.K. hold or held TransMed PhD stipends funded by the 10.13039/501100008454Boehringer Ingelheim Foundation. L.S. and H.U. hold TransMed PhD stipends funded by the Else-Kröner-Fresenius Foundation. S.S. holds an excellence stipend of the Else-Kröner-Fresenius Foundation (2021_EKES.04). T.J. holds a MD stipend funded by the Heart Foundation Mainz. T.M. is PI and A.D. and P.S. and S.D. are (Young) Scientists of the 10.13039/100010447DZHK (German Center for Cardiovascular Research), Partner Site Rhine-Main, Mainz, Germany. Also financial support by 10.13039/501100000289Cancer Research UK (CRUK Edinburgh Centre C157/A255140) and 10.13039/100010269Wellcome Trust (Multiuser Equipment Grant, 208402/Z/17/Z) is gratefully acknowledged. The collaboration of the authors was supported by European 10.13039/501100000921COST Action CA20121: Bench to bedside transition for pharmacological regulation of NRF2 in noncommunicable diseases (BenBedPhar). Webpage: https://benbedphar.org/about-benbedphar/.

## Declaration of competing interest

The authors declare that they have no known competing financial interests or personal relationships that could have appeared to influence the work reported in this paper.

## Data Availability

Data will be made available on request.
