# Supplementary material for: Co-exposure to urban particulate matter and aircraft noise adversely impacts the cerebro-pulmonary-cardiovascular axis in mice
Source: Redox Biol. 2022 Dec 18;59:102580. doi: 10.1016/j.redox.2022.102580 (PMC9804249; doi:10.1016/j.redox.2022.102580)
Supplement: Multimedia component 1 [file mmc1.pdf]

National Institute of Standards & Technology

Certificate of Analysis

Standard Reference Material® 1650b

Diesel Particulate Matter

This Standard Reference Material (SRM) is intended for use in evaluating analytical methods for the determination of selected polycyclic aromatic hydrocarbons (PAHs) and nitro-substituted PAHs (nitro-PAHs) in diesel particulate matter and similar matrices. In addition to certified and non-certified values for selected PAHs and nitro-PAHs, non-certified values are provided for percent extractable mass, particle-size distribution, and specific surface area; supplemental information on mutagenic activity is also provided. All of the chemical constituents for which certified and non-certified values are provided are naturally present in the diesel particulate material. SRM 1650b was prepared from the same bulk diesel particulate material that was issued in 1985 as SRM 1650 [1] and in 2000 as SRM 1650a [2–4]. A unit of SRM 1650b consists of a bottle containing approximately 200 mg of diesel particulate material.

Certified Mass Fraction Values: Certified values are provided for PAHs in Table 1. The certified values for the PAHs are based on the agreement of results obtained at NIST from two or more independent analytical methods [5]. A NIST certified value is a value for which NIST has the highest confidence in its accuracy, in that all known or suspected sources of bias have been investigated or taken into account [5]. Metrological traceability is to the International System of Units (SI) unit of mass expressed as derived unit of mass fraction.

Non-Certified Values: Non-certified mass fraction values are provided for nitro-PAHs in Table 2, additional PAHs in Table 3, for PAHs of molecular mass 302 in Table 4, and for additional nitro-PAHs for specific method conditions in Table 5. In Tables 3 and 5, the non-certified mass fraction values for some PAHs and nitro-PAHs, respectively, are listed more than once depending on the extraction conditions that are used (see "Preparation and Analysis"). A non-certified value for percent extractable mass is provided in Table 6. Non-certified values for specific surface area, as determined by N_2 gas adsorption, and particle-size characteristics are provided in Table 7. Non-certified values are the best estimate of the true value; however, the values do not meet the NIST criteria for certification and are provided with associated uncertainties that may reflect only measurement precision, may not include all sources of uncertainty, or may reflect a lack of sufficient statistical agreement among multiple analytical methods [5]. Non-certified values are traceable to the measurement processes and standards used by NIST.

Period of Validity: SRM 1650b is valid, with the measurement uncertainty specified, until **01 May 2031**, provided the SRM is handled and stored in accordance with instructions given in this certificate (see "Instructions for Use"). The value assignments are nullified if the SRM is damaged, contaminated, or otherwise modified.

Maintenance of SRM Certification: NIST will monitor this SRM over the period of its validity. If substantive technical changes occur that affect the certification before the expiration of this certificate, NIST will notify the purchaser. Registration (see attached sheet or register online) will facilitate notification.

Coordination of the technical measurements leading to the certification of SRM 1650b was under the leadership of S.A. Wise of the NIST Chemical Sciences Division and M.M. Schantz, formerly of NIST.

Statistical consultation was provided by N.A. Heckert of the NIST Statistical Engineering Division and S.D. Leigh, formerly of NIST.

Carlos A. Gonzalez, Chief
Chemical Sciences Division

Gaithersburg, MD 20899
Certificate Issue Date: 07 July 2021
Certificate Revision History on Last Page

Steven J. Choquette, Director
Office of Reference Materials

Analytical measurements for the certification of SRM 1650b were performed by D.L. Poster of the NIST Material Measurement Laboratory Office and H.A. Bamford, B.J. Porter, M.M. Schantz, P. Schubert, and R. Zeisler, formerly of NIST. Analytical measurements for PAHs and nitro-PAHs were also provided by C. Chiu of the Environmental Technology Centre, Environment Canada (Ottawa, Canada). Specific surface area and porosity measurements and confirmation measurements for 1-nitropyrene were provided by P. Scheepers of the Department of Epidemiology at Katholieke Universiteit Nijmegen (Nijmegen, The Netherlands).

Support aspects involved in the issuance of this SRM were coordinated through the NIST Office of Reference Materials.

INSTRUCTIONS FOR HANDLING, STORAGE, AND USE

Handling: This material is a naturally occurring diesel particulate material and contains constituents of known and unknown toxicities and mutagenicities; therefore, extreme caution and care should be exercised during its handling and use.

Storage: Store SRM 1650b in its original bottle at approximately 25 ºC (room temperature) and keep away from direct sunlight.

Use: Prior to removal of subsamples for analysis, the contents of the bottle should be mixed thoroughly. The recommended minimum sample size is 50 mg, although smaller sample sizes have been evaluated. The evaluation of the homogeneity of SRM 1650b, at small sample sizes for PAHs, is described in "Homogeneity Assessment for PAHs" and in further detail in reference 6.

PREPARATION AND ANALYSIS(1)

Sample Collection and Preparation: The diesel particulate material used for the preparation of SRM 1650b was the same bulk diesel particulate material used for the preparation of SRM 1650 and SRM 1650a. This material was obtained through the Coordinating Research Council, Inc. (Atlanta, GA). The particulate material was collected from the heat exchangers of a dilution tube facility following 200 engine hours of particulate accumulation. Several direct injection four-cycle diesel engines, operating under a variety of conditions were used to generate this particulate material. Therefore, while the sample is not intended to be representative of any particular diesel engine operating under any specific conditions, it should be representative of heavy-duty diesel engine particulate emissions.

Relationship Among SRM 1650, SRM 1650a, and SRM 1650b: SRM 1650b was prepared from the same bulk diesel particulate matter used for preparation of SRM 1650; the bulk material had been stored at –20 °C since the preparation of SRM 1650 in 1984. SRM 1650b was analyzed as described below to confirm that the bulk diesel particulate matter was the same as the material used for SRM 1650 and SRM 1650a. The analyses of SRM 1650b confirmed that the material was the same and that the mass fractions of PAHs and nitro-PAHs had not significantly changed. The results of these analyses were then used to assign new certified and non-certified mass fractions and to increase again the number of PAHs and nitro-PAHs with values assigned. However, measurements for some properties (e.g., percent extractable mass and pore size) and some PAHs (selected methyl- and dimethyl-substituted PAHs) were not repeated on SRM 1650b; instead it was determined that the information values from SRM 1650a are applicable to SRM 1650b.

Polycyclic Aromatic Hydrocarbons (PAHs)

The general approach used for the value assignment of the PAHs in SRM 1650b consisted of pressurized fluid extraction (PFE) using dichloromethane (DCM), toluene, or a toluene/methanol mixture at two extraction temperatures (100 °C and 200 °C), cleanup of the extracts using solid phase extraction (SPE), and analysis by using gas chromatography/mass spectrometry (GC/MS) on three stationary phases of different selectivity [i.e., a relatively nonpolar phase, a 50 % (mole fraction) phenyl-substituted methylpolysiloxane phase, and a dimethyl 50 % (mole fraction) polysiloxane liquid crystalline stationary phase]. PFE was the only extraction method used at NIST; previous studies indicated that conventional Soxhlet extraction was not as effective as PFE in the removal of higher relative molecular mass PAHs [7]. Results were also obtained from Environment Canada as described in "GC/MS (Environment Canada)".

---

(1)Certain commercial equipment, instruments or materials are identified in this certificate to adequately specify the experimental procedure. Such identification does not imply recommendation or endorsement by the National Institute of Standards and Technology, nor does it imply that the materials or equipment identified are necessarily the best available for the purpose.

Multiple sets of GC/MS results performed by the NIST Chemical Sciences Division, designated as GC/MS (Ia and Ib), GC/MS (IIa and IIb), GC/MS (IIIa and IIIb), GC/MS (IVa and IVb), GC/MS (Va and Vb), GC/MS (VIa and VIb), GC/MS (VII), GC/MS (VIII) and GC/MS (Environment Canada), were obtained using three columns with different selectivities for the separation of PAHs. For all PAH analyses, the mass spectrometer was operated using electron impact ionization.

GC/MS (Ia and Ib): For GC/MS (I) analyses, duplicate subsamples of 50 mg from six bottles of SRM 1650b were mixed with Hydromatrix (Isco, Lincoln, NE) and extracted with toluene using PFE at 100 °C and 13.8 MPa as described by Schantz et al. [7]. The extracts were concentrated to about 0.5 mL, solvent exchanged to hexane, placed on an aminopropylsilane SPE cartridge, and eluted with 20 mL of 2 % DCM in hexane (volume fraction). The eluant was concentrated and then analyzed by using GC/MS with a 0.25 mm i.d. × 60 m fused silica capillary column with a relatively nonpolar phase (0.25 µm film thickness; DB-XLB, J&W Scientific, Folsom, CA) and these results are denoted as GC/MS (Ia). Concentrated eluants were also analyzed on a 50 % phenyl-substituted methylpolysiloxane stationary phase (0.25 mm i.d. × 60 m, 0.25 µm film thickness; DB-17MS, J&W Scientific), and these results are designated as GC/MS (Ib).

GC/MS (IIa and IIb): For the GC/MS (IIa and IIb) analyses, 50 mg samples from six bottles of SRM 1650b were extracted with toluene using PFE at 200 °C and 13.8 MPa. The extracts were processed and analyzed as described above for GC/MS (Ia) and GC/MS (Ib) and the results are designated GC/MS (IIa) and GC/MS (IIb), respectively.

GC/MS (IIIa and IIIb): For GC/MS (IIIa and IIIb) analyses, subsamples of 50 mg to 100 mg from six bottles of SRM 1650b were mixed with clean sodium sulfate and extracted with DCM using PFE at 100 °C and 13.8 MPa. The extracts were processed as described above for GC/MS (Ia and Ib) and then analyzed by using GC/MS on fused silica capillary columns with a 50 % phenyl-substituted methylpolysiloxane stationary phase (0.25 mm i.d. × 60 m, 0.25 µm film thickness), designated as GC/MS (IIIa), and on a dimethyl 50 % polysiloxane liquid crystalline stationary phase (0.25 mm i.d. × 15 m, 0.25 µm film thickness; LC-50, J&K Environmental, Milton, Ontario, Canada), designated as GC/MS (IIIb).

GC/MS (IVa and IVb): For GC/MS (IVa and IVb) analyses, subsamples of 50 mg to 100 mg from six bottles of SRM 1650b were extracted with DCM using PFE at 200 °C and 13.8 MPa. The extracts were processed and analyzed by using GC/MS on the two columns as described above for GC/MS (IIIa and IIIb).

GC/MS (Va and Vb) and GC/MS (VIa and VIb): Additional extractions and analyses were performed in the same manner as for GC/MS (IIIa and IIIb) and GC/MS (IVa and IVb) to obtain concentrations for selected methylated PAHs. These data sets are designated as GC/MS (Va and Vb) and GC/MS (VIa and VIb).

GC/MS (VII): For the relative molecular mass (M_r) 276 and higher PAHs, additional extractions and analyses were performed in a similar manner as for GC/MS (Ib) except that the PAHs of interest were eluted from the aminopropylsilane SPE cartridge using 40 mL of 10 % DCM in hexane (volume fraction). These data are designated as GC/MS (VII).

GC/MS (VIIIa through VIIId): The effect of increasing the temperature and pressure used for PFE on the extraction efficiency for PAHs was evaluated. The solvent used was toluene, although a 9:1 toluene:methanol (volume fraction) was also evaluated. The PFE conditions used included: 100 °C with 13.8 MPa; 100 °C with 20.7 MPa; 200 °C with 13.8 MPa; and 200 °C with 20.7 MPa, methods GC/MS (VIIIa) through GC/MS (VIIId), respectively. Following an SPE step similar to that of method I above, the processed extracts were analyzed using a non-polar, extra-low bleed phase (0.25 mm i.d. × 60 m, 0.25 µm film thickness).

GC/MS Internal Standards: For all GC/MS measurements described above, except GC/MS (VIa and VIb), selected perdeuterated PAHs were added to the diesel particulate matter prior to extraction for use as internal standards for quantification purposes. For GC/MS (VIa and VIb), fluorinated PAHs were added to the diesel particulate matter prior to extraction for use as internal standards for quantification purposes.

GC/MS (Environment Canada): For PAH measurements at Environment Canada, subsamples of approximately 10 mg from each of four bottles of SRM 1650b were extracted with DCM or hexane/acetone (1:1 volume fraction) using microwave-assisted extraction at 100 °C for 20 min. The extract was filtered through sodium sulfate and concentrated to a few milliliters by rotary evaporation, solvent exchanged to cyclohexane, and then divided into two portions; one portion was processed for PAH measurements and the other portion was processed for nitro-PAH measurements (see below). The PAH fraction was isolated from the extract by using open-column chromatography on silica, with hexane followed by benzene as the mobile phase. The PAH fraction was then analyzed by using GC/MS on a DB-XLB column (0.25 mm i.d. × 30 m, 0.25 µm film thickness). Selected perdeuterated PAHs were added to the diesel particulate matter prior to extraction for use as internal standards for quantification purposes.

PAH Isomers of Relative Molecular Mass (M_r) *302*: For the determination of the relative molecular mass (M_r) 302 isomers, the method used was similar to that described by Schubert et al. [8]. Two sets of samples (one set of three subsamples and one set of six subsamples) of approximately 50 mg each were extracted using PFE at 100 °C and 13.8 MPa with DCM. The extracts were then concentrated, solvent exchanged to hexane, passed through an aminopropylsilane SPE cartridge, and eluted with 40 mL of 10 % DCM in hexane (volume fraction). The processed extract was then analyzed by GC/MS using a 0.25 mm i.d. × 60 m fused silica capillary column with a 50 % phenyl-substituted methylpolysiloxane phase (0.25 µm film thickness; DB-17MS). Perdeuterated dibenzo[*a,i*]pyrene was added to the diesel particulate matter, prior to extraction, for use as an internal standard.

Homogeneity Assessment for PAHs: The homogeneity of SRM 1650b was assessed by analyzing duplicate samples of 50 mg from six bottles selected by stratified random sampling. Samples were processed and analyzed as described above for GC/MS (I). No statistically significant differences among bottles were observed for the PAHs at the 50 mg sample size. The relative homogeneity of trace levels of PAHs in SRM 1650b in the milligram sampling range was also evaluated. The subsampling contribution to the overall uncertainty varies with PAH considered. Using pyrene as an example, the subsampling error for a 10 mg sample size of SRM 1650b is 2 %, while the subsampling error for a 50 mg sample of SRM 1650b is 0.9 %. A more extensive evaluation of the homogeneity of SRM 1650b, at small sample sizes for PAHs, is described in reference 6.

Nitro-Substituted Polycyclic Aromatic Hydrocarbons (Nitro-PAHs)

SRM 1650b was analyzed at NIST and Environment Canada for the determination of nitro-PAHs. The general procedure for determination of nitro-PAHs at NIST utilizes GC with negative ion chemical ionization mass spectrometry (GC/NICI-MS) [9,10] and high-resolution mass spectrometry using negative chemical ionization (GC/NCI-HRMS). Mass fraction values for nitro-PAHs are provided in Tables 2 and 5.

GC/NICI-MS (I): Subsamples of approximately 50 mg from each of six bottles of SRM 1650b were mixed with Hydromatrix (Isco, Lincoln, NE) and extracted with DCM using PFE at 100 °C and 13.8 MPa. The extracts were concentrated to about 0.5 mL, solvent exchanged to hexane, placed on an aminopropylsilane SPE cartridge, and eluted with 40 mL of 20 % DCM in hexane (volume fraction). To isolate the nitro-PAH fraction, the concentrated eluant was analyzed by normal-phase liquid chromatography (LC) using a semi-preparative amino/cyano phase column with a mobile phase of 20 % DCM in hexane [10]. The nitro-PAH fraction was analyzed by GC with GC/NICI-MS using a 0.25 mm i.d. × 30 m fused silica capillary column containing a 50 % phenyl-substituted methylpolysiloxane stationary phase (0.25 µm film thickness), and the results are designated as GC/NICI-MS (I).

GC/NICI-MS (II): A second set of four subsamples of SRM 1650b was processed and analyzed at a different time using the same procedures described above and the results are denoted as GC/NICI-MS (II).

GC/NICI-MS (IIIa through IIId): The effect of increasing the temperature and pressure used for PFE on the extraction efficiency for nitro-PAHs was evaluated. The solvent used was toluene, although a 9:1 toluene:methanol (volume fraction) was also evaluated. The PFE conditions used included: 100 °C with 13.8 MPa; 100 °C with 20.7 MPa; 200 °C with 13.8 MPa; and 200 °C with 20.7 MPa [methods GC/NICI-MS (IIIa) through GC/NICI-MS (IIId), respectively]. Following an SPE step similar to that of method I above, the processed extracts were analyzed using a non-polar, extra-low bleed proprietary phase (0.25 mm i.d. × 60 m, 0.25 µm film thickness).

GC/NCI-HRMS: For the Environment Canada measurements of nitro-PAHs, five subsamples of SRM 1650b were extracted as described above for PAH measurements. The second portion of the extract was taken to dryness and then redissolved in 1 mL of dimethyl sulfoxide (DMSO). The nitro-PAH fraction was isolated by using a liquid-liquid partition scheme involving hexane extraction of the DMSO to remove aliphatic hydrocarbons, followed by dilution of the DMSO with water and extraction of the polar fraction into cyclohexane. The nitro-PAHs were then isolated by using normal-phase LC on a silica column with a solvent gradient from 5 % DCM in hexane (volume fraction) to 100 % DCM. The nitro-PAH fraction was collected from 60 % DCM in hexane to 100 % DCM, concentrated, and analyzed by using GC on a 30 m 5 % phenyl-substituted methylpolysiloxane column (0.25 mm i.d., 0.25 µm film thickness) with detection by high-resolution mass spectrometry using negative chemical ionization (GC/NCI-HRMS).

GC/NICI-MS and GC/NCI-HRMS Internal Standards: For the GC/NICI-MS and GC/NCI-HRMS measurements described above, perdeuterated nitro-PAHs were added to the diesel particulate matter, prior to extraction, for use as internal standards for quantification purposes.

Value Assignment for PAHs and Nitro-PAHs

The value assignment of PAHs and nitro-PAHs in SRM 1650b is based on the measurements used when SRM 1650b was issued in 2003 and on recent additional measurements using different extraction temperatures. Recent studies on the extraction of PAHs and nitro-PAHs from diesel particulate matter [11–13] have shown that using PFE at 200 °C removes higher quantities of some PAHs and nitro-PAHs than using PFE at 100 °C. As a result of these studies, value assignment for specific PAHs and nitro-PAHs in SRM 1650b is based on measurements using PFE at both 100 °C and 200 °C. In cases where the quantities of the individual PAHs and nitro-PAHs do not change with the PFE temperature, the measurements were combined and the resulting values are denoted as certified values and non-certified values in Tables 1 and 2, respectively. When different results are obtained at 100 °C and 200 °C, the values are reported for both temperatures, and they are denoted as non-certified values in Tables 3 and 5. These non-certified values should be considered "method dependent" values, because they are dependent on the extraction method and temperature.

Percent Extractable Mass

For the determination of percent extractable mass, six subsamples of approximately 200 mg were extracted using Soxhlet extraction for 18 h with DCM. The extract was concentrated to approximately 20 mL and then filtered to remove particulate matter. Aliquots of 100 µL to 150 µL were placed in tared aluminum foil pans; the DCM was evaporated until constant mass was obtained, and then the mass of the residue remaining was determined. The percent extractable mass non-certified value for SRM 1650a is applicable to SRM 1650b and provided in Table 6.

Particle Size Information, Specific Surface Area, and Porosity

Particle-size distribution measurements for SRM 1650b were carried out using a laser diffraction instrument (Mastersizer 2000, Malvern Instruments, Southborough, MA) set at a refractive index of 1.5 and absorption index of 0.1 and the liquid suspension method with the instrument manufacturer's small volume sample dispersion unit (Hydro 2000 SM). A suspension of 0.1 % (mass fraction) of SRM 1650b in distilled water with 0.001 % Triton (volume fraction) was prepared by ultra-sonication for 1 h and 24 h. After the recording of the background, a portion of the suspension was added to the measurement cell to achieve an obscuration of 5 %. Three passes of the sample solution were recorded and averaged. Results were calculated using the General Purpose Model provided by the instrument manufacturer; the results obtained for the two sonication periods are shown in Figure 1. The diesel particulate matter, as-received, does not have a stable particle size because of agglomeration which is evident, as the particle-size distribution for SRM 1650b measured after the 1 h sonication period did not show a typical profile for a material from a combustion process. The subsequent 24 h sonication broke up most agglomerates, and the size distribution shows a profile typical for combustion engine emissions (see Table 7).

The specific surface area and porosity were determined based on N_2 gas adsorption measurements [14]. The gas adsorption measurements were performed on a NOVA-1200 instrument (Quantachrome Corp., Boynton Beach, FL) at 77 K after the samples were outgassed for 24 h at 120 °C under vacuum. The N_2 isotherms were analyzed using the Brunauer-Emmet-Teller (BET) equation [15] to obtain the surface area (Table 7) and the Barrett-Joyner-Halenda (BJH) method [16] to obtain the porosity. Based on the BJH method, SRM 1650b shows a wide distribution of mesopores, but with substantial outer area. The pore diameter of the particles in SRM 1650b ranges from 4 nm to 45 nm with a mean at about 25 nm.

Supplemental Information for SRM 1650b

Because SRM 1650, SRM 1650a, and SRM 1650b were all prepared from the same bulk diesel particulate matter, some measurements reported for the previous two materials have not been duplicated for SRM 1650b and the previous results are transferable to the current SRM 1650b. In addition, because SRM 1650 and SRM 1650a have been available since 1985, a considerable amount of information on the characterization of these materials has been published. A summary of some of the studies reporting characterization of this diesel particulate matter SRM is provided in Poster et al [3,4]. A description of the mutagenicity assay is provided below as supplemental information for SRM 1650b.

Mutagenicity Activity: Non-certified values for the mutagenic activity of a dichloromethane extract of SRM 1650 were determined as part of an international collaborative study in 1989 sponsored by the International Programme on Chemical Safety (IPCS) and supported and technically coordinated by the U.S. Environmental Protection Agency's (EPA) Office of Health Research. Twenty laboratories from North America, Europe, and Japan participated in the study for which a complete summary has been published [17,18]. Mutagenicity data were provided by J. Lewtas and L.D. Claxton of the National Health and Environmental Effects Research Laboratory, U.S. EPA (Research Triangle Park, NC).

Table 1. Certified Mass Fraction Values for PAHs in SRM 1650b

PAH	Mass Fraction[(a)] (mg/kg)		
Phenanthrene[(b,c,d,e,f,g,h,i,j,k)]	65.6	±	3.6
1-Methylphenanthrene[(b,c,d,e,k,l,m)]	32.1	±	1.4
2-Methylphenanthrene[(b,c,d,e,k,l,m)]	72.3	±	1.2
3-Methylphenanthrene[(b,c,d,e,k,l,m)]	56.7	±	1.9
9-Methylphenanthrene[(b,c,d,e,k,l,m)]	36.6	±	1.6
Fluoranthene[(b,c,d,e,f,g,h,i,j,k)]	48.1	±	1.1[(n)]
Pyrene[(b,c,d,e,f,g,h,i,j,k)]	44.1	±	1.2
Benzo[*ghi*]fluoranthene[(c,e,f,g,h,i,j,k)]	11.1	±	0.7
Benzo[*c*]phenanthrene[(b,c,d,e,f,g,h,i,k)]	2.65	±	0.24
Benz[*a*]anthracene[(b,c,d,e,f,g,h,i,j,k)]	6.45	±	0.39
Chrysene[(b,d,g,o)]	13.4	±	0.6
Triphenylene[(b,d,g,o)]	9.49	±	0.63
Benzo[*a*]fluoranthene[(b,c,d,e,f,h,k)]	0.384	±	0.023
Benzo[*b*]fluoranthene[(c,e,f,g,h,i)]	6.77	±	0.92
Benzo[*j*]fluoranthene[(c,e,f,h)]	3.24	±	0.50[(n)]
Benzo[*k*]fluoranthene[(b,c,d,e,f,g,h,i,j,k)]	2.30	±	0.18[(n)]
Benzo[*e*]pyrene[(b,c,d,e,f,g,h,i,j,k)]	6.36	±	0.37
Benzo[*a*]pyrene[(b,c,d,e,f,g,h,i,j,k)]	1.25	±	0.12
Perylene[(c,d,e,k)]	0.167	±	0.019[(n)]
Indeno[1,2,3-*cd*]pyrene[(b,c,d,e,f,g,h,i,j,k,o)]	4.48	±	0.21
Benzo[*ghi*]perylene[(b,c,d,e,f,g,h,i,j,k,o)]	6.04	±	0.30
Dibenz[*a,c*]anthracene[(c,e,f,g,h,i)]	0.439	±	0.048
Dibenz[*a,h*]anthracene[(c,e,g,i)]	0.365	±	0.082
Dibenz[*a,j*]anthracene[(c,e,g,i)]	0.387	±	0.068[(n)]
Benzo[*b*]chrysene[(b,c,d,e,f,g,h,i,k)]	0.301	±	0.019[(n)]
Picene[(b,c,d,e,f,g,h,i,k)]	0.506	±	0.058[(n)]

(a) The certified mass fraction values, unless otherwise footnoted, are weighted means of the mass fractions from multiple analytical methods [19]. The uncertainty listed with each value is an expanded uncertainty about the mean [19,20] with coverage factor, $k = 2$, calculated by combining within-method variances with a between-method variance [21] following the ISO/JCGM Guide [22,23].
(b) GC/MS (Ia) on a proprietary relatively nonpolar phase after PFE at 100 °C and 13.8 MPa with toluene.
(c) GC/MS (Ib) on 50 % phenyl-substituted methylpolysiloxane phase, same extract as GC/MS (Ia).
(d) GC/MS (IIa) on a proprietary relatively nonpolar phase after PFE at 200 °C and 13.8 MPa with toluene.
(e) GC/MS (IIb) on 50 % phenyl-substituted methylpolysiloxane phase same extract as GC/MS (IIa).
(f) GC/MS (IIIa) on 50 % phenyl-substituted methylpolysiloxane phase after PFE at 100 °C and 13.8 MPa with DCM.
(g) GC/MS (IIIb) on dimethyl 50 % polysiloxane liquid crystalline stationary phase with same extract as GC/MS (IIIa).
(h) GC/MS (IVa) on 50 % phenyl-substituted methylpolysiloxane phase after PFE at 200 °C and 13.8 MPa with DCM.
(i) GC/MS (IVb) on dimethyl 50 % polysiloxane liquid crystalline stationary phase with same extract as GC/MS (IVa).
(j) GC/MS (Environment Canada) on a proprietary relatively nonpolar phase.
(k) GC/MS (VIII) on non-polar extra low bleed proprietary phase after PFE with toluene at 100 °C with 13.8 MPa; 100 °C with 20.7 MPa; 200 °C with 13.8 MPa; and 200 °C with 20.7 MPa.
(l) GC/MS (Va) on 50 % phenyl-substituted methylpolysiloxane phase after PFE at 100 °C and 13.8 MPa with DCM.
(m) GC/MS (VIa) on 50 % phenyl-substituted methylpolysiloxane phase after PFE at 200 °C and 13.8 MPa with DCM.
(n) The certified value is a weighted mean of average mass fractions, with one average from each of two or more analytical methods [19,20]. The expanded uncertainty is the half width of a symmetric 95 % parametric bootstrap confidence interval [24] which is consistent with the ISO/JCGM Guide [22,23] with an effective coverage factor, k, equals 2.
(o) GC/MS (VII) on 50 % phenyl-substituted methylpolysiloxane phase after PFE at 100 °C with DCM.

Table 2. Non-Certified Mass Fraction Values for Nitro-PAHs in SRM 1650b

Nitro-PAH	Mass Fraction[a] (µg/kg)
9-Nitrophenanthrene[b,c,d,e]	539 ± 24
3-Nitrophenanthrene[b,c,e]	4250 ± 50[f]
2-Nitrofluoranthene[b,c,e]	217 ± 15
3-Nitrofluoranthene[b,c,e]	65.1 ± 1.1
1-Nitropyrene[b,c,e,g]	18400 ± 300
7-Nitrobenz[*a*]anthracene[b,c,e]	943 ± 22[f]
6-Nitrochrysene[b,c,e]	46.6 ± 1.1[f]

[a] The non-certified mass fraction values, unless otherwise footnoted, are weighted means of the mass fractions from multiple analytical methods [19]. The uncertainty listed with each value is an expanded uncertainty about the mean [19,20] with coverage factor, $k = 2$, calculated by combining within-method variances with a between-method variance [21] following the ISO/JCGM Guide [22,23].
[b] GC/NICI-MS (I) on 50 % phenyl-substituted methylpolysiloxane stationary phase.
[c] GC/NICI-MS (II) on 50 % phenyl-substituted methylpolysiloxane stationary phase.
[d] GC/NCI-HRMS on 5 % phenyl-substituted methylpolysiloxane stationary phase.
[e] GC/NICI-MS (III) on 50 % phenyl-substituted methylpolysiloxane phase after PFE with toluene at two temperatures and two pressures (100 °C with 13.8 MPa; 100 °C with 20.7 MPa; 200 °C with 13.8 MPa; and 200 °C with 20.7 MPa).
[f] The non-certified value is a weighted mean of average mass fractions, with one average from each of two or more analytical methods [19,20]. The expanded uncertainty is the half width of a symmetric 95 % parametric bootstrap confidence interval [24], which is consistent with the ISO/JCGM Guide [22,23] with an effective coverage factor, k, equals 2.
[g] Non-certified value for 1-nitropyrene confirmed original SRM 1650 certified value, which included measurements by two other independent techniques [2,25].

Table 3. Non-Certified Mass Fraction Values for PAHs in SRM 1650b
Based on Extraction Method and Conditions

Extraction Conditions	Mass Fractions (mg/kg)[a]
PFE at temperatures between 100 °C and 200 °C	
1-Methylnaphthalene[b,c,d,e,f]	1.71 ± 0.26[g]
2-Methylnaphthalene[b,c,d,e,f]	3.71 ± 0.21
Acenaphthene[c,e,f,h]	0.233 ± 0.021
1,7-Dimethylphenanthrene[e,f,i,j]	17.2 ± 0.7
4H-Cyclopenta[*def*]phenanthrene[e,f]	3.35 ± 0.18
Cyclopenta[*def*]phenanthrone[k,l]	15.6 ± 0.8
Cyclopenta[*cd*]pyrene[b,h]	0.349 ± 0.068
1-Methylfluoranthene[e,f,i,j,m]	3.17 ± 0.11
3-Methylfluoranthene[e,f,i,j,m]	6.09 ± 0.83
1-Methylpyrene[e,f,i,j]	2.05 ± 0.14
3-Methylchrysene[e,f,i,j,n,o]	2.17 ± 0.10
6-Methylchrysene[e,f,i,j,n,o]	1.57 ± 0.04[g]
Coronene[e,f,p]	9.48 ± 0.28

Extraction Conditions		Mass Fractions (mg/kg)[(a)]
PFE at 100 °C		
	Naphthalene[(e,h,q)]	5.16 ± 0.48[(g)]
	Biphenyl[(e,h,k,q)]	0.965 ± 0.035
	Acenaphthylene[(c,e,q)]	0.365 ± 0.019
	Fluorene[(e,h,k,q)]	0.762 ± 0.029
	Anthracene[(e,h,k,q,r,s)]	1.56 ± 0.20[(g)]
	2-Methylanthracene[(h,q)]	0.60 ± 0.11
	Dibenzothiophene[(e,h,q)]	9.54 ± 0.56
	1,2-Dimethylphenanthrene[(t)]	6.3 ± 0.5
	1,6-, 2,5-, and 2,9-Dimethylphenanthrene[(t)]	38 ± 3
	1,8-Dimethylphenanthrene[(t)]	4.5 ± 0.5
	2,6-Dimethylphenanthrene[(t)]	29 ± 2
	2,7-Dimethylphenanthrene[(t)]	20 ± 2
	3,6-Dimethylphenanthrene[(t)]	23 ± 2
	8-Methylfluoranthene[(i,j)]	3.60 ± 0.12[(g)]
	2-Methylpyrene[(i,j,n,o)]	5.8 ± 1.4
	4-Methylpyrene[(i,j,n)]	5.14 ± 0.63
	1-Methylchrysene[(n,o)]	1.46 ± 0.05
	2-Methylchrysene[(i,j,n,o)]	2.50 ± 0.26[(g)]
	1-Methylbenz[*a*]anthracene[(o)]	0.355 ± 0.010
	2-Methylbenz[*a*]anthracene[(i,j)]	0.43 ± 0.19
	6-Methylbenz[*a*]anthracene[(o)]	5.28 ± 0.17
	9- and 3-Methylbenz[*a*]anthracene[(i,j)]	0.61 ± 0.17
	11-Methylbenz[*a*]anthracene[(i,j)]	0.36 ± 0.15
PFE at 200 °C		
	Naphthalene[(f)]	7.29 ± 0.38
	Biphenyl[(b,c,f,l)]	3.47 ± 0.17
	Acenaphthylene[(b,c,d,f)]	1.36 ± 0.04
	Fluorene[(b,c,d,f)]	1.27 ± 0.04
	Anthracene[(b,c,d,f,m)]	7.58 ± 0.35
	2-Methylanthracene[(b,c)]	5.88 ± 0.46
	Dibenzothiophene[(f,l,m)]	20.9 ± 1.5

[(a)] The non-certified values, unless otherwise footnoted, are weighted means of the mass fractions from multiple analytical methods [19]. The uncertainty listed is an expanded uncertainty about the mean [19,20], with coverage factor, *k* = 2, calculated by combining within-method variances with a between-method variance [21] following the ISO/JCGM Guide [22,23].
[(b)] GC/MS (IIa) on a proprietary relatively nonpolar phase after PFE at 200 °C and 13.8 MPa with toluene.
[(c)] GC/MS (IIb) on 50 % phenyl-substituted methylpolysiloxane phase same extract as GC/MS (IIa).
[(d)] GC/MS (IVa) on 50 % phenyl-substituted methylpolysiloxane phase after PFE at 200 °C and 13.8 MPa with DCM.
[(e)] GC/MS (VIII) on non-polar extra low bleed proprietary phase after PFE with toluene at 100 °C and two pressures (13.8 MPa and 20.7 MPa).
[(f)] GC/MS (VIII) on non-polar extra low bleed proprietary phase after PFE with toluene at 200 °C and two pressures (13.8 MPa and 20.7 MPa).
[(g)] The non-certified value is a weighted mean of average mass fractions, with one average from each of two or more analytical methods [19,20]. The expanded uncertainty is the half width of a symmetric 95 % parametric bootstrap confidence interval [24], which is consistent with the ISO/JCGM Guide [22,23]. The effective coverage factor, *k*, = 2.
[(h)] GC/MS (Ia) on a proprietary relatively nonpolar phase after PFE at 100 °C and 13.8 MPa with toluene.
[(i)] GC/MS (Va) on 50 % phenyl-substituted methylpolysiloxane phase after PFE at 100 °C with DCM.
[(j)] GC/MS (VIa) on 50 % phenyl-substituted methylpolysiloxane phase after PFE at 100 °C with DCM.
[(k)] GC/MS (IIIa) on 50 % phenyl-substituted methylpolysiloxane phase after PFE at 100 °C and 13.8 MPa with DCM.
[(l)] GC/MS (IVa) on 50 % phenyl-substituted methylpolysiloxane phase after PFE at 200 °C and 13.8 MPa with DCM.
[(m)] GC/MS (IVb) on dimethyl 50 % polysiloxane liquid crystalline stationary phase with same extract as GC/MS (IVa).
[(n)] GC/MS (VIb) on dimethyl 50 % polysiloxane liquid crystalline stationary phase with same extract as GC/MS (VIa).
[(o)] GC/MS (Vb) on dimethyl 50 % polysiloxane liquid crystalline stationary phase with same extract as GC/MS (Va).
[(p)] GC/MS (VII) on 50 % phenyl-substituted methylpolysiloxane phase after PFE at 100 °C with DCM.
[(q)] GC/MS (Ib) on 50 % phenyl-substituted methylpolysiloxane phase same extract as GC/MS (Ia).
[(r)] GC/MS (Environment Canada) on a proprietary relatively nonpolar phase.
[(s)] GC/MS (IIIb) on dimethyl 50 % polysiloxane liquid crystalline stationary phase with same extract as GC/MS (IIIa).
[(t)] Non-certified values based on GC/MS analyses on 5 % and/or 50 % phenyl-substituted methylpolysiloxane phase after PFE at 100 °C and 13.8 MPa with DCM from SRM 1650a.

Table 4. Non-Certified Mass Fraction Values for PAHs of Relative Molecular Mass 302 in SRM 1650b

PAH	Mass Fractions[(a,b)] (mg/kg)		
Dibenzo[*b,e*]fluoranthene	0.375	±	0.034
Naphtho[1,2-*b*]fluoranthene	2.31	±	0.21
Naphtho[1,2-*k*]fluoranthene and Naphtho[2,3-*j*]fluoranthene	1.71	±	0.17
Naphtho[2,3-*b*]fluoranthene	0.427	±	0.048
Dibenzo[*b,k*]fluoranthene	1.68	±	0.17
Dibenzo[*a,k*]fluoranthene	0.148	±	0.009
Dibenzo[*j,l*]fluoranthene	1.31	±	0.09
Dibenzo[*a,l*]pyrene	0.137	±	0.024
Naphtho[2,3-*e*]pyrene	0.770	±	0.073
Dibenzo[*a,e*]pyrene	1.13	±	0.049
Naphtho[2,1-*a*]pyrene	0.818	±	0.052
Dibenzo[*e,l*]pyrene	0.770	±	0.095
Benzo[*b*]perylene	0.125	±	0.013

(a) The non-certified values are weighted means of the mass fractions from multiple analytical methods [19]. The uncertainty listed with each value is an expanded uncertainty about the mean [19,20], with coverage factor, $k = 2$, calculated by combining within-method variances with a between-method variance [21] following the ISO/JCGM Guide [22,23].
(b) GC/MS on 50 % phenyl-substituted methylpolysiloxane phase after PFE at 100 °C and 13.8 MPa with DCM.

Table 5. Non-Certified Mass Fraction Values for Selected Nitro-PAHs in SRM 1650b
Based on Extraction Method and Conditions

Extraction Conditions	Mass Fractions[(a)] (µg/kg)		
PFE at temperatures between 100 °C and 200 °C			
1-Nitronaphthalene[(b,c,d,e)]	85.6	±	1.1
2-Nitronaphthalene[(b,c,d,e)]	236	±	5
2-Nitrobiphenyl[(b,c,d,e)]	16.1	±	0.7[(f)]
3-Nitrobiphenyl[(b,c,d,e)]	57.3	±	1.6
5-Nitroacenaphthene[(b,c,d,e)]	36.8	±	0.6[(f)]
2-Nitrofluorene[(b,c,d,e)]	46.0	±	1.5
PFE at 100 °C			
9-Nitroanthracene[(b,c,d,g)]	5940	±	90[(f)]
4-Nitrophenanthrene[(b,c)]	152	±	3
1-Nitrofluoranthene[(b,c)]	272	±	4[(f)]
8-Nitrofluoranthene[(b,c)]	112	±	6[(f)]
4-Nitropyrene[(b,c)]	137	±	4[(f)]
6-Nitrobenzo[*a*]pyrene[(b,c,g)]	1420	±	26[(f)]
3-Nitrobenzo[*e*]pyrene[(b,c)]	79.8	±	6.7[(f)]
1,3-Dinitropyrene[(b,c,g)]	45.5	±	1.6[(f)]
1,6-Dinitropyrene[(b,c)]	83.3	±	2.3[(f)]
PFE at 200 °C			
9-Nitroanthracene[(e)]	6930	±	210[(f)]

(a) The non-certified values, unless otherwise footnoted, are weighted means of the mass fractions from multiple analytical methods [19]. The listed uncertainty is an expanded uncertainty about the mean [19,20], with coverage factor, $k = 2$, calculated by combining within-method variances with a between-method variance [21] following the ISO/JCGM Guide [22,23].
(b) GC/NICI-MS (I) on 50 % phenyl-substituted methylpolysiloxane stationary phase.
(c) GC/NICI-MS (II) on 50 % phenyl-substituted methylpolysiloxane stationary phase.
(d) GC/NICI-MS (III) on 50 % phenyl-substituted methylpolysiloxane phase after PFE with toluene at 100 °C and two pressures (13.8 MPa and 20.7 MPa).
(e) GC/NICI-MS (III) on 50 % phenyl-substituted methylpolysiloxane phase after PFE with toluene at 200 °C and two pressures (13.8 MPa and 20.7 MPa).
(f) The non-certified value is a weighted mean of the average mass fractions, with one average from each of two or more analytical methods [19,20]. The expanded uncertainty is the half width of a symmetric 95 % parametric bootstrap confidence interval [24], which is consistent with the ISO/JCGM Guide [22,23] with an effective coverage factor, k, equals 2.
(g) GC/NCI-HRMS on 5 % phenyl-substituted methylpolysiloxane stationary phase.

Table 6. Non-Certified Value for Percent Extractable Mass for SRM 1650b[a]

	Mass Fractions[b] (%)
Percent Extractable Mass	20.2 ± 0.4

[a] Non-certified value for percent extractable mass reported for SRM 1650a is applicable to SRM 1650b.
[b] Non-certified value is the mean of results obtained using one analytical technique. The expanded uncertainty, U, is calculated as $U = ku_c$, where u_c is one standard deviation of the analyte mean, and the coverage factor, $k = 2$, is determined from the Student's t-distribution corresponding to the associated degrees of freedom and 95 % confidence level for each analyte.

Table 7. Non-Certified Values for Particle-Size Characteristics and Specific Surface Area for SRM 1650b[a]

Mean Particle Diameter, d(0.5)[b]	0.18	µm
Particle Diameter, d(0.1)[c]	0.12	µm
Particle Diameter, d(0.9)[d]	0.33	µm
Volume Weighted Mean[e]	0.22	µm
Specific Surface Area (S)[f]	108	m^2/g

[a] These values are provided for informational purposes only. The values have not been confirmed by an independent analytical technique as required for certification. See Figure 1 for particle-size distribution for SRM 1650b after 1 h and 24 h sonication.
[b] d(0.5) is the particle-size distribution parameter indicating the particle size below which 50 % of the volume is present.
[c] d(0.1) is the particle-size distribution parameter indicating the particle size below which 10 % of the volume is present.
[d] d(0.9) is the particle-size distribution parameter indicating the particle size below which 90 % of the volume is present.
[e] The volume weighted mean is the particle size in a uniform distribution.
[f] Specific surface area determined by multi-point N_2 gas adsorption BET equation [15].

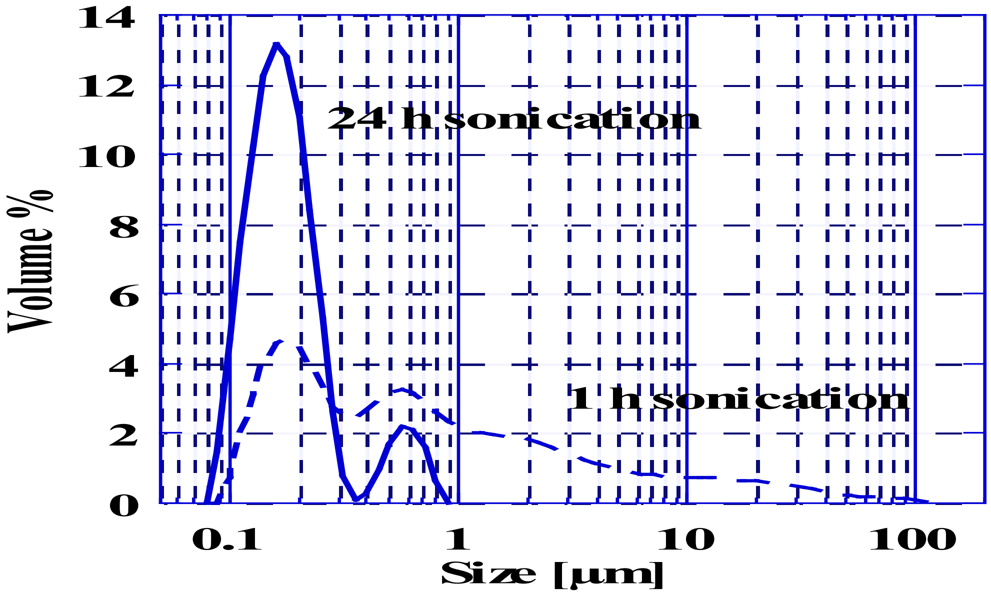

Figure 1. Particle-size distribution for SRM 1650b after 1 h and 24 h sonication.
See "Particle Size Information, Specific Surface Area, and Porosity" section for additional information.

REFERENCES

[1] SRM 1650; *Diesel Particulate Matter*; National Institute of Standards and Technology; U.S. Department of Commerce: Gaithersburg, MD (1985).

[2] SRM 1650a; *Diesel Particulate Matter*; National Institute of Standards and Technology; Department of Commerce: Gaithersburg, MD (2000).

[3] Poster, D.L.; Lopez de Alda, M.J.; Schantz, M.M.; Sander, L.C.; Vangel, M.G.; Wise, S.A.; *Development and Analysis of Three Diesel Particulate-Related Standard Reference Materials for the Determination of Chemical, Physical, and Biological Characteristics*; Polycyclic Aromat. Compd., Vol. 23, pp. 141–191 (2003).

[4] Poster, D.L.; Benner, B.A., Jr.; Schantz, M.M.; Sander, L.C.; Vangel, M.G.; Wise, S.A.; *Determination of Methyl-Substituted Polycyclic Aromatic Hydrocarbons in Diesel Particulate-Related Standard Reference Materials*; Polycyclic Aromat. Compd., Vol. 23, pp. 113–139 (2003).

[5] Beauchamp, C.R.; Camara, J.E.; Carney, J.; Choquette, S.J.; Cole, K.D.; DeRose, P.C.; Duewer, D.L.; Epstein, M.S.; Kline, M.C.; Lippa, K.A.; Lucon, E.; Phinney, K.W.; Polakoski, M.; Possolo, A.; Sharpless, K.E.; Sieber, J.R.; Toman, B.; Winchester, M.R.; Windover, D.; *Metrological Tools for the Reference Materials and Reference Instruments of the NIST Material Measurement Laboratory*; NIST Special Publication (NIST SP) 260-136, 2020 Edition; U.S. Government Printing Office: Washington, DC (2020); available at https://nvlpubs.nist.gov/nistpubs/SpecialPublications/NIST.SP.260-136-2020.pdf (accessed July 2021).

[6] Lippa, K.A.; Schantz, M.M.; *Microhomogeneity Evaluation of Polycyclic Aromatic Hydrocarbons in Particulate Standard Reference Materials*; Anal. Bioanal. Chem., Vol. 387, pp. 2389–2399 (2007).

[7] Schantz, M.M.; Nichols, J.J.; Wise, S.A.; *Evaluation of Pressurized Fluid Extraction for the Extraction of Environmental Matrix Reference Materials*; Anal. Chem., Vol. 69, pp. 4210–4219 (1997).

[8] Schubert, P.; Schantz, M.M.; Sander, L.C.; Wise, S.A.; *Determination of Polycyclic Aromatic Hydrocarbons with Molecular Mass 300 and 302 in Environmental-Matrix Standard Reference Materials by Gas Chromatography-Mass Spectrometry*; Anal. Chem., Vol. 75, pp. 234–246 (2003).

[9] Bezabeh, D.Z.; Bamford, H.A.; Schantz, M.M.; Wise, S.A.; *Determination of Nitrated Polycyclic Aromatic Hydrocarbons in Diesel Particulate-Related Standard Reference Materials by Using Gas Chromatography/Mass Spectrometry With Negative Ion Chemical Ionization*; Anal. Bioanal. Chem., Vol. 375, pp. 381–388 (2003).

[10] Bamford, H.A.; Bezabeh, D.Z.; Schantz, M.M.; Wise, S.A.; Baker, J.E.; *Determination and Comparison of Nitrated-Polycyclic Aromatic Hydrocarbons Measured in Air and Diesel Particulate Reference Materials*; Chemosphere, Vol. 50, pp. 575–587 (2003).

[11] Schantz, M.M.; McGaw, E.; Wise, S.A.; *Pressurized Liquid Extraction of Diesel and Air Particulate Standard Reference Materials: Effects of Extraction Temperature and Pressure*; Anal. Chem., Vol. 84, pp. 8222–8231 (2012).

[12] Bergvall, C.; Westerholm, R.; *Determination of 252–302 Da and Tentative Identification of 316–376 Da Polycyclic Aromatic Hydrocarbons in Standard Reference Materials 1649a Urban Dust and 1650b and 2975 Diesel Particulate Matter by Accelerated Solvent Extration — HPLC-GC-MS*; Anal. Bioanal. Chem., Vol. 391, pp. 2235–2248 (2008).

[13] Masala, S.; Ahmed, T.; Bergvall, C.; Westerholm, R.; *Improved Efficiency of Extraction of Polycyclic Aromatic Hydrocarbons (PAHs) from the National Institute of Standards and Technology (NIST) Standard Reference Material Diesel Particulate Matter (SRM 2975) using Accelerated Solvent Extraction*; Anal. Bioanal. Chem., Vol. 401, pp. 3305–3315 (2011).

[14] Gregg, S.J.; Sing, K.S.W.; *Adsorption, Surface Area and Porosity*; 2nd, Academic Press, London (1982).

[15] Brunauer, S.; Emmett, P.; Teller, E.; *Adsorption of Gases in Multimolecular Layers*; J. Am. Chem. Soc., Vol. 60, pp. 309–319 (1938).

[16] Barrett, E.P.; Joyner, L.G.; Halenda, P.P.; *The Determination of Pore Volume and Area Distributions in Porous Substances. I. Computations for Nitrogen Isotherms*; J. Am. Chem. Soc., Vol. 73, pp. 373–380 (1951).

[17] Lewtas, J.; Claxton, L.D.; Rosenkranz, H.S.; Schuetzle, D.; Shelby, M.; Matsushita, H.; Wurgler, F.E.; Zimmermann, F.K.; Lofroth, G.; May, W.E.; Krewski, D.; Matsushima, T.; Ohnishi, Y.; Gopalan, H.N.G.; Sarin, R.; Becking, G.C.; *Design and Implementation of a Collaborative Study of the Mutagenicity of Complex Mixtures in Salmonella typhimurium*; Mutat. Res., Vol. 276, pp. 3–9 (1992).

[18] Claxton, L.D.; Douglas, G.; Krewski, D.; Lewtas, J.; Matsushita, H.; Rosenkranz, H.; *Overview, Conclusions, and Recommendations of the IPCS Collaborative Study on Complex Mixtures*; Mutat. Res., Vol. 276, pp. 61–80 (1992).

[19] DerSimonian, R.; Laird, N.; *Meta-Analysis in Clinical Trials*; Control Clin. Trials, Vol. 7, pp. 177–188 (1986).

[20] Rukhin, A.L.; *Weighted Means Statistics in Interlaboratory Studies*; Metrologia, Vol. 46, pp. 323–331 (2009).

[21] Horn, R.D.; Horn, R.A.; Duncan, D.B.; *Estimating Heteroscedastic Variances in Linear Models*; J. Am. Stat. Assoc., Vol. 70, pp. 380–385 (1975).

[22] JCGM 100:2008; *Evaluation of Measurement Data — Guide to the Expression of Uncertainty in Measurement* (GUM 1995 with Minor Corrections); Joint Committee for Guides in Metrology (JCGM) (2008); available at

https://www.bipm.org/en/publications/guides (accessed July 2021); see also Taylor, B.N.; Kuyatt, C.E.; *Guidelines for Evaluating and Expressing the Uncertainty of NIST Measurement Results*; NIST Technical Note 1297, U.S. Government Printing Office: Washington, DC (1994); available at https://www.nist.gov/pml/nist-technical-note-1297 (accessed July 2021).
[23] JCGM 101:2008; *Evaluation of Measurement Data — Supplement 1 to the "Guide to the Expression of Uncertainty in Measurement" — Propagation of Distributions using a Monte Carlo Method*; JCGM (2008); available at https://www.bipm.org/en/publications/guides (accessed July 2021).
[24] Efron, B.; Tibshirani, R.J.; *An Introduction to the Bootstrap*; Chapman & Hall: London, UK (1993).
[25] MacCrehan, W.A.; May, W.E.; Yang, S.D.; Benner, B.A., Jr.; *Determination of Nitro Polynuclear Aromatic Hydrocarbons in Air and Diesel Particulate Matter Using Liquid Chromatography with Electrochemical and Fluorescence Detection*; Anal. Chem., Vol. 60, pp. 194–199 (1988).

Certificate Revision History: 07 July 2021 (Change of expiration date; change of nitro-PAHs certified values to non-certified values due to incomplete stability testing from lack of appropriate calibrants; all other measurands previously labeled as reference or information values were converted to non-certified values; editorial changes); 17 July 2013 (Update certified and reference values for PAHs and nitro-PAHs to address the effect of temperature used for extraction; extension of certification period; editorial changes); 27 September 2006 (Original certificate date).

Users of this SRM should ensure that the Certificate of Analysis in their possession is current. This can be accomplished by contacting the SRM Program: telephone (301) 975-2200; e-mail srminfo@nist.gov; or via the Internet at https://www.nist.gov/srm.
