# Supplementary material for: Co-exposure to urban particulate matter and aircraft noise adversely impacts the cerebro-pulmonary-cardiovascular axis in mice
Source: Redox Biol. 2022 Dec 18;59:102580. doi: 10.1016/j.redox.2022.102580 (PMC9804249; doi:10.1016/j.redox.2022.102580)
Supplement: Multimedia component 2 [file mmc2.pdf]

National Institute of Standards & Technology

Certificate of Analysis

Standard Reference Material® 1648a

Urban Particulate Matter

This Standard Reference Material (SRM) is atmospheric particulate matter collected in an urban area and is intended primarily for use as a quality control material and in the evaluation of methods used in inorganic analysis and for the determination of selected polycyclic aromatic hydrocarbons (PAHs), nitro-substituted PAHs (nitro-PAHs), polychlorinated biphenyl (PCB) congeners, and chlorinated pesticides in atmospheric particulate material and similar matrices. All constituents for which certified, reference, and information values are provided in SRM 1648a were naturally present in the material before processing. While not intended to be representative of the area where it was collected, nor of a contemporary composition of an urban aerosol, its use should typify the analytical problems encountered when analyzing atmospheric particulate samples obtained from industrialized urban areas. A unit of SRM 1648a consists of a bottle containing 2 g of atmospheric particulate matter.

Certified Mass Fraction Values: Certified values for elements are provided in Table 1, and values for the content of selected PAHs and PCB congeners are provided in Tables 2 and 3, respectively. The certified values are based on the agreement of results from two or more chemically independent analytical techniques obtained at NIST and collaborating expert laboratories [1]. A NIST certified value is a value for which NIST has the highest confidence in its accuracy in that all known or suspected sources of bias have been investigated or taken into account.

Reference Mass Fraction Values: Reference values are provided for the content of additional elements in Table 4 and for additional PAHs, nitro-PAHs, additional PCB congeners, and chlorinated pesticides in Tables 5 to 8, respectively. In Tables 5a and 5b, the reference values for some PAHs are listed more than once depending on the extraction conditions used (see "Preparation and Analysis"). Reference values are non-certified values that are the best estimate of the true values. The values do not meet the NIST criteria for certification and are provided with associated uncertainties that may reflect only measurement precision, may not include all sources of uncertainty, or may reflect a lack of sufficient statistical agreement among multiple analytical methods [1].

Information Values: Information mass fraction values are provided in Table 9 for additional elements. Information values for particle-size characteristics are provided in Table 10. An information value is considered to be a value that will be of interest and use to the SRM user, but insufficient information is available to adequately assess the uncertainty associated with the value, or it is a value derived from a limited number of analyses [1]. Information values cannot be used to establish metrological traceability.

Expiration of Certification: The certification of **SRM 1648a** is valid, within the measurement uncertainty specified, until **01 October 2027**, provided the SRM is handled and stored in accordance with the instructions given in this certificate (see "Instructions for Handling, Storage, and Use"). The certification is nullified if the SRM is damaged, contaminated, or otherwise modified.

Maintenance of SRM Certification: NIST will monitor this SRM over the period of its certification. If substantive technical changes occur that affect the certification before the expiration of this certificate, NIST will notify the purchaser. Registration (see attached sheet or register online) will facilitate notification.

Coordination of the investigations and technical measurements leading to the certification of SRM 1648a was under the leadership of R. Zeisler, M.M. Schantz, and S.E. Long formerly of NIST.

Carlos A. Gonzalez, Chief
Chemical Sciences Division

Steven J. Choquette, Director
Office of Reference Materials

Gaithersburg, MD 20899
Certificate Issue Date: 06 October 2020
Certificate Revision History on Last Page

Collaborating laboratories and analysts include: Desert Research Institute, Reno, NV: S.D. Kohl; Federal Institute for Materials Research and Testing (BAM), Germany: M. Hedrich and C. Segebade; National Institute of Nuclear Research (ININ) and National University of Mexico, Mexico: M. Navarrete, G. Zarazúa, P. Avila, T. Martínez, and C. Solís; Sunset Laboratory Inc., Tigard, OR: R. Cary; University of São Paulo, Brazil: C.S. Nomura and P.V. Oliveira.

Statistical consultation was provided by N.A. Heckert and S.D. Leigh of the NIST Statistical Engineering Division.

Support aspects involved in the issuance of this SRM were coordinated through the NIST Office of Reference Materials.

INSTRUCTIONS FOR HANDLING, STORAGE, AND USE

Handling: This readily aerosolized material is a naturally occurring particulate material and may contain constituents of known and unknown toxicities and mutagenicities. A significant portion of the material is in the range of inhalable airborne particulate matter based on its particle size. For health and safety information, see the Safety Data Sheet (SDS).

Storage: Store SRM 1648a in its original amber glass bottle at temperatures less than 30 °C, away from sources of radiation, including ultraviolet lamps or sunlight.

Use: Prior to removal of sub-samples for analysis, the contents of the bottle should be mixed thoroughly. The recommended minimum dry sample size is: 5 mg for determination of the certified inorganic constituents (except Hg which requires a 160 mg sample size); and 150 mg sample size for PAHs constituents. These respective sample sizes provide less than 1 % variability contribution from inhomogeneity components. The mass fractions of constituents in SRM 1648a are reported on a dry-mass basis. The SRM "as received" contains a mass fraction of approximately 2.3 % moisture. The aerosol sample should be dried to a constant mass before weighing for analysis or a separate sub-sample of the SRM should be removed from the bottle at the time of analysis and dried to determine mass fractions on a dry-mass basis. If the constituents of interest are volatile, then the moisture must be determined with a separate sub-sample. The recommended drying procedures are discussed in "*Conversion to Dry-Mass Basis*." Equivalent procedures may be used, but the temperature of 30 °C should not be exceeded.

PREPARATION AND ANALYSIS(1)

Sample Collection and Preparation: The SRM was prepared from urban particulate matter collected in the St. Louis, MO area in a baghouse specially designed for this purpose. The material was collected over a period greater than 12 months during 1976 and 1977; therefore, it is a time-integrated sample from that period. The material was removed from the filter bags, combined into a single lot, screened through a fine mesh sieve to remove extraneous materials, and thoroughly blended in a V-blender. The lot was divided; one portion was used for the previous issue of this SRM (SRM 1648), and the other 8 kg portion of the material was set aside and is used for this renewal, SRM 1648a. The material has been re-blended, bottled, and reanalyzed to provide updated certified values, reference values, and information values for inorganic and organic analyses. Thus, SRM 1648a consists of the same particulate material issued in 1978 as SRM 1648.

Conversion to Dry-Mass Basis: The results for the constituents in SRM 1648a are reported on a dry-mass basis; however, the material "as received" contains residual moisture. Drying in a desiccator over fresh $Mg(ClO_4)_2$ for 48 h is recommended. Alternately, drying in a desiccator over $CaSO_4$ for seven days results in similar moisture loss. Drying with the use of heat is not recommended.

Particle Size Distribution: The particle size distribution of SRM 1648a was obtained using a commercial laser diffraction instrument manufactured by Malvern Instruments (Worchestershire, UK). The particulate matter was measured in aqueous suspension and is detailed in "*Supplemental Information for SRM 1648a*."

(1) Certain commercial equipment, instruments, or materials are identified in this certificate to adequately specify the experimental procedure. Such identification does not imply recommendation or endorsement by the National Institute of Standards and Technology, nor does it imply that the materials or equipment identified are necessarily the best available for the purpose.

INORGANIC CONSTITUENTS

Analytical Approach: In the investigations at NIST, instrumental neutron activation analysis (INAA) and neutron capture prompt gamma activation analysis (PGAA) were used to directly determine mass fraction values for the elements, except for mercury. Wavelength-dispersive X-ray fluorescence spectrometry (WDXRF) was used to determine specific count rates for selected elements in SRM 1648a. Additional measurements with photon activation analysis (PAA), proton-induced X-ray emission spectrometry (PIXE), solid-sample graphite furnace atomic absorption spectrometry (SS-GFAAS) and WDXRF were provided by collaborating laboratories. All assays were designed to establish comparability of values between those conventionally certified in SRM 1648, representing the parent material, and the SRM 1648a measured in small sample sizes. These measurements confirmed that the composition of the material had not changed in storage and that the measured values in SRM 1648 can be utilized for confirmation of the value assignment of SRM 1648a.

Mercury measurements were made using cold-vapor mercury generation coupled with inductively coupled-mass spectrometry (ICP-MS) isotope ratio measurements. Single subsamples (160 mg to 230 mg) were taken from each of eight bottles and spiked with ^{201}Hg followed by microwave digestion. The value assignment of the mass fraction of mercury is based on cold-vapor (ICP-MS).

Homogeneity Assessment: The homogeneity of SRM 1648a was assessed by analyzing samples of approximately 1 mg from bottles selected by stratified random sampling (as described in "Homogeneity Assessment for Inorganic Constituents"). Kurfürst homogeneity factors derived from the results confirmed that a smaller than 1 % relative heterogeneity component of the uncertainty in the results is encountered for most elements by selecting sample sizes of 5 mg or larger.

ORGANIC CONSTITUENTS

Polycyclic Aromatic Hydrocarbons: The PAHs in SRM 1648a were determined by Soxhlet extraction or pressurized-fluid extraction (PFE) followed by analysis of the extracts using gas chromatography/mass spectrometry (GC/MS) on columns of differing selectivity. Multiple sets of GC/MS results, designated as GC/MS (Ia), GC/MS (Ib), GC/MS (Ic), GC/MS (IIa), GC/MS (IIb), and GC/MS (III) were used in the certification process.

GC/MS (I): For GC/MS (I) analyses, duplicate subsamples of between 150 mg and 250 mg from 10 bottles were extracted using a Soxhlet extraction for 20 h with dichloromethane. The concentrated extract was passed through an aminopropylsilane solid-phase extraction (SPE) cartridge and eluted with 20 % (volume fraction) dichloromethane in hexane. The PAH fraction was then analyzed by GC/MS using three columns with differing selectivity: (a) a 0.25 mm i.d. × 60 m fused silica capillary column with a 50 % (mole fraction) phenyl-substituted methylpolysiloxane phase (0.25 µm film thickness; DB-17ms, Agilent Technologies, Wilmington, DE), designated as GC/MS (Ia); (b) a 0.25 mm i.d. × 60 m fused silica capillary column with a relatively nonpolar phase (0.25 µm film thickness; DB-XLB, Agilent Technologies), designated as GC/MS (Ib); and (c) a 0.25 mm i.d. × 20 m dimethyl 50 % liquid crystal polysiloxane phase (0.25 µm film thickness; LC-50, J&K Environmental, Milton, Ontario, Canada), designated as GC/MS (Ic).

GC/MS (II): The GC/MS (II) analyses were conducted on test portions of 180 mg to 300 mg from each of three bottles extracted using PFE with dichloromethane at 100 °C and 13.8 MPa (2000 psi), designated as GC/MS (IIa) and test portions of 180 mg to 300 mg from three bottles extracted using PFE with dichloromethane at 200 °C and 13.8 MPa, designated as GC/MS (IIb). The concentrated extracts were passed through a silica SPE cartridge and eluted with10 % (volume fraction) dichloromethane in hexane. The PAH fraction was then analyzed by GC/MS using a 0.25 mm i.d. × 60 m fused silica capillary column with a proprietary relatively nonpolar phase (0.25 µm film thickness; DB-XLB).

GC/MS (III): For GC/MS (III), test portions of approximately 500 mg from each of six bottles were extracted using PFE with dichloromethane at 100 °C and 13.8 MPa. The samples were cleaned-up using automated SPE with 1.8 g alumina columns and eluting with 9 mL of 35 % (volume fraction) dichloromethane in hexane. The samples were analyzed by GC/MS with a 0.25 mm i.d. × 60 m fused silica capillary column containing a 50 % (mole fraction) phenyl-substituted methylpolysiloxane phase (0.25 µm film thickness; DB-17, Agilent Technologies).

For all of the GC/MS measurements described above, selected perdeuterated PAHs were added to the particulate matter prior to solvent extraction for use as internal standards for quantification purposes.

Homogeneity Assessment for PAHs: The homogeneity of SRM 1648a was assessed by analyzing duplicate test portions of 150 mg to 250 mg from 10 randomly selected bottles. Samples were extracted, processed, and analyzed as described above for the GC/MS (I). No statistically significant differences between bottles were observed for the PAH mass fractions at a 150 mg to 250 mg sample size.

Value Assignment for PAHs: Certified mass fraction values were assigned for PAHs in SRM 1648a (Table 2) based on analyses using Soxhlet extraction and PFE at both 100 °C and 200 °C when the values did not change based on the extraction method [2]. For some PAHs, PFE at higher temperatures resulted in higher levels of extraction for air particulate SRMs [3,4]. For these PAHs, the different mass fractions are reported as a reference values in Tables 5a and 5b and should be considered "method dependent".

Nitro-Substituted PAHs: SRM 1648a was analyzed at NIST for the determination of nitro-substituted PAHs. Duplicate test portions of 150 mg to 250 mg from 10 bottles were extracted using Soxhlet extraction for 20 h with dichloromethane. The concentrated extract was passed through an aminopropylsilane SPE cartridge and eluted with 20 % (volume fraction) dichloromethane in hexane. The concentrated eluant was then subjected to normal-phase liquid chromatography (LC) using a semi-preparative amino/cyano phase column with a mobile phase of 20 % (volume fraction) dichloromethane in hexane to isolate the nitro-PAH fraction. The nitro-PAH fraction was analyzed by GC with negative ion chemical ionization mass spectrometry (GC/NICI-MS) using a 0.25 mm i.d. × 60 m fused silica capillary column containing a 50 % phenyl-substituted methylpolysiloxane stationary phase (0.25 µm film thickness). Selected perdeuterated nitro-PAHs were added to the air particulate matter prior to extraction for use as internal standards for quantification purposes.

Value Assignment for Nitro PAHs: Nitro-PAHs were determined in SRM 1648a using only Soxhlet extraction, and as only a single extraction method was used, the values are provided as reference values (Table 6). The reference values should also be considered as "method dependent" values because they are dependent on the extraction method and temperature.

PCBs and Chlorinated Pesticides: SRM 1648a was analyzed for selected PCB congeners and chlorinated pesticides using GC/MS. Four sets of GC/MS results, designated as GC/MS (Va), GC/MS (Vb), GC/MS (VIa), and GC/MS (VIb), were used in the certification process.

GC/MS (V): For GC/MS (V) analyses, a single test portion of 150 mg to 250 mg from each of 6 bottles was Soxhlet extracted for 20 h with dichloromethane. The concentrated extract was passed through an aminopropylsilane SPE cartridge and eluted with 20 % (volume fraction) dichloromethane in hexane. The chlorinated compounds were then analyzed by GC/MS in the electron ionization mode (EI, noted as GC/MS throughout this Certificate of Analysis) and in the NICI mode (GC/NICI-MS). For the GC/MS analysis, designated as GC/MS (Va), a 0.25 mm i.d. × 60 m fused silica capillary column with a 50 % (mole fraction) phenyl-substituted methylpolysiloxane phase (0.25 µm film thickness) was used while for the GC/NICI-MS, designated as GC/MS (Vb), a 0.25 mm i.d. × 60 m fused silica capillary column with a relatively nonpolar phase (0.25 µm film thickness; DB-XLB) was used.

GC/MS (VI): For GC/MS (VI), test portions from six bottles of approximately 150 mg to 250 mg were extracted using PFE with dichloromethane at 100 °C and 13.8 MPa. The samples were cleaned up using automated SPE with alumina cartridges and then with acidified silica cartridges. The first fraction from the silica cartridges was concentrated and then further cleaned-up using size-exclusion chromatography. The samples were analyzed by GC/MS with a 0.18 mm i.d. × 30 m fused silica capillary column containing a 5 % phenyl-substituted methylpolysiloxane phase (0.18 µm film thickness), designated as GC/MS (VIa) and by GC/NICI-MS with a 0.18 mm i.d. × 30 m fused silica capillary column with a relatively nonpolar phase (0.18 µm film thickness; DB-XLB), designated as GC/MS (VIb).

For the GC/MS and GC/NICI-MS analyses, two PCB congeners that are not significantly present in the air particulate extract (PCB 103 and PCB 198 [5,6]) and selected ^{13}C-labeled PCB congeners and pesticides were added to the air particulate material prior to extraction for use as internal standards for quantification purposes.

Certified Mass Fraction Values of Elements: The certified mass fraction value for each element in Table 1, expressed on a dry-mass basis, is an equally weighted mean of the individual sets of results provided by the individual NIST methods and individual methods of the collaborating laboratories, where used. The NIST results and some of those provided by collaborating laboratories included estimates of all recognized sources of uncertainty. Some collaborating laboratory results were provided without complete uncertainty budgets. These uncertainties were augmented using an approach that accounts for the differences among the results obtained by different methods [7]. For values solely determined by NIST, the certified mass fraction are weighted means of the mass fractions from multiple analytical methods [8]. The uncertainty listed with each value is an expanded uncertainty about the mean [8,9], with coverage factor, $k = 2$, with an approximately 95 % confidence interval calculated according to the method described in the ISO/JCGM Guide [10,11]. The measurands are the total mass fraction for each element in urban particulate matter as listed in Table 1. Metrological traceability is to the SI derived units of mass fraction (expressed as mg/kg or percent on a dry-mass basis).

Table 1. Certified Mass Fraction Values (Dry-Mass Basis) of Elements

Element	Mass Fraction			Units
Aluminum (Al)[a,b]	3.43	±	0.13	%
Antimony (Sb)[a,c]	45.4	±	1.4	mg/kg
Arsenic (As)[a,c,d,e]	115.5	±	3.9	mg/kg
Bromine (Br)[a,b,e]	502	±	10	mg/kg
Calcium (Ca)[a,b,c,e]	5.84	±	0.19	%
Cadmium (Cd)[d,f]	73.7	±	2.3	mg/kg
Cerium (Ce)[a,c]	54.6	±	2.2	mg/kg
Chlorine (Cl)[a,b]	4543	±	47	mg/kg
Cobalt (Co)[a,c]	17.93	±	0.68	mg/kg
Chromium (Cr)[a,b]	402	±	13	mg/kg
Copper (Cu)[a,d,e]	610	±	70	mg/kg
Iron (Fe)[a,b,e,f]	3.92	±	0.21	%
Lead (Pb)[b,c,d,e]	0.655	±	0.033	%
Magnesium (Mg)[a,b]	0.813	±	0.012	%
Manganese (Mn)[a,b,e]	790	±	44	mg/kg
Mercury (Hg)[g]	1.323	±	0.064	mg/kg
Nickel (Ni)[b,c,d,e]	81.1	±	6.8	mg/kg
Potassium (K)[a,b,e,f]	1.056	±	0.049	%
Rubidium (Rb)[a,c]	51.0	±	1.5	mg/kg
Sodium (Na)[a,b]	4240	±	60	mg/kg
Strontium (Sr)[b,e]	215	±	17	mg/kg
Sulfur (S)[b,f]	5.51	±	0.36	%
Titanium (Ti)[a,b,c,e,f]	4021	±	86	mg/kg
Vanadium (V)[a,e]	127	±	11	mg/kg
Zinc (Zn)[a,b,c,d,e]	4800	±	270	mg/kg

[a] INAA (NIST)
[b] WDXRF (NIST)
[c] PAA
[d] SS-GFAAS
[e] PIXE
[f] PGAA (NIST)
[g] Isotope dilution ICP-MS (NIST)

Certified Mass Fraction Values for PAHs: The certified mass fraction values in Table 2 for the PAHs are weighted means of the mass fractions from multiple analytical methods [8]. The uncertainty listed with each value is an expanded uncertainty, U, calculated as $U = ku_c$, with coverage factor, $k = 2$. The expanded uncertainty about the mean is determined by combining within method variances with a between method variance [12] following the ISO/JCGM Guide [10,11]. The measurands are the total mass fraction of each PAHs in urban particulate matter as listed in Table 2. Metrological traceability is to the SI derived units of mass fraction (expressed as mg/kg on a dry-mass basis).

Table 2. Certified Mass Fraction Values (Dry-Mass Basis) for PAHs in SRM 1648a

	Mass Fractions (mg/kg)		
Phenanthrene(a,b,c,d,e)	4.86	±	0.17
2-Methylphenathrene(a,b,d)	0.96	±	0.12
3-Methylphenanthrene(a,b,d)	0.614	±	0.067
Fluoranthene(a,b,c,d,e)	8.07	±	0.14
Pyrene(a,b,c,d,e)	5.88	±	0.07
Benzo[*ghi*]fluoranthene(a,b,c,d,e)	1.17	±	0.05
Benz[*a*]anthracene(a,b,c,d,e)	2.71	±	0.15
Chrysene(b,c,d)	6.12	±	0.06
Triphenylene(b,c,d)	2.04	±	0.13
Benzo[*k*]fluoranthene(a,b,c,d,e)	3.03	±	0.24
Benzo[*e*]pyrene(a,b,c,d,e)	4.85	±	0.07
Benzo[*a*]pyrene(a,b,c,d,e)	2.57	±	0.10
Perylene(a,b,c,d,e)	0.742	±	0.048
Benzo[*ghi*]perylene(a,b,c,d,e)	5.00	±	0.18
Indeno[1,2,3-*cd*]pyrene(a,b,c,d,e)	4.17	±	0.17
Dibenz[*a,j*]anthracene(a,b,c,d,e)	0.407	±	0.039
Benzo[b]chyrsene(a,b,c,d,e)	0.405	±	0.041
Picene(a,b,c,d,e)	0.586	±	0.058
Coronene(a,b,c,d,e)	2.28	±	0.10
Dibenzo[*b,k*]fluoranthene(a,b,c,d,e)	0.947	±	0.054
Dibenzo[*a,e*]pyrene(a,b,c,d,e)	0.622	±	0.045

(a) GC/MS (Ia) on a 50 % phenyl-substituted methylpolysiloxane phase after Soxhlet extraction with dichloromethane.
(b) GC/MS (Ib) on a relatively non-polar phase using same extracts as GC/MS (Ia).
(c) GC/MS (Ic) on a dimethyl 50 % liquid crystal polysiloxane phase using same extracts as GC/MS (Ia).
(d) GC/MS (IIa and IIb) on a relatively non-polar phase after PFE at 100 °C and at 200 °C with dichloromethane at 13.8 MPa.
(e) GC/MS (III) on a 50 % phenyl-substituted methylpolysiloxane phase after PFE at 100 °C with dichloromethane at 13.8 MPa.

Certified Mass Fraction Values for PCB Congeners: The certified mass fraction values for the PCB Congeners in Table 3 are weighted means of the mass fractions from multiple analytical methods [8]. The uncertainty listed with each value is an expanded uncertainty, U, calculated as $U = ku_c$, with coverage factor, $k = 2$. The expanded uncertainty about the mean is determined by combining within method variances with a between method variance [12] following the ISO/JCGM Guide [10,11]. The measurands are the total mass fraction for each PCB Congeners in urban particulate matter as listed in Table 3. Metrological traceability is to the SI derived units of mass fraction (expressed as µg/kg on a dry-mass basis).

Table 3. Certified Mass Fraction Values (Dry-Mass Basis) for PCB Congeners[(a)] in SRM 1648a

			Mass Fractions (µg/kg)		
PCB	105	(2,3,3',4,4'-Pentachlorobiphenyl)[(b,c,d)]	19.6	±	2.3
PCB	110	(2,3,3',4',6-Pentachlorobiphenyl)[(b,c)]	25.4	±	1.9
PCB	149	(2,2',3,4',5',6-Hexachlorobiphenyl)[(b,d)]	38.9	±	2.6
PCB	153	(2,2',4,4',5,5'-Hexachlorobiphenyl)[(b,d)]	40.0	±	4.9
PCB	183	(2,2',3,4,4',5',6-Heptachlorobiphenyl)[(c,d)]	7.99	±	0.54
PCB	187	(2,2',3,4',5,5',6-Heptachlorobiphenyl)[(b,c,d)]	17.1	±	1.4
PCB	194	(2,2',3,3',4,4',5,5'-Octachlorobiphenyl)[(c,d)]	19.1	±	2.2

[(a)] PCB congeners are numbered according to the scheme proposed by Ballschmiter and Zell [5] and later revised by Schulte and Malisch [6] to conform with IUPAC rules; for the specific congeners identified in this SRM, the Ballschmiter-Zell numbers correspond to those of Schulte and Malisch. When two or more congeners are known to coelute under the conditions used, the PCB congener listed first is the major component and the additional congeners may be present as minor components. The quantitative results are based on the response of the congener listed first.
[(b)] GC/MS (Va) on a 50 % phenyl-substituted methylpolysiloxane phase after Soxhlet extraction with dichloromethane.
[(c)] GC/MS (VIa) on a 5 % phenyl-substituted methylpolysiloxane phase after PFE with dichloromethane.
[(d)] GC/MS (Vb) on a relatively non-polar phase using same extracts as GC/MS (Va).

Reference Mass Fraction Values for Elements: Reference values are based on NIST results from one method for each reported element in Table 4. The results are validated by the values previously reported for SRM 1648 [13] since INAA and XRF procedures established equivalence of SRM 1648a with SRM 1648 based on previously certified values in the latter. The uncertainties of the NIST results were augmented [7] on the basis of the previously reported differences among the results obtained by different methods in SRM 1648 [13]. These results do not fulfill the criteria for certification since a full estimate of method bias for the determinations in SRM 1648a is not available. The reporting follows the ISO/JCGM Guide [10,11]. The measurands are the total mass fraction of elements in urban particulate matter as determined by the method listed in Table 4. Metrological traceability is to the SI derived units of mass fraction (expressed as mg/kg or percent on a dry-mass basis).

Table 4. Reference Mass Fraction Values (Dry Mass Basis) for Elements

Element	Mass Fraction			Units
Boron (B)[(a)]	161	±	9	mg/kg
Cesium (Cs)[(b)]	3.4	±	0.2	mg/kg
Lanthanum (La)[(b)]	39	±	3	mg/kg
Samarium (Sm)[(b)]	4.3	±	0.3	mg/kg
Selenium (Se)[(b)]	28.4	±	1.1	mg/kg
Silicon (Si)[(c)]	12.8	±	0.4	%
Silver (Ag)[(b)]	6.0	±	0.3	mg/kg
Tungsten (W)[(b)]	4.6	±	0.3	mg/kg

[(a)] PGAA (NIST)
[(b)] INAA (NIST)
[(c)] WDXRF (NIST)

Reference Mass Fraction Values for PAHs: The measurands are the total mass fraction for each PAH in urban particulate matter listed in Table 5a and 5b based on the extraction method and conditions. Metrological traceability is to the SI derived units of mass fraction (expressed as mg/kg on a dry-mass basis).

Table 5a. Reference Mass Fraction Values (Dry-Mass Basis) for PAHs in SRM 1648a
Based on Extraction Method and Conditions

Extraction Conditions	Mass Fractions[(a)] (mg/kg)		
Soxhlet extraction or PFE at 100 °C			
Naphthalene[(b,c,d)]	1.23	±	0.58[(e)]
Biphenyl[(c,d)]	0.186	±	0.031
Acenaphthene[(c,d)]	0.250	±	0.083
Acenaphthylene[(c,d)]	0.173	±	0.012
Fluorene[(b,c,d,g)]	0.251	±	0.035
Dibenzothiophene[(b,c,d,g)]	0.262	±	0.015
Anthracene[(b,c,d,f,g)]	0.459	±	0.013
1-Methylphenanthrene[(b,c,g)]	0.438	±	0.080[(e)]
4H-Cyclopenta[*def*]phenanthrene[(b,c,d,f,g)]	0.291	±	0.021
8-Methylfluoranthene[(b,g)]	0.275	±	0.022
2-Methylpyrene[(b,g)]	0.330	±	0.018
4-Methylpyrene[(b,c,g)]	0.321	±	0.021
Retene[(b,c,g)]	0.687	±	0.052
Cyclopenta[*cd*]pyrene[(b,d,f,g)]	0.239	±	0.045[(e)]
Benzo[*c*]phenanthrene[(b,c,d,f,g)]	0.819	±	0.034
2-Methylchrysene[(b,g)]	0.538	±	0.032
3-Methylchrysene[(b,g)]	0.351	±	0.037
6-Methylchrysene[(b,g)]	0.204	±	0.020
Benzo[*b*]fluoranthene[(b,d,f)]	8.89	±	0.05[(e)]
Benzo[*j*]fluoranthene[(b,d,f)]	3.20	±	0.03[(e)]
Benzo[*a*]fluoranthene[(b,c,d,f,g)]	0.449	±	0.045[(e)]
Anthanthrene[(b,d,f,g)]	0.544	±	0.039
Dibenz[*a,c*]anthracene[(b,c)]	0.38	±	0.14
Dibenz[*a,h*]anthracene[(b,c)]	0.42	±	0.15
Benzo[*c*]chrysene[(b,f,h)]	0.143	±	0.014
Dibenzo[*b,e*]fluoranthene[(b)]	0.098	±	0.004[(i)]
Naphtho[1,2-*b*]fluoranthene[(b)]	0.635	±	0.020[(i)]
Naphtho[2,3-*b*]fluoranthene[(b)]	0.186	±	0.007[(i)]
Dibenzo[*a,k*]fluoranthene[(b)]	0.057	±	0.007[(i)]
Dibenzo[*j,l*]fluoranthene[(b)]	0.518	±	0.024[(i)]
Dibenzo[*a,l*]pyrene[(b)]	0.076	±	0.007[(i)]
Naphtho[2,3-*e*]pyrene[(b)]	0.227	±	0.004[(i)]
Naphtho[2,1-*a*]pyrene[(b)]	0.438	±	0.017[(i)]
Dibenzo[*e,l*]pyrene[(b)]	0.355	±	0.019[(i)]

(a) The reference mass fraction values, except where otherwise footnoted, are weighted means of the mass fractions from multiple analytical methods [8]. The uncertainty listed with each value is an expanded uncertainty, U, calculated as $U = ku_c$, with coverage factor, $k = 2$. The expanded uncertainty about the mean is determined by combining within method variances with a between method variance [12] following the ISO/JCGM Guide [10,11].
(b) GC/MS (Ia) on a 50 % phenyl-substituted methylpolysiloxane phase after Soxhlet extraction with dichloromethane.
(c) GC/MS (IIa) on a relatively non-polar phase after PFE at 100 °C with dichloromethane at 13.8 MPa.
(d) GC/MS (III) on a 50 % phenyl-substituted methylpolysiloxane phase after PFE at 100 °C with dichloromethane.
(e) The reference value is a weighted mean of average mass fractions, with one average from each of two or more analytical methods [8,9]. The uncertainty listed with each value is an expanded uncertainty, U, calculated as $U = ku_c$, with coverage factor, $k = 2$. The expanded uncertainty is the half width of a symmetric 95 % parametric bootstrap confidence interval [14], which is consistent with the ISO/JCGM Guide [10,11].
(f) GC/MS (Ic) on a dimethyl 50 % liquid crystal polysiloxane phase using same extracts as GC/MS (Ia).
(g) GC/MS (Ib) on a relatively non-polar phase using same extracts as GC/MS (Ia).
(h) GC/MS (IIb) on a relatively non-polar phase after PFE at 200 °C with dichloromethane at 13.8 MPa.
(i) The reference value is the mean of results obtained using one analytical technique. The expanded uncertainty, U, is calculated as $U = ku_c$, where u_c is one standard deviation of the analyte mean, and the coverage factor, $k = 2.48$, is determined from the Student's t-distribution corresponding to the associated degrees of freedom and 95 % confidence level for each analyte.

Table 5b. Reference Mass Fraction Values (Dry Mass Basis) for PAHs in SRM 1648a
Based on Extraction Method and Conditions

Extraction Conditions	Mass Fractions[(a)] (mg/kg)		
PFE at 200 °C			
Naphthalene[(b)]	3.22	±	0.39
Biphenyl[(b)]	1.56	±	0.07
Acenaphthene[(b)]	0.347	±	0.022
Acenaphthylene[(b)]	0.722	±	0.067
Fluorene[(b)]	0.406	±	0.055
Dibenzothiophene[(b)]	0.606	±	0.025
Anthracene[(b)]	1.24	±	0.19
1-Methylphenanthrene[(b)]	2.47	±	0.52
4H-Cyclopenta[*def*]phenanthrene[(b)]	0.362	±	0.055
4-Methylpyrene[(b)]	0.454	±	0.075
Retene[(b)]	0.923	±	0.032
Benzo[*c*]phenanthrene[(b)]	0.584	±	0.067
Benzo[*a*]fluoranthene[(b)]	0.545	±	0.050
Soxhlet extraction or PFE using temperatures between 100 °C and 200 °C			
9-Methylphenanthrene[(b,c,d,e)]	0.46	±	0.11[(f)]
1,7-Dimethylphenanthrene[(b,c,d,e)]	0.260	±	0.021[(f)]
1-Methylfluoranthene[(b,c,d,e)]	0.229	±	0.033[(f)]
3-Methylfluoranthene[(b,c,d,e)]	0.394	±	0.097[(f)]
1-Methylpyrene[(b,c,d,e)]	0.507	±	0.053[(f)]

[(a)] The reference value, except where otherwise footnoted, is the mean of results obtained using one analytical technique. The expanded uncertainty, U, is calculated as $U = ku_c$, where u_c is one standard deviation of the analyte mean, and the coverage factor, $k = 2.48$, is determined from the Student's t-distribution corresponding to the associated degrees of freedom and 95 % confidence level for each analyte.

[(b)] GC/MS (IIb) on a relatively non polar phase after PFE at 200 °C with dichloromethane at 13.8 MPa.

[(c)] GC/MS (Ia) on a 50 % phenyl-substituted methylpolysiloxane phase after Soxhlet extraction with dichloromethane.

[(d)] GC/MS (Ib) on a relatively non-polar phase using same extracts as GC/MS (Ia).

[(e)] GC/MS (IIa) on a relatively non-polar phase after PFE at 100 °C with dichloromethane at 13.8 MPa.

[(f)] The reference values are weighted means of the mass fractions from multiple analytical methods [8]. The uncertainty listed with each value is an expanded uncertainty, U, calculated as $U = ku_c$, with coverage factor, $k = 2$. The expanded uncertainty about the mean is determined by combining within method variances with a between method variance [12] following the ISO/JCGM Guide [10,11].

Reference Mass Fraction Values for Selected Nitro-PAHs: The reference values in Table 6 are the mean of results obtained using one analytical technique, GC/NICI-MS on a 50 % phenyl-substituted methylpolysiloxane phase after Soxhlet extraction with dichloromethane. The measurands are the total mass fraction for each selected Nitro-PAHs in urban particulate matter listed in Table 6 as determined by the method. Metrological traceability is to the SI derived units of mass fraction (expressed as µg/kg on a dry-mass basis). The uncertainty listed with each value is an expanded uncertainty, U, calculated as $U = ku_c$, with coverage factor, $k = 2.48$. The expanded uncertainty is determined by is determined from the Student's t-distribution corresponding to the associated degrees of freedom and 95 % confidence level for each analyte.

Table 6. Reference Mass Fraction Values (Dry-Mass Basis) for Selected Nitro-PAHs in SRM 1648a

	Mass Fractions (µg/kg)		
1-Nitronaphthalene	9.40	±	0.60
2-Nitronaphthalene	7.61	±	0.66
3-Nitrobiphenyl	4.47	±	0.52
5-Nitroacenaphthene	3.50	±	0.30
4-Nitrophenanthrene	1.04	±	0.13
3-Nitrophenanthrene	23.4	±	0.4
9-Nitrophenanthrene	2.70	±	0.35
9-Nitroanthracene	178	±	24
1-Nitropyrene	85.7	±	7.3
2-Nitropyrene	48.7	±	2.0
4-Nitropyrene	8.94	±	0.36
2-Nitrofluoranthene	257	±	26
3-Nitrofluoranthene	6.61	±	0.70
8-Nitrofluoranthene	12.8	±	1.2
7-Nitrobenz[*a*]anthracene	83.0	±	1.1
6-Nitrochrysene	6.75	±	0.55

Reference Mass Fraction Values for Selected PCB Congeners: In Tables 7a and 7b, reference values are provided for selected PCB congeners that are numbered according to the scheme proposed by Ballschmiter and Zell [5] and later revised by Schulte and Malisch [6] to conform with IUPAC rules; differences occur for the following congeners: BZ 107 is IUPAC 109; BZ 199 is IUPAC 200: BZ 200 is IUPAC 201; and BZ 201 is IUPAC 199. When two or more congeners are known to coelute under the conditions used, the PCB congener listed first is the major component and the additional congeners may be present as minor components. The quantitative results are based on the response of the congener listed first. The measurands are the total mass fraction for each PCB congener in urban particulate matter as determined by the method listed in Table 7a and 7b. Metrological traceability is to the SI derived units of mass fraction (expressed as µg/kg).

Table 7a. Reference Mass Fraction Values (Dry-Mass Basis) for Selected PCB Congeners in SRM 1648a

			Mass Fractions[a,b] (µg/kg)		
PCB	8	(2,4'-Dichlorobiphenyl)	11.2	±	0.8
PCB	18	(2,2',5-Trichlorobiphenyl)	12.1	±	0.4
PCB	28	(2,4,4'-Trichlorobiphenyl)[c]	16.1	±	0.9
PCB	31	(2,4',5-Trichlorobiphenyl)[c]	16.3	±	0.8
PCB	44	(2,2'3,5'-Tetrachlorobiphenyl)	10.4	±	0.3
PCB	45	(2,2',3,6-Tetrachlorobiphenyl)	1.50	±	0.10
PCB	49	(2,2'4,5'-Tetrachlorobiphenyl)[b,c]	8.1	±	2.0[d]
PCB	52	(2,2',5,5'-Tetrachlorobiphenyl)[b,c]	16.5	±	6.0[d]
PCB	56	(2,3,3',4'-Tetrachlorobiphenyl)	15.5	±	0.3
	60	(2,3,4,4'-Tetrachlorobiphenyl)			
PCB	66	(2,3',4,4'-Tetrachlorobiphenyl)[b,c]	23.7	±	4.8[d]
PCB	70	(2,3',4',5-Tetrachlorobiphenyl)	26.8	±	1.6
PCB	74	(2,4,4',5-Tetrachlorobiphenyl)	10.1	±	0.2
PCB	82	(2,2',3,3',4-Pentachlorobiphenyl)	3.40	±	0.10
PCB	87	(2,2',3,4,5'-Pentachlorobiphenyl)	11.3	±	0.4
	81	(3,4,4',5-Tetrachlorobiphenyl)			
PCB	92	(2,2',3,5,5'-Pentachlorobiphenyl)	3.60	±	0.10
	84	(2,2',3,3',6-Pentachlorobiphenyl)			
	89	(2,2',3,4,6'-Pentachlorobiphenyl)			
PCB	95	(2,2',3,5',6-Pentachlorobiphenyl)[c]	34.6	±	3.3
PCB	101	(2,2',4,5,5'-Pentachlorobiphenyl)[b,c,e]	34	±	15[f]
PCB	107	(2,3,3',4',5-Pentachlorobiphenyl)	2.80	±	0.21
PCB	118	(2,3',4,4',5-Pentachlorobiphenyl)[b,c,e]	30.5	±	6.0[f]
PCB	128	(2,2',3,3',4,4'-Hexachlorobiphenyl)[b,e]	10.4	±	1.9[d]
PCB	130	(2,2',3,3',4,5'-Hexachlorobiphenyl)	2.25	±	0.10
PCB	137	(2,2',3,4,4',5-Hexachlorobiphenyl)	14.3	±	0.5
PCB	138	(2,2',3,4,4',5'-Hexachlorobiphenyl)[b,c,e]	41	±	12[f]
PCB	146	(2,2',3,4',5,5'-Hexachlorobiphenyl)	5.09	±	0.21

[a] The reference values are the means of results obtained by NIST using one analytical technique. The uncertainty listed with each value is an expanded uncertainty, U, calculated as $U = ku_c$, with coverage factor, $k = 2.48$. The expanded uncertainty about the mean is determined from the Student's t-distribution corresponding to the associated degrees of freedom and 95 % confidence level for each analyte.

[b] Unless otherwise noted, GC/MS (VIa) on a 5 % phenyl-substituted methylpolysiloxane phase after PFE with dichloromethane only.

[c] GC/MS (Va) on a 50 % phenyl-substituted methylpolysiloxane phase after Soxhlet extraction with dichloromethane.

[d] The reference mass fraction values are weighted means of the mass fractions from multiple analytical methods [8]. The uncertainty listed with each value is an expanded uncertainty, $U = ku_c$, about the mean [8,9], with coverage factor, $k = 2$, calculated by combining within method variances with a between method variance [12] following the ISO/JCGM Guide [10,11].

[e] GC/MS (Vb) on a relatively non-polar phase using the same extracts as GC/MS (Va).

[f] The reference value is a weighted mean of average mass fractions, with one average from each of two or more analytical methods [8,9]. The uncertainty listed with each value is an expanded uncertainty, U, calculated as $U = ku_c$, with coverage factor, $k = 2$. The expanded uncertainty is the half width of a symmetric 95 % parametric bootstrap confidence interval [14], which is consistent with the ISO/JCGM Guide [10,11].

Table 7b. Reference Mass Fraction Values (Dry-Mass Basis) for Selected PCB Congeners in SRM 1648a

			Mass Fractions[a,b] (µg/kg)		
PCB	151	(2,2',3,5,5',6-Hexachlorobiphenyl)[b,c]	8.6	±	2.3[d]
PCB	156	(2,3,3',4,4',5-Hexachlorobiphenyl)[b,c]	7.4	±	2.4[d]
PCB	157	(2,3,3',4,4',5'-Hexachlorobiphenyl)	1.54	±	0.10
PCB	158	(2,3,3',4,4',6-Hexachlorobiphenyl)	4.28	±	0.21
PCB	170	(2,2',3,3',4,4',5-Heptachlorobiphenyl)[b,c,e]	24.2	±	6.1[f]
PCB	174	(2,2',3,3',4,5,6'-Heptachlorobiphenyl)	14.7	±	0.5
PCB	175	(2,2',3,3',4,5',6-Heptachlorobiphenyl)	0.55	±	0.10
PCB	176	(2,2',3,3',4,6,6'-Heptachlorobiphenyl)	1.41	±	0.10
PCB	177	(2,2'3,3',4',5,6-Heptachlorobiphenyl)	8.31	±	0.42
PCB	178	(2,2',3,3',5,5',6-Heptachlorobiphenyl)	2.66	±	0.21
PCB	180	(2,2',3,4,4',5,5'-Heptachlorobiphenyl)[c]	45.9	±	2.0
PCB	185	(2,2',3,4,5,5',6-Heptachlorobiphenyl)	1.84	±	0.10
PCB	189	(2,3,3',4,4',5,5'-Heptachlorobiphenyl)	1.29	±	0.10
PCB	191	(2,3,3',4,4',5',6-Heptachlorobiphenyl)	0.96	±	0.10
PCB	200	(2,2',3,3',4,5,6,6'-Octachlorobiphenyl)	1.65	±	0.21
PCB	201	(2,2',3,3',4,5',6,6'-Octachlorobiphenyl)	1.66	±	0.31
PCB	203	(2,2',3,4,4',5,5',6-Octachlorobiphenyl)	29.0	±	4.8
	196	(2,2',3,3',4,4',5,6'-Octachlorobiphenyl)			
PCB	205	(2,3,3',4,4',5,5',6-Octachlorobiphenyl)	1.33	±	0.21
PCB	206	(2,2',3,3',4,4',5,5',6-Nonachlorobiphenyl)[b,c]	12.6	±	2.1[d]
PCB	207	(2,2',3,3',4,4',5,6,6'-Nonachlorobiphenyl)	2.53	±	0.42
PCB	208	(2,2',3,3',4,5,5',6,6'-Nonachlorobiphenyl)	4.9	±	1.5
PCB	209	Decachlorobiphenyl[b,c]	10.5	±	2.6[d]

[a] The reference values are the means of results obtained by NIST using one analytical technique. The uncertainty listed with each value is an expanded uncertainty, U, calculated as $U = ku_c$, with coverage factor, $k = 2.48$. The expanded uncertainty about the mean is determined from the Student's t-distribution corresponding to the associated degrees of freedom and 95 % confidence level for each analyte.

[b] Unless otherwise noted, GC/MS (VIa) on a 5 % phenyl-substituted methylpolysiloxane phase after PFE with dichloromethane only.

[c] GC/MS (Vb) on a relatively non-polar phase using the same extracts as GC/MS (Va).

[d] The reference mass fraction values are weighted means of the mass fractions from multiple analytical methods [10]. The uncertainty listed with each value is an expanded uncertainty, $U = ku_c$, about the mean [10,11], with coverage factor, $k = 2$, calculated by combining within method variances with a between method variance [12] following the ISO/JCGM Guide [10,11].

[e] GC/MS (Va) on a 50 % phenyl-substituted methylpolysiloxane phase after Soxhlet extraction with dichloromethane.

[f] The reference value is a weighted mean of average mass fractions, with one average from each of two or more analytical methods [8,9]. The uncertainty listed with each value is an expanded uncertainty, U, calculated as $U = ku_c$, with coverage factor, $k = 2$. The expanded uncertainty is the half width of a symmetric 95 % parametric bootstrap confidence interval [14], which is consistent with the ISO/JCGM Guide [10,11].

Reference Mass Fraction Values for Additional Chlorinated Pesticides, including Toxaphene Congeners: The measurands are the total mass fraction for each analyte in urban particulate matter as determined by the method indicated listed in Table 8. Metrological traceability is to the SI derived units of mass fraction (expressed as µg/kg on a dry-mass basis).

Table 8. Reference Mass Fraction Values (Dry-Mass Basis) for Additional Chlorinated Pesticides, Including Toxaphene Congeners, in SRM 1648a

	Mass Fractions[(a,b)] (µg/kg)		
alpha-HCH[(b,c)]	8.4	±	2.0[(d)]
gamma-HCH	0.97	±	0.04
hexachlorobenzene[(b,c)]	5.2	±	1.6[(d)]
oxychlordane	3.07	±	0.08
cis-chlordane[(b,e)]	19.1	±	6.8[(d)]
cis-nonachlor	2.16	±	0.22
trans-nonachlor[(b,e)]	14.9	±	3.0[(d)]
trans-chlordane (γ-chlordane)[(b,e)]	29.6	±	1.5[(d)]
mirex	2.23	±	0.47
4,4'-DDE[(c,f)]	20.8	±	9.6[(d)]
2,4'-DDT[(c)]	16.2	±	1.5
4,4'-DDT[(c,f)]	76	±	18[(d)]
2-*endo*,3-*exo*,5-*endo*,6-*exo*,8,8,10,10-octachlorobornane (Parlar 26)	1.78	±	0.15
2-*endo*,3-*exo*,5-*endo*,6-*exo*,8,8,9,10,10-nonachlorobornane (Parlar 50)	7.67	±	0.79
2,2,5,5,8,9,9,10,10-nonachlorobornane (Parlar 62)	8.70	±	0.45
2,2,5-*endo*,6-*exo*,8,9,10-heptachlorobornane (Parlar 32)	10.2	±	0.72
2,2,5,5,9,9,10,10-Octachlorobornane	12.3	±	1.0
2,2,3-*exo*,5,5,9,9,10,10-nonachlorobornane	1.39	±	0.07
2,2,3-*exo*,5,5,8,9,10,10-nonachlorobornane (Parlar 58)	9.27	±	0.67
2,2,3-*exo*,5-*endo*,6-*exo*,8,9,10-octachlorobornane (Parlar 39)	2.31	±	0.17
2-*exo*,3-*endo*,5-*exo*,8,9,10,10-heptachlorobornane	2.88	±	0.22
2,2,5,5,8,9,10-heptachlorobornane	23.2	±	1.5
2,2,3-*exo*,5-*endo*,6-*exo*, 8,9,10,10-nonachlorobornane	2.51	±	0.14
2-*exo*,3,3,5-*exo*,6-*endo*,8,9,10,10-nonachlorobornane	0.563	±	0.054
2,2,5-*endo*,6-*exo*,8,8,9,10,10-nonachlorobornane (Parlar 56)	4.07	±	0.52
2,2,5-*endo*,6-*exo*,8,8,9,10-octachlorobornane (Parlar 42a)	9.34	±	0.65
2-*endo*,3-*exo*,5-*endo*,6-*exo*,8,9,10-heptachlorobornane	1.31	±	0.08
2,2,5-*endo*,6-*exo*,8,9,10,10-octachlorobornane	5.15	±	0.32
2-*endo*,3-*exo*,5-*endo*,6-*exo*,8,9,10,10-octachlorobornane (Parlar 40)	11.1	±	0.7
2,2,3-*exo*,8,8,9,10-heptachlorocamphene (Parlar 25)	2.58	±	0.18
2,2,3-*exo*,8,8,9,9,10-octachlorocamphene (Parlar 31)	1.09	±	0.06
2-*exo*,5,5,8,9,9,10,10-octachlorobornane (Parlar 44)	4.95	±	0.32
2-*exo*,3-*endo*,5-*exo*,8,9,9,10,10-octachlorobornane (Parlar 41)	4.23	±	0.22
2-*endo*,3-*exo*,6-*exo*,8,8,9,10,10-octachlorobornane	10.7	±	0.6

(a) The reference value is the mean of results obtained by NIST using one analytical technique. The uncertainty listed with each value is an expanded uncertainty, U, calculated as $U = ku_c$, with coverage factor, $k = 2.48$. The expanded uncertainty is determined from the Student's t-distribution corresponding to the associated degrees of freedom and 95 % confidence level for each analyte.
(b) Unless otherwise noted, GC/MS (VIb) on a relatively non-polar phase using the same extracts as GC/MS (VIb) only.
(c) GC/MS (VIa) on a 5 % phenyl-substituted methylpolysiloxane phase after PFE with dichloromethane
(d) The reference values, unless otherwise noted, are weighted means of the mass fractions from multiple analytical methods [8]. The uncertainty listed with each value is an expanded uncertainty, U, calculated as $U = ku_c$, with coverage factor, $k = 2$. The expanded uncertainty about the mean is calculated by combining within method variances with a between method variance [12] following the ISO/JCGM Guide [10,11]. The measurand in each case is the mass fraction for each analyte listed based on the method indicated. Metrological traceability is to the unit milligram analyte per kilogram sample on a dry-mass basis.
(e) GC/MS (Vb) on a relatively non-polar phase after Soxhlet extraction with dichloromethane.
(f) GC/MS (Va) on a 50 % phenyl-substituted methylpolysiloxane phase using the same extracts as GC/MS (Vb).

Information Mass Fraction Values for the Content of Selected Elements: Information values that may be of interest and use to the SRM user are given in Table 9. Information values are based on results that did not allow complete assessment of all sources of uncertainty; hence, only estimated means without uncertainties are given. These element values deviate from values in the previous SRM 1648. Scandium (Sc) and thorium (Th) were found to be inhomogeneous at the 5 mg sample size.

Table 9. Information Mass Fraction Values (Dry-Mass Basis) for the Content of Selected Elements

Carbon	Mass Fraction (%)
C_{total} (a,b)	12.7
$C_{organic}$ (b)	10.5
$C_{element}$ (b)	2.3
**Element**	**Mass Fraction (mg/kg)**
Hafnium (Hf) (c)	5.2
Scandium (Sc) (c)	6 to 120
Thorium (Th) (c)	7 to 107

(a) PGAA (NIST)
(b) Thermal-optical organic carbon/elemental carbon method
(c) INAA (NIST)

SUPPLEMENTAL INFORMATION FOR SRM 1648a

Users may wish to refer to the compilation of literature data for the original SRM 1648 for further information on elements that may occur in the material and are not reported in this certificate [13].

Particle Size: Particle size distributions in SRM 1648a determined in aqueous suspension via laser light scattering instrumentation (Malvern Mastersizer 2000) and are shown in Figure 1. The suspensions were prepared by a 10 minute sonication in distilled water (20 mL with approximately 0.02 mg of particulate matter with a drop of 0.1 % solution [volume fraction] of Triton added). These suspensions were gradually introduced into the water-filled, small sample measurement cell until a 6.5 % obscuration of the laser beam was achieved. Each suspension was measured three times for 30 s with a 10 s pause between the passes. A refractive index of 1.52 and absorption index of 0.1 were selected for the measurements. Results were calculated using the General Purpose Model provided by Malvern. The results depicting a typical distribution for total suspended air particulate matter are shown in Figure 1. Uncertainties in these values are estimated at ± 10 % relative (2s).

Table 10. Information Values for Particle Size Characteristics for SRM 1648a[a]

Mean Particle Diameter, d (0.5)[b]	5.85 µm
Particle Diameter, d (0.1)[c]	1.35 µm
Particle Diameter, d (0.9)[d]	30.1 µm

(a) These values are provided for informational purposes only and are based on and instrument-specific measurement of SRM 1648a dispersed in water after 10 minute sonication in water. The values have not been confirmed by an independent analytical technique as required for certification. See Figure 1 for particle-size distribution.
(b) d(0.5) is the particle-size distribution parameter indicating the particle size below which 50 % of the volume is present.
(c) d(0.1) is the particle-size distribution parameter indicating the particle size below which 10 % of the volume is present.
(d) d(0.9) is the particle-size distribution parameter indicating the particle size below which 90 % of the volume is present.

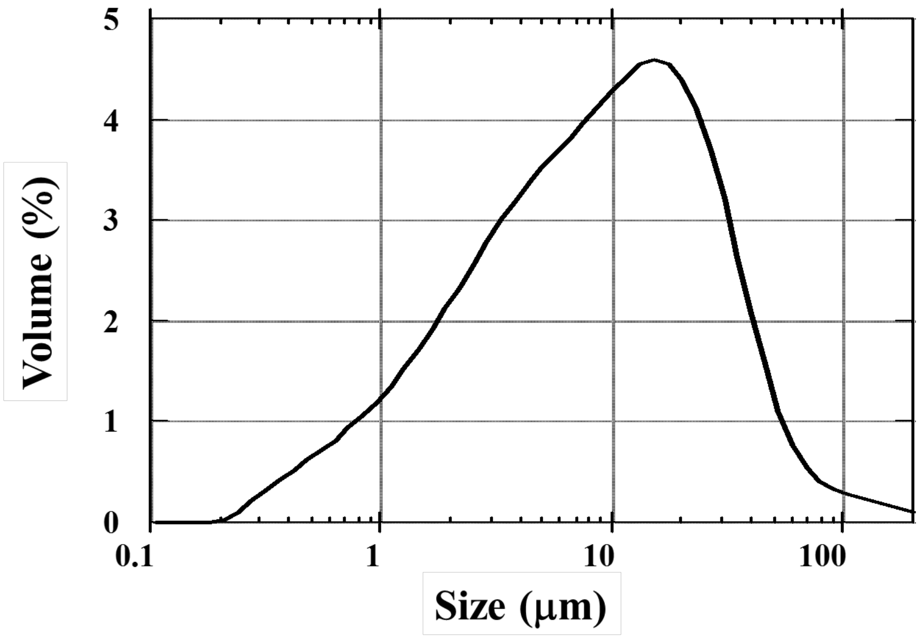

Figure 1. Particle size distribution for SRM 1648a after 10 minute sonication in water. Solid line represents the volume in %. See "Particle-Size Information (Table 10)" for additional information.

Homogeneity Assessment for Inorganic Constituents: Three methods were used to investigate the homogeneity of SRM 1648a for the suggested sample size of several milligrams: small-sample INAA, SS-GFAAS, and WDXRF. Results as relative standard deviations for each of the certified elements are shown in Table 11. The contribution from heterogeneity u_{HET} is derived from the measured total standard deviation u_{exp} and its analytical contribution u_{AN} according to the equation below (equation 1).

$$u_{\mathrm{exp}}^2 = u_{\mathrm{HET}}^2 + u_{\mathrm{AN}}^2 \qquad (1)$$

Based on the models linking sample mass (w) to the homogeneity of particulate materials [14], Kurfürst, Grobecker, and Stoeppler have proposed an elemental homogeneity factor H_e that gives the relative standard deviation in percent for the element of interest if 1 mg samples were repeatedly analyzed and no analytical uncertainty were to influence the result (equation 2) [15].

$$H_{\mathrm{e}} = u_{\mathrm{HET}} \sqrt{w} \qquad (2)$$

INAA Determinations: INAA has been shown to be applicable for the determination of heterogeneity in small samples because the small samples, which essentially form point sources, provide for great improvements in the assays [16]. In the case of this INAA procedure, the analytical variance is in many instances dominated by the uncertainty from counting statistics listed as "instrument uncertainty" in Table 11. Duplicate portions from 12 bottles and duplicate portions from one bottle containing samples from 6 randomly selected locations were analyzed by INAA, resulting in thirty-six test portions analyzed and the heterogeneity components were calculated by subtracting the analytical uncertainties from the observed experimental uncertainty.

WDXRF Determinations: WDXRF has been used routinely for homogeneity determinations because of the excellent instrument stability. Since the X-ray intensities are attenuated in the sample, the highest contribution comes from the surface sample layers. The analyzed sample mass varies for each element and was calculated from a sample thickness that contributes to 90 % of the X-ray yield. To obtain sample mass, the calculated thickness was multiplied with the sample area exposed to the excitation beam and multiplied with the sample density. The counting statistics are listed under "instrument uncertainty" in Table 11. WDXRF analyzed samples from 12 bottles in duplicate.

SS-GFAAS Determinations: Solid sampling procedures were used in conjunction with GFAAS by directly weighing 20 µg test portions into the graphite furnace. The instrument uncertainty was determined as repeatability of the determination of 20 µg single-element solution standards. Twelve measurements were made for each test.

Table 11. Homogeneity Test Results Obtained for Certified Elements

Element	Method	Sample Mass (mg)	Observed Uncertainty	Instrument Uncertainty	Other Uncertainty (estimate)	Uncertainty Due to Heterogeneity	Kurfürst Homogeneity Factor
			Relative Standard Deviation				
Al	INAA	1.15	1.1	0.24	0.5	0.91	0.98
	WDXRF	0.13	1.4	0.21	0.2	1.37	0.49
As	INAA	1.15	2.6	0.62	1.5	2.07	2.22
	SS-GFAAS	0.036	11.2	2.70	0.7	10.85	2.06
Br	INAA	1.15	1.2	0.63	0.7	0.68	0.73
Ca	INAA	1.15	1.7	1.32	0.5	1.02	1.09
	WDXRF	1.5	1.4	0.15	0.2	1.38	1.69
Cd	SS-GFAAS	0.037	11.9	2.04	0.7	11.70	2.25
Ce	INAA	1.15	3.1	1.23	1.2	2.58	2.77
Cl	INAA	1.15	1.4	1.17	0.5	0.48	0.52
	WDXRF	0.59	1.3	0.36	0.2	1.23	0.95
Co	INAA	1.15	2.5	0.91	1.2	2.00	2.14
Cr	INAA	1.15	6.4	0.51	1.2	6.27	6.72
Cu	INAA	1.15	9.6	8.34	0.5	4.73	5.07
	SS-GFAAS	0.02	19.9	2.55	0.7	19.72	2.79
Fe	INAA	1.15	2.0	0.41	1.2	1.55	1.66
	WDXRF	17	1.3	0.09	0.2	1.28	5.28
K	INAA	1.15	1.9	1.17	0.5	1.42	1.53
	WDXRF	1.1	1.5	0.32	0.2	1.45	1.52
Mg	INAA	1.15	4.2	3.92	0.7	1.38	1.48
	WDXRF	0.22	1.4	0.42	0.2	1.32	0.62
Mn	INAA	1.15	3.1	0.46	0.5	3.02	3.24
	WDXRF	7.1	2.0	0.28	0.2	1.97	5.25
Na	INAA	1.15	1.6	0.53	0.5	1.41	1.52
	WDXRF	0.13	1.4	0.99	0.2	0.97	0.35
Ni	SS-GFAAS	0.022	14.0	3.98	0.7	13.40	1.99
Pb	SS-GFAAS	0.026	11.4	3.90	0.7	10.69	1.72
Rb	INAA	1.15	10.2	9.13	1.2	4.39	4.70
S	WDXRF	0.59	1.5	0.13	0.2	1.48	1.14
Sb	INAA	1.15	2.8	0.60	1.2	2.46	2.64
Sr	WDXRF	36	1.7	0.25	0.2	1.67	10.02
Ti	INAA	1.15	5.9	4.00	0.7	4.24	4.55
	WDXRF	2.6	1.8	0.40	0.2	1.74	2.81
V	INAA	1.15	1.1	0.51	0.5	0.80	0.85
Zn	INAA	1.15	1.9	0.25	1.2	1.45	1.56
	SS-GFAAS	0.028	14.6	2.38	0.7	14.39	2.41

The results are to some degree dependent on the method used, but in general, follow the predictions of the sampling models. Best estimates for heterogeneity may be obtained by analyses at the desired sample size (e.g., 1 mg) with a method that has smaller than 1 % uncertainties. Nevertheless, WDXRF and particularly GFAAS results, from much smaller sample sizes, fall into the same range as the INAA results obtained with 1.15 mg samples. The results reveal that most of the certified elements exhibit 1 % or less relative uncertainty due to heterogeneity when the recommended sample size of 5 mg is used. A small group of metals, cobalt (Co), chromium (Cr), iron (Fe), manganese (Mn), and titanium (Ti), seem to be affected by about 2 %, and strontium (Sr) by nearly 5 %. However, these uncertainties are included in the expanded uncertainties of the certified values. The values in Table 11 nevertheless can give some guidance to analysts using significantly smaller sample sizes.

REFERENCES

[1] May, W.; Parris, R.; Beck, C.; Fassett, J.; Greenberg, R.; Guenther, F.; Kramer, G.; Wise, S.; Gills, T.; Colbert, J.; Gettings, R.; MacDonald, B.; *Definitions of Terms and Modes Used at NIST for Value-Assignment of Reference Materials for Chemical Measurements;* NIST Special Publication 260-136; U.S. Government Printing Office: Washington, DC (2000); available at https://www.nist.gov/system/files/documents/srm/SP260-136.PDF (accessed Oct 2020).

[2] Schantz, M.M.; Nichols, J.J.; Wise, S.A.; *Evaluation of Pressurized Fluid Extraction for the Extraction of Environmental Matrix Reference Materials*; Anal. Chem., Vol. 69, pp. 4210–4219 (1997).

[3] Schantz, M.M.; McGaw, E.; Wise, S.A.; *Pressurized Liquid Extraction of Diesel and Air Particulate Standard Reference Materials: Effects of Extraction Temperature and Pressure*; Anal. Chem., Vol. 84, pp. 8222–8231 (2012).

[4] Bergvall, C.; Westerholm, R.; *Determination of 252-302 Da and Tentative Identification of 316-376 Da Polycyclic Aromatic Hydrocarbons in Standard Reference Materials 1649a Urban Dust and 1650b and 2975 Diesel Particulate Matter by Accelerated Solvent Extration- HPLC-GC-MS*; Anal. Bioanal. Chem., Vol. 391 pp. 2235–2248 (2008).

[5] Ballschmiter, K.; Zell, M.; *Analysis of Polychlorinated Biphenyls (PCB) by Glass Capillary Gas Chromatography - Composition of Technical Aroclor- and Clophen-PCB Mixtures*; Fresenius Z. Anal. Chem., Vol. 302, pp. 20–31 (1980).

[6] Schulte, E.; Malisch, R.; *Calculation of the Real PCB Content in Environmental Samples. I. Investigation of the Composition of Two Technical PCB Mixtures*; Fresenius Z. Anal. Chem., Vol. 314, pp. 545–551 (1983).

[7] Levenson, M.S.; Banks, D.L.; Eberhardt, K.R.; Gill, L.M.; Guthrie, W.F.; Liu, H.-k.; Vangel, M.G.; Yen, J.H.; Zhang, N.F.; *An Approach to Combining Results from Multiple Methods Motivated by the ISO GUM*; J. Res. Natl. Inst. Stand. Technol., Vol. 105, pp. 571–579 (2000).

[8] DerSimonian, R.; Laird, N.; *Meta-Analysis in Clinical Trials*; Control Clin. Trials, Vol. 7, pp. 177–188 (1986).

[9] Rukhin, A.L.; *Weighted Means Statistics in Interlaboratory Studies*; Metrologia, Vol. 46, pp. 323–331 (2009).

[10] JCGM 100:2008; *Evaluation of Measurement Data — Guide to the Expression of Uncertainty in Measurement* (GUM 1995 with Minor Corrections); Joint Committee for Guides in Metrology (JCGM) (2008); available at https://www.bipm.org/utils/common/documents/jcgm/JCGM_100_2008_E.pdf (accessed Oct 2020); see also Taylor, B.N.; Kuyatt, C.E.; *Guidelines for Evaluating and Expressing the Uncertainty of NIST Measurement Results*; NIST Technical Note 1297; U.S. Government Printing Office: Washington, DC (1994); available at https://www.nist.gov/pml/nist-technical-note-1297 (accessed Oct 2020).

[11] JCGM 101:2008; *Evaluation of Measurement Data — Supplement 1 to the "Guide to the Expression of Uncertainty in Measurement" — Propagation of Distributions using a Monte Carlo Method*; JCGM (2008); available at https://www.bipm.org/utils/common/documents/jcgm/JCGM_101_2008_E.pdf (accessed Oct 2020).

[12] Horn, R.A.; Horn, S.A.; Duncan, D.B.; *Estimating Hetroscedastic Variance in Linear Models*; J. Am. Stat. Assoc., Vol. 70, pp. 380–385 (1975).

[13] Roelandts, I.; Gladney, E.S.; *Consensus Values for NIST Biological and Environmental Standard Reference Materials*; Fresenius J. Anal. Chem., Vol. 360, pp. 327–338 (1998).

[14] Efron, B.; Tibshirani, R.J.; *An Introduction to the Bootstrap*; Chapman & Hall (1993).

[15] Ingamells, C.O.; Switzer, P.; *A Proposed Sampling Constant for Use in Geochemical Analysis*; Talanta, Vol. 20, pp. 547–568 (1973).

[16] Kurfürst, U.; Pauwels, J.; Grobecker, K.-H.; Stoeppler, M.; Muntau, H.; *Micro-heterogeneity of Trace Elements in Reference Materials - Determination and Statistical Evaluation*; Fresenius J. Anal. Chem., Vol. 345, pp. 112–120 (1993).

[17] Zeisler, R.; *Investigations by INAA for the Development of Natural Matrix Standard Reference Materials (SRMs) Suitable for Small Sample Analysis*; J. Radioanal. Nucl. Chem., Vol. 245, No. 1, pp. 81–85 (2000).

Certificate Revision History: 06 October 2020 (Editorial changes); **18 September 2015** (Values added for mercury and organic constituents; recommend minimum sample sizes updated; editorial changes); **05 January 2012** (Correction of values for organic carbon and elemental carbon in Table 9; editorial changes); **08 April 2008** (Original certificate date).

Users of this SRM should ensure that the Certificate of Analysis in their possession is current. This can be accomplished by contacting the SRM Program: telephone (301) 975-2200; fax (301) 948-3730; e-mail srminfo@nist.gov; or via the Internet at https://www.nist.gov/srm.
