# Supplementary material for: Co-exposure to urban particulate matter and aircraft noise adversely impacts the cerebro-pulmonary-cardiovascular axis in mice
Source: Redox Biol. 2022 Dec 18;59:102580. doi: 10.1016/j.redox.2022.102580 (PMC9804249; doi:10.1016/j.redox.2022.102580)
Supplement: Multimedia component 3 [file mmc3.pdf]

National Institute of Standards & Technology

Certificate of Analysis

Standard Reference Material® 2786

Fine Atmospheric Particulate Matter
(Mean Particle Diameter < 4 µm)

This Standard Reference Material (SRM) is intended for use in evaluating analytical methods for the determination of selected polycyclic aromatic hydrocarbons (PAHs), nitro-substituted PAHs (nitro-PAHs), polybrominated diphenyl ether (PBDE) congeners, hexabromocyclododecane (HBCD) isomers, sugars, polychlorinated dibenzo-*p*-dioxin (PCDD) and dibenzofuran (PCDF) congeners, inorganic constituents, and particle-size characteristics in atmospheric particulate material and similar matrices. All of the constituents for which certified and non-certified values are provided are naturally present in the particulate matter. A unit of SRM 2786 consists of one bottle containing between 100 mg and 140 mg of particulate matter.

Certified Mass Fraction Values: Certified mass fraction values for PAHs and PBDE congeners are provided in Tables 1 and 2, respectively. The certified mass fraction values for selected inorganic constituents are provided in Table 3. The certified values are based on the agreement of results obtained at NIST using multiple analytical techniques, or for cadmium, lead, and mercury, measurements performed at NIST using a single primary method. A NIST certified value is a value for which NIST has the highest confidence in its accuracy in that all known or suspected sources of bias have been investigated or taken into account [1]. The measurand is the total concentration of each analyte. Metrological traceability is to the International System of Units (SI) unit of mass expressed as derived unit of mass fraction.

Non-Certified Values: Non-certified mass fraction values are provided in Table 4 for nitro-PAHs, in Table 5 for additional PAHs, in Table 6 for additional PBDE congeners, in Table 7 for selected inorganic constituents, in Table 8 for selected sugars, and in Table 9 for selected PCDD and PCDF congeners and the total tetra-, penta-, hexa-, and hepta-substituted congeners of PCDD and PCDF. Non-certified mass fraction values for three HBCD isomers are provided in Table 10 and additional inorganic constituents in Table 11. Non-certified values for particle-size characteristics are in Table 12. Non-certified values are estimates of the true value; however, the values do not meet the NIST criteria for certification and are provided with associated uncertainties that may reflect only measurement precision, may not include all sources of uncertainty, or may reflect a lack of sufficient statistical agreement among multiple analytical methods [1]. The measurand is the concentration of each analyte, as determined by the methods indicated in the text. Non-certified values are traceable to the measurement processes and standards used by NIST.

Period of Validity: SRM 2786 is valid, with the measurement uncertainty specified, until **01 May 2031**, provided the SRM is handled and stored in accordance with instructions given in this certificate (see “Instructions for Handling, Storage, and Use”). The value assignments are nullified if the SRM is damaged, contaminated, or otherwise modified.

Overall direction and coordination of technical measurements leading to certification were performed by L.C. Sander and S.A. Wise of the NIST Chemical Sciences Division, and M.M. Schantz, R. Zeisler, S.E. Long, formerly of NIST.

Carlos A. Gonzalez, Chief
Chemical Sciences Division

Gaithersburg, MD 20899

Steven J. Choquette, Director
Office of Reference Materials

Certificate Issue Date: 02 July 2021
Certificate Revision History on Last Page

Maintenance of SRM Certification: NIST will monitor this SRM over the period of its validity. If substantive technical changes occur that affect the certification before the expiration of this certificate, NIST will notify the purchaser. Registration (see attached sheet or register online) will facilitate notification.

Partial funding support for the preparation and certification of this SRM was provided by the U.S. Environmental Protection Agency, Office of Research and Development, Human Exposure and Atmospheric Science Division, National Exposure Research Laboratory, Research Triangle Park, NC.

The particulate matter was collected by J. Kucera and staff from the Nuclear Physics Institute, Prague, Czech Republic. The collection, processing, and bottling of fractions of the particulate matter were performed by M. Mildner, R. Oflaz, B.J. Porter, M.M. Schantz, and R. Zeisler, all formerly of the NIST Chemical Sciences Division.

Analytical measurements at NIST were performed by J.R. Kucklick, K.E. Murphy, and S.A. Rabb of the NIST Chemical Sciences Division, and D. Cleveland, J.M. Keller, S.E. Long, R. Oflaz, B.J. Porter, M.M. Schantz, S.S. Vander Pol, and R. Zeisler, formerly of NIST. The sugar measurements were performed by P. Louchouarn of the Department of Marine Science and Department of Oceanography, Texas A&M University, Galveston, TX and College Station, TX, and the PCDD and PCDF measurements were performed by G. Poole of Environment Canada, Environmental Technology Centre, Analysis and Air Quality Division, Ottawa, Ontario, Canada.

Evaluation of the data for cadmium, lead and nickel was provided by A.L. Pintar of the NIST Statistical Engineering Division (SED). Evaluation of the data for all other constituents was provided by N.A. Heckert of the NIST SED and S.D. Leigh, formerly of NIST.

Support aspects involved in the issuance of this SRM were coordinated through the NIST Office of Reference Materials.

INSTRUCTIONS FOR HANDLING, STORAGE, AND USE

Handling: This material may contain constituents of unknown toxicity and is readily aerosolized. The particle size of a significant portion of the material is in the range of inhalable airborne particulate matter. Therefore, caution and care should be exercised during its handling and use.

Storage: SRM 2786 is provided in an amber glass bottle and should be stored at approximately 25 °C (room temperature) and away from direct sunlight.

Use: Prior to removal of test portions for analysis, the contents of the bottle should be mixed thoroughly. The recommended minimum sample size is 30 mg. The concentrations of constituents in SRM 2786 are reported on a dry-mass basis. The SRM, as received, contains a mass fraction of approximately 1.7 % moisture. A separate test portion of the SRM should be removed from the bottle at the time of analysis and dried to determine the moisture content to convert the mass fraction to a dry-mass basis.

PREPARATION AND ANALYSIS[(1)]

Sample Collection and Preparation: This SRM was prepared from atmospheric particulate material collected in 2005 from an air intake filtration system of a major exhibition center in Prague, Czech Republic. While the sample is not intended to be representative of the area from which it was collected, it should generally typify atmospheric particulate matter obtained from an urban area. The particulate material was removed from the reusable surface filters and sent to NIST. A particle suspension unit and ultra-high-volume sampler (UHVS) were used to resuspend the total suspended particulate matter, and the face velocity of the cyclone in the UHVS was adjusted to control the particle size collected on Teflon membrane filters. The size-fractionated particulate matter was then brushed from the filters and collected in a clean amber bottle with a Teflon-lined cap. The bottle containing the material was rolled and then the material was aliquoted into clean amber 4 mL bottles with Teflon-lined caps. Each bottle contains between 100 mg and 140 mg of material.

[(1)]Certain commercial equipment, instruments, or materials are identified in this certificate to adequately specify the experimental procedure. Such identification does not imply recommendation or endorsement by the National Institute of Standards and Technology, nor does it imply that the materials or equipment identified are necessarily the best available for the purpose.

Conversion to Dry-Mass Basis: The results for the constituents in SRM 2786 are reported on a dry-mass basis; the material "as received" contains residual moisture. The amount of moisture in SRM 2786 was determined by measuring the mass loss after freeze-drying test portions of 100 mg to 140 mg for 5 days at 1 Pa with a –20 °C shelf temperature and a –50 °C condenser temperature. The mass fraction of moisture in SRM 2786 at the time of certification analyses was 1.7 % ± 0.5 % at the 95 % confidence level.

PAHs, Nitro-PAHs, and PBDEs: The general approach used for the value assignment of the PAHs, nitro-PAHs, and PBDEs in SRM 2786 was similar to that reported for the recent certification of several environmental-matrix SRMs [2] and consisted of combining results from analyses using various combinations of different extraction techniques, cleanup/isolation procedures, and chromatographic separation and detection techniques.

Five sets of gas chromatography/mass spectrometry (GC/MS) results, designated as GC/MS (I) through GC/MS (V) were obtained at NIST. For GC/MS (I) analyses, duplicate test portions of between 10 mg and 30 mg from six bottles of SRM 2786 were extracted using pressurized fluid extraction (PFE) at 150 °C with toluene. The extract was fractionated using an aminopropyl solid-phase extraction (SPE) column to isolate the fraction of interest. The processed extract was then analyzed by GC/MS using a 0.25 mm i.d. × 60 m fused silica capillary column with a 50 % (mole fraction) phenyl methylpolysiloxane phase (0.25 µm film thickness; DB-17, Agilent Technologies, Wilmington, DE) and a 0.25 mm i.d. × 15 m fused silica capillary column with a 50 % (mole fraction) liquid crystal polysiloxane phase (0.15 µm film thickness; LC-50, J&K Scientific, Milton, Ontario, Canada). The PAHs were determined on the DB-17 column using electron impact MS (EI-MS), method GC/MS (Ia). The PAHs were also determined on the LC-50 column using EI-MS, method GC/MS (Ib). The nitro-PAHs and PBDEs were determined on the LC-50 column using negative chemical ionization MS (NCI-MS), method GC/MS (Ic).

For the GC/MS (II) determination of the PAHs, one test portion (100 mg) from each of six bottles was extracted using PFE at 100 °C with dichloromethane (DCM). Size exclusion chromatography (SEC) on a divinylbenzene-polystyrene column (10 µm particle size, 10 nm [100 angstrom] pore size, 7.5 mm i.d. × 300 mm, PL-Gel, Polymer Labs, Inc., Amherst, MA) was used. The fraction of interest was further isolated using an alumina (5 % deactivated) SPE column. The isolated fraction was then analyzed by GC/MS using a 0.25 mm i.d. × 60 m fused silica capillary column with a 50 % phenyl methylpolysiloxane phase (0.25 µm film thickness; DB-17MS, Agilent Technologies).

The GC/MS (III) analyses focused on the PBDEs. Six test portions (between 100 mg and 140 mg) were extracted using PFE at 100 °C with DCM. The extracts were cleaned up using an alumina (5 % deactivated) SPE column. SEC on a divinylbenzene-polystyrene column (10 µm particle size, 10 nm (100 angstrom) pore size, 7.5 mm i.d. × 300 mm, PL-Gel, Polymer Labs, Inc.) was then used. This step was followed by an acidified silica SPE column step. Two fractions were collected from the acidified silica column. The PBDEs were quantified using GC/EI-MS on a 0.18 mm i.d. × 30 m fused silica capillary column with a 5 % (mole fraction) phenyl methylpolysiloxane phase (0.18 µm film thickness; DB-5MS, Agilent Technologies). The PBDEs were also quantified using GC/NCI-MS on a 0.18 mm i.d. × 10 m fused silica capillary column with a 5 % (mole fraction) phenyl methylpolysiloxane phase (0.18 µm film thickness; DB-5MS, Agilent Technologies).

For GC/MS (IV), duplicate test portions of between 10 mg and 30 mg from three bottles of SRM 2786 were extracted using PFE at 100 °C (for one of the test portions from each bottle; GC/MS IVa) or at 150 °C (for the other test portion from each bottle; GC/MS IVb) with toluene. The extract was fractionated using an aminopropyl SPE column to isolate the fraction of interest. The processed extract was then analyzed by GC/MS using a 0.25 mm i.d. × 60 m fused silica capillary column with a proprietary non-polar column (0.25 µm film thickness; DB-XLB, Agilent Technologies, Wilmington, DE) and a 0.25 mm i.d. × 30 m fused silica capillary column with a 50 % (mole fraction) phenyl methylpolysiloxane phase (0.25 µm film thickness; DB-17MS, Agilent Technologies). The PAHs were determined on the DB-XLB column using EI-MS. The nitro-PAHs were determined on the DB-17MS column using NCI-MS.

Duplicate test portions of 50 mg from three bottles of SRM 2786 were extracted using PFE at 100 °C with DCM for GC/MS (V) determination of the PAHs. The fraction of interest was further isolated using an alumina (5 % deactivated) SPE column. The isolated fraction was then analyzed by GC/MS using a 0.25 mm i.d. × 60 m fused silica capillary column with a 50 % phenyl methylpolysiloxane phase (0.25 µm film thickness; DB-17MS, Agilent Technologies).

For the methods described above, selected perdeuterated PAHs, perdeuterated nitro-PAHs, and fluorinated and ^{13}C-labeled PBDEs were added to the particulate matter prior to solvent extraction for use as internal standards for quantification purposes. The non-certified mass fraction values for selected PAHs (biphenyl, dibenzothiophene, phenanthrene, anthracene, 1-methylphenanthrene, 2-methylphenanthrene, 3-methylphenanthrene, 9-methylphenanthrene, 1,7-dimethylphenanthrene, and retene) were significantly higher in the extracts obtained using PFE at 150 °C compared to those obtained using PFE at 100 °C. The mass fraction values listed in Table 5 are those obtained using PFE at 100 °C, which is the typical temperature used for PFE, with the values obtained using PFE at 150 °C in footnote (h) of Table 5.

Homogeneity Assessment for PAHs, Nitro-PAHs, and PBDEs: The homogeneity of SRM 2786 was assessed by analyzing duplicate 10 mg to 30 mg test portions from six bottles selected by stratified random sampling. No statistically significant differences among bottles were observed for the three classes of compounds at the 30 mg sample size.

Value Assignment of PAHs and Nitro-PAHs: Recent studies [3,4] on solvent extraction of other air particulate matter SRMs have shown that using PFE at 200 °C removes higher quantities than using PFE at 100 °C or 150 °C for some PAHs and nitro-PAHs. As a result of these studies, SRM 2786 was reanalyzed for determination of PAHs using PFE at 200 °C, and the results were found to be equivalent to the previously assigned values.

HBCDs: The second fraction from the acidified silica SPE clean-up in Method GC/MS (III) above was analyzed by liquid chromatography with tandem mass spectrometry (LC/MS/MS) for the HBCDs using both electrospray ionization (ESI), Method LC/MS/MS IIId, and atmospheric pressurized photoionization (APPI), Method LC/MS/MS IIIe. A C18 column (3.0 mm × 150 mm × 3.5 µm column, Eclipse Plus, Agilent Technologies) was used with a solvent gradient using 2.5 mmol/L ammonium acetate in 12.5 % water in methanol (volume fraction) and acetonitrile at a flow rate of 0.3 mL/min. ^{13}C-labeled HBCDs were added to the air particulate matter prior to solvent extraction for use as internal standards for quantification purposes.

Inorganic Constituents Other Than Mercury: Value assignment of the mass fractions of Cd and Pb were performed using isotope dilution-inductively coupled plasma mass spectrometry (ID-ICP-MS). Two 50 mg samples taken from each of four bottles of SRM 2786 were spiked with ^{111}Cd and ^{206}Pb and subjected to microwave digestion using 1 g of concentrated hydrofluoric acid and 9 g of concentrated nitric acid. For Pb, a portion of the samples were heated to near-dryness and redissolved in 2 % (volume fraction) nitric acid prior to ICP-MS analysis. For Cd, the remaining sample portions were subjected to anion exchange chromatography to separate Cd from the matrix prior to analysis.

Value assignment of the mass fractions of additional inorganic constituents is based on instrumental neutron activation analysis (INAA) and inductively coupled plasma optical emission spectrometry (ICP-OES). For INAA, direct analysis of pellets formed from duplicate test portions (median sample size of 1.3 mg) from each of 12 randomly selected bottles of SRM 2786 was achieved by the comparator technique with established multielement standards. Two sets of irradiations were carried out in the RT-1 pneumatic facility of the NIST Center for Neutron Research (NCNR). The irradiations for short-lived nuclides were done for 300 s. The long irradiations were spaced 48 h apart. Irradiation time was (6+6) h with a 180 degree inversion of the irradiation capsule after the first 6 h for flux homogeneity. For the gamma-ray assay of short-lived nuclides, measurements were done 10 min after 8 min decay. Standards were counted either before or after the samples for 5 min or 10 min respectively. For the long irradiations, the first count was 2 h after 3 d decay, the second count was 12 h after at least 25 d decay with counts of the standards following the sample counts.

For ICP-OES, one 50 mg sample was taken from each of seven bottles of SRM 2786. The samples were microwave-digested using 1 mL of concentrated hydrofluoric acid and 9 mL of concentrated nitric acid. Following dilution with water, the samples were analyzed by ICP-OES using optimized parameters for each element.

Homogeneity Assessment for Inorganic Constituents: The assessment of the homogeneity of SRM 2786 for inorganic constituents was based on the INAA results for the test portions from 12 bottles (median sample size of 1.3 mg). Calculated elemental homogeneity factors H_e [5] range from 1.3 for aluminum to 10 for titanium, which indicates that SRM 2786 is suitable for milligram-size sampling for inorganic constituents.

Mercury: Value assignment of the mass fraction of mercury is based on isotope dilution cold-vapor ICP-MS [6–8]. Single subsamples (40 mg to 70 mg) were taken from each of four bottles and spiked with ^{201}Hg followed by microwave digestion.

Sugars: Value assignment of the mass fractions of sugars is based on the analysis of three samples (30 mg) of SRM 2786 at Texas A&M University [9]. The samples were extracted using PFE at 100 °C with dichloromethane:methanol (9:1, volume fractions). The extracts were dried and redissolved in pyridine and then derivatized using N,O-bis(trifluoroacetamide (BSTFA) containing 1 % (volume fraction) trimethylchlorosilane (TMCS) at 75 °C for 1 h. The sugars were quantified using GC/EI-MS on a 0.25 mm i.d. × 30 m fused silica capillary column with a 5 % (mole fraction) phenyl methylpolysiloxane phase (0.25 µm film thickness, DB-5, Agilent Technologies).

Polychlorinated Dibenzo-*p*-Dioxins and Dibenzofurans: Value assignment of the mass fractions of the seventeen 2,3,7,8-substituted polychlorinated dibenzo-*p*-dioxin and dibenzofuran congeners and the total tetra- through hepta-substituted polychlorinated dibenzo-*p*-dioxins and dibenzofurans is based on the analysis of four test portions (between 100 mg and 150 mg) of SRM 2786 by Environment Canada. Samples were Soxhlet-extracted overnight with toluene. Extracts were concentrated and solvent exchanged to hexane, then passed through a modified silica column followed by a basic alumina column. The dioxin/furan fraction was analyzed by using GC with

high resolution mass spectrometric detection (GC-HRMS) and a 0.25 mm i.d. × 60 m fused silica capillary column with a 5 % (mole fraction) phenyl-substituted methylpolysiloxane phase (0.25 µm film thickness). The 2,3,7,8-tetrachlorodibenzofuran was quantified using a 50 % cyanopropylphenyl-substituted methylpolysiloxane (DB-225, Agilent Technologies) capillary column.

Particle-Size: Particle size distribution measurements for SRM 2786 were carried out using a laser diffraction instrument (Mastersizer 2000, Malvern Instruments, Southborough, MA) and the liquid suspension method with the instrument manufacturer's small-volume sample dispersion unit (Hydro 2000 SM). A suspension of 0.1 % (mass fraction) of SRM 2786 in distilled water with 0.001 % Triton (volume fraction), was prepared by ultrasonication for 1 h. A measurement sequence of background and sample measurement was used. After the recording of the background, a portion of the suspension was added to the measurement cell to achieve an obscuration of 5 %. Three passes of the sample solution were recorded and averaged. A refractive index of 1.5 and absorption index of 0.1 were selected for the measurements. Results were calculated using the General Purpose Model provided by the instrument manufacturer; the results obtained are shown in Figure 1.

Table 1. Certified Mass Fraction Values for Selected PAHs in SRM 2786 (Dry-Mass Basis)

	Mass Fraction[(a)] (mg/kg)		
Fluorene[(b,c,d,e,f)]	0.195	±	0.014
Fluoranthene[(b,c,d,e,f,g)]	10.28	±	0.36
Pyrene[(b,c,d,e,f,g)]	8.01	±	0.22
Benzo[*ghi*]fluoranthene[(b,c,d,e,f,,g)]	3.158	±	0.098
Benzo[*c*]phenanthrene[(b,c,d,e,f,g)]	1.597	±	0.052
Benz[*a*]anthracene[(c,d,e,f)]	4.82	±	0.17
Chrysene[(c,d)]	6.82	±	0.53
Triphenylene[(c,d,g)]	1.794	±	0.041
Benzo[*b*]fluoranthene[(b,e,f,g)]	7.51	±	0.36
Benzo[*j*]fluoranthene[(b,e,f)]	4.37	±	0.32
Benzo[*k*]fluoranthene[(b,c,d,e,f)]	3.48	±	0.32
Benzo[*a*]fluoranthene[(b,c,d,e,f)]	0.898	±	0.037
Benzo[*e*]pyrene[(b,c,d,e,f,g)]	4.77	±	0.28
Benzo[*a*]pyrene[(b,c,d,e,f,g)]	3.70	±	0.13
Perylene[(b,c,d,e,f,g)]	0.769	±	0.020
Benzo[*ghi*]perylene[(b,c,d,e,f,g)]	5.60	±	0.41
Indeno[1,2,3-*cd*]pyrene[(b,c,d,e,f,g)]	4.87	±	0.36
Dibenz[*a,c*]anthracene[(b,e,f,g)]	0.509	±	0.052
Dibenz[*a,j*]anthracene[(b,c,d,e,f,g)]	0.610	±	0.015
Dibenz[*a,h*]anthracene[(b,e,f,g)]	0.717	±	0.029
Benzo[*b*]chrysene[(b,c,d,e,f,g)]	0.662	±	0.022
Picene[(b,c,d,e,f,g)]	1.242	±	0.031
Coronene[(b,c,d,e,f)]	2.156	±	0.087
Dibenzo[*b,k*]fluoranthene[(b,c,d,e,f)]	1.013	±	0.082
Dibenzo[*a,e*]pyrene[(b,c,d,e)]	0.812	±	0.081

(a) The certified value is a weighted mean of the results from three to six analytical methods [10,11]. The uncertainty listed with each value is an expanded uncertainty about the mean [12], with coverage factor, $k = 2$ (approximately 95 % confidence), calculated by combining a pooled within-method variance with a between-method variance following the ISO/JCGM Guide [12–14].
(b) GC/EI-MS Ia using PFE at 150 °C with toluene followed by analysis on a DB-17 column.
(c) GC/EI-MS IVa using PFE at 100 °C with toluene followed by analysis on a DB-XLB column.
(d) GC/EI-MS IVb using PFE at 150 °C with toluene followed by analysis on a DB-XLB column.
(e) GC/EI-MS V using PFE at 100 °C with DCM followed by analysis on a DB-17MS column.
(f) GC/EI-MS II using PFE at 100 °C with DCM followed by analysis on a DB-17MS column.
(g) GC/EI-MS Ib using PFE at 150 °C with toluene followed by analysis on a LC-50 column.

Table 2. Certified Mass Fraction Values for Selected PBDE Congeners[a] in SRM 2786 (Dry-Mass Basis)

		Mass Fraction[b] (µg/kg)
PBDE 99	(2,2',4,4',5-Pentabromodiphenyl ether)[c,d]	7.60 ± 0.03
PBDE 209	(Decabromodiphenyl ether)[c,d]	243 ± 14

[a] PBDE congeners are numbered according to IUPAC rules.

[b] The certified value is a weighted mean of the results from two analytical methods [10,11]. The uncertainty listed with each value is an expanded uncertainty about the mean [12], with coverage factor, $k = 2$ (approximately 95 % confidence), calculated by combining a pooled within-method variance with a between-method variance following the ISO/JCGM Guide [12–14].

[c] GC/NCI-MS Ic using PFE at 150 °C with toluene followed by analysis on a LC-50 column.

[d] GC/NCI-MS III using PFE at 100 °C with DCM followed by analysis on a DB-5 column (10 m).

Table 3. Certified Mass Fraction Values for Selected Trace Elements in SRM 2786 (Dry-Mass Basis)

	Mass Fraction (mg/kg)
Total Mercury	5.32 ± 0.14[a]
Aluminum	33480 ± 700[b]
Cadmium	4.34 ± 0.07[a]
Chromium	462.2 ± 1.5[b]
Copper	847 ± 13[b]
Lead	286 ± 3[a]
Manganese	780 ± 39[b]
Vanadium	85.5 ± 6.5[b]

[a] The certified mass fraction value is the mean of results obtained using one definitive analytical technique (ID-ICP-MS). The expanded uncertainty, U, is calculated as $U = ku_c$, where u_c is the standard error of the dry-mass corrected analyte mean, and the coverage factor, k, is determined from the Student's t-distribution corresponding to the associated degrees of freedom and a 95 % confidence level for each analyte. The coverage factor is 3.18.

[b] The certified value is a weighted mean of the results from two analytical methods, INAA and ICP-OES [10,11]. The uncertainty listed with each value is an expanded uncertainty about the mean [12], with coverage factor, $k = 2$ (approximately 95 % confidence), calculated by combining a pooled within-method variance with a between-method variance following the ISO/JCGM Guide [12–14].

Table 4. Non-Certified Mass Fraction Values for Selected Nitro-PAHs in SRM 2786 (Dry-Mass Basis)

	Mass Fraction[(a)] (µg/kg)
1-Nitropyrene[(b,c,d)]	85.9 ± 5.5
2-Nitrofluoranthene[(b,c,d)]	330 ± 37
9-Nitroanthracene[(b,c,d)]	841 ± 51
3-Nitrophenanthrene[(c,d)]	32.4 ± 6.0
9-Nitrophenanthrene[(c,d)]	12.3 ± 2.8
3-Nitrofluoranthene[(c,d)]	5.28 ± 0.75
7-Nitrobenz[*a*]anthracene[(b)]	105 ± 4[(e)]

(a) The non-certified value is a weighted mean of the results from two or three analytical methods [10,11]. The uncertainty listed with each value is an expanded uncertainty about the mean [12], with coverage factor, $k = 2$ (approximately 95 % confidence), calculated by combining a pooled within-method variance with a between-method variance following the ISO/JCGM Guide [12–14].

(b) GC/NCI-MS Ic using PFE at 150 °C with toluene followed by analysis on a LC-50 column.

(c) GC/NCI-MS IVa using PFE at 100 °C with toluene followed by analysis on a DB-17MS column.

(d) GC/NCI-MS Ic using PFE at 150 °C with toluene followed by analysis on a DB-17MS column.

(e) This non-certified value is the mean of results obtained using one analytical technique. The expanded uncertainty, U, is calculated as $U = ku_c$, where u_c is one standard uncertainty of the analyte mean, and the coverage factor, k, is determined from the Student's t-distribution corresponding to the associated degrees of freedom (df = 11) and a 95 % confidence level for each analyte.

Table 5. Non-Certified Mass Fraction Values for Selected PAHs in SRM 2786 (Dry-Mass Basis)

Extraction Conditions	Mass Fraction (mg/kg)
PFE using temperatures between 100 °C and 150 °C	
Naphthalene[a,b,c,d,e]	0.513 ± 0.044[f]
1-Methylnaphthalene[a,b,c,d,e]	0.151 ± 0.013[f]
2-Methylnaphthalene[a,b,c,d,e]	0.278 ± 0.017[f]
1,6-Dimethylnaphthalene[d,e]	0.152 ± 0.017[f]
Acenaphthylene[b,c,d]	0.282 ± 0.019[f]
2-Methylanthracene[e]	0.171 ± 0.005[g]
4H-cyclopenta[*def*]phenanthrene[a,d,e]	0.368 ± 0.038[f]
1-Methylfluoranthene[b,c,d,e]	0.452 ± 0.048[f]
3-Methylfluoranthene[a,b,c,d,e]	0.82 ± 0.17[f]
1-Methylpyrene[a,b,c,d,e]	0.942 ± 0.096[f]
4-Methylpyrene[a,d,e]	1.01 ± 0.13[f]
Cyclopenta[*cd*]pyrene[b,c,d,e]	0.361 ± 0.056[f]
3-Methylchrysene[a,d,e]	0.773 ± 0.035[f]
6-Methylchrysene[a,d,e]	0.651 ± 0.082[f]
Anthanthrene[d]	0.738 ± 0.031[g]
Dibenzo[*a,h*]pyrene[d]	0.269 ± 0.013[g]
PFE using 100 °C	
Biphenyl[b]	0.163 ± 0.018[g]
Dibenzothiophene[b]	0.132 ± 0.005[g]
Phenanthrene[b,d,e]	3.340 ± 0.065[f]
Anthracene[b,d,e]	0.323 ± 0.052[f]
1-Methylphenanthrene[a,b,d]	0.587 ± 0.044[f]
2-Methylphenanthrene[b,d,e]	0.747 ± 0.043[f]
3-Methylphenanthrene[b,d,e]	0.542 ± 0.048[f]
9-Methylphenanthrene[b,d,e]	0.374 ± 0.057[f]
1,7-Dimethylphenanthrene[b,d,e]	0.738 ± 0.095[f]
Retene[b,d,e]	2.88 ± 0.20[f]
PFE using 150 °C	
Biphenyl[a]	0.263 ± 0.025[g]
Dibenzothiophene[a]	0.239 ± 0.007[g]
Phenanthrene[a]	4.14 ± 0.09[g]
Anthracene[a]	0.607 ± 0.066[g]
1-Methylphenanthrene[a]	0.700 ± 0.027[g]
2-Methylphenanthrene[a]	0.988 ± 0.050[g]
3-Methylphenanthrene[a]	0.677 ± 0.057[g]
9-Methylphenanthrene[a]	0.445 ± 0.020[g]
Retene[a]	3.30 ± 0.12[g]

[a] GC/EI-MS Ia using PFE at 150 °C with toluene followed by analysis on a DB-17 column.
[b] GC/EI-MS IVa using PFE at 100 °C with toluene followed by analysis on a DB-XLB column.
[c] GC/EI-MS IVb using PFE at 150 °C with toluene followed by analysis on a DB-XLB column.
[d] GC/EI-MS V using PFE at 100 °C with DCM followed by analysis on a DB-17MS column.
[e] GC/EI-MS II using PFE at 100 °C with DCM followed by analysis on a DB-17MS column.
[f] The non-certified value is a weighted mean of the results from two to five analytical methods [10,11]. The uncertainty listed with each value is an expanded uncertainty about the mean [12], with coverage factor, $k = 2$ (approximately 95 % confidence), calculated by combining a pooled within-method variance with a between-method variance following the ISO/JCGM Guide [12–14].
[g] This non-certified value is the mean of results obtained using one analytical technique. The expanded uncertainty, U, is calculated as $U = ku_c$, where u_c is one standard uncertainty of the analyte mean, and the coverage factor, k, is determined from the Student's t-distribution corresponding to the associated degrees of freedom (df = 2) and a 95 % confidence level for each analyte.

Table 6. Non-Certified Mass Fraction Values for Selected PBDE Congeners[a] in SRM 2786 (Dry-Mass Basis)

		Mass Fraction (µg/kg)		
PBDE 100	(2,2',4,4',6-Pentabromodiphenyl ether)[b,c]	2.34	±	0.42[d]
PBDE 153	(2,2',4,4',5,5'-Hexabromodiphenyl ether)[c]	1.71	±	0.10[e]
PBDE 154	(2,2',4,4',5,6'-Hexabromodiphenyl ether)[c]	0.924	±	0.033[e,f]
PBDE 183	(2,2',3,4,4',5',6-Heptabromodiphenyl ether)[c]	4.67	±	0.22[e]
PBDE 191	(2,3,3',4,4',5',6-Heptabromodiphenyl ether)[c]	0.810	±	0.044[e]

(a) PBDE congeners are numbered according to IUPAC rules.

(b) GC/NCI-MS Ic using PFE at 150 °C with toluene followed by analysis on a LC-50 column.

(c) GC/NCI-MS IIIa using PFE at 100 °C with DCM followed by analysis on a DB-5 column (10 m).

(d) The non-certified value is a weighted mean of the results from two analytical methods [10,11]. The uncertainty listed with each value is an expanded uncertainty about the mean [12], with coverage factor, $k = 2$ (approximately 95 % confidence), calculated by combining a pooled within-method variance with a between-method variance following the ISO/JCGM Guide [12–14].

(e) This non-certified value is the mean of results obtained using one analytical technique. The expanded uncertainty, U, is calculated as $U = ku_c$, where u_c is one standard uncertainty of the analyte mean, and the coverage factor, k, is determined from the Student's *t*-distribution corresponding to the associated degrees of freedom (df = 5) and a 95 % confidence level for each analyte.

(f) The non-certified value for PBDE 154 includes a contribution from PBB 153 (2, 2', 4, 4', 5, 5'-hexabromobipenyl). The quantitative results are based on the response of only PBDE 154 in the calibration standards.

Table 7. Non-Certified Mass Fraction Values for Inorganic Constituents in SRM 2786 (Dry-Mass Basis)

	Mass Fraction[(a)] (mg/kg)
Antimony	192.1 ± 9.4
Arsenic	36.7 ± 1.1
Calcium	73500 ± 1900
Cesium	4.01 ± 0.22
Chlorine	17390 ± 440
Cobalt	19.55 ± 0.96
Iron	48900 ± 2400
Lanthanum	20.72 ± 0.68
Nickel	243 ± 4[(b)]
Samarium	2.840 ± 0.090
Sodium	14920 ± 370
Titanium	2460 ± 140
Zinc	1793 ± 88

(a) Non-certified values are based on the results from the INAA study unless otherwise noted. The associated uncertainty accounts for both random and systematic effects, but because only one method was used, unrecognized biases may exist for some elements in this matrix. The expanded uncertainty in the non-certified value is equal to $U = ku_c$, where u_c is the combined standard uncertainty and k is the coverage factor, both calculated according to the ISO/JCGM Guide [13,14]. The value of u_c is intended to represent, at the level of one standard deviation, the uncertainty in the mass fraction value. Here u_c accounts for within-method variation and material inhomogeneity. The coverage factor, k, is the Student's t-value for a 95 % confidence interval with the corresponding degrees of freedom (df = 23).

(b) The non-certified mass fraction value is the mean of results obtained using one analytical technique (ICP-OES). The expanded uncertainty, U, is calculated as $U = ku_c$, where u_c is the standard error of the dry-mass corrected analyte mean, and the coverage factor, k, is determined from the Student's t-distribution corresponding to six degrees of freedom and a 95 % confidence level for each analyte. The coverage factor k is 2.45.

Table 8. Non-Certified Mass Fraction Values for Selected Sugars in SRM 2786 (Dry-Mass Basis)

	Mass Fraction[(a)] (mg/kg)
Levoglucosan	465 ± 10
Mannosan	94.7 ± 2.2
Glactosan	33.2 ± 1.1

(a) Non-certified values are the means of results obtained by Texas A&M University using one analytical technique. The expanded uncertainty, U, is calculated as $U = ku_c$, where u_c is one standard uncertainty of the analyte mean, and the coverage factor, k, is determined from the Student's t-distribution corresponding to the associated degrees of freedom (df = 2) and 95 % confidence level for each analyte.

Table 9. Non-Certified Mass Fraction Values for Selected Dibenzo-*p*-Dioxin and Dibenzofuran Congeners in SRM 2786 (Dry-Mass Basis)

	Mass Fractions[a] (µg/kg)
2,3,7,8-Tetrachlorodibenzo-*p*-dioxin	0.009 ± 0.001
1,2,3,7,8-Pentachlorodibenzo-*p*-dioxin	0.049 ± 0.002
1,2,3,4,7,8-Hexachlorodibenzo-*p*-dioxin	0.043 ± 0.001
1,2,3,6,7,8-Hexachlorodibenzo-*p*-dioxin	0.081 ± 0.007
1,2,3,7,8,9-Hexachlorodibenzo-*p*-dioxin	0.079 ± 0.012
1,2,3,4,6,7,8-Heptachlorodibenzo-*p*-dioxin	0.670 ± 0.013
Octachlorodibenzo-*p*-dioxin	2.48 ± 0.07
2,3,7,8-Tetrachlorodibenzofuran[b]	0.149 ± 0.010
1,2,3,7,8-Pentachlorodibenzofuran	0.123 ± 0.002
2,3,4,7,8-Pentachlorodibenzofuran	0.260 ± 0.010
1,2,3,4,7,8-Hexachlorodibenzofuran	0.429 ± 0.010
1,2,3,6,7,8-Hexachlorodibenzofuran	0.167 ± 0.004
2,3,4,6,7,8-Hexachlorodibenzofuran	0.157 ± 0.004
1,2,3,7,8,9-Hexachlorodibenzofuran	0.013 ± 0.002
1,2,3,4,6,7,8-Heptachlorodibenzofuran	0.562 ± 0.010
1,2,3,4,7,8,9-Heptachlorodibenzofuran	0.082 ± 0.001
Octachlorodibenzofuran	0.386 ± 0.007
Total Tetrachlorodibenzo-*p*-dioxins	0.41 ± 0.01
Total Pentachlorodibenzo-*p*-dioxins	0.70 ± 0.02
Total Hexachlorodibenzo-*p*-dioxins	1.19 ± 0.05
Total Heptachlorodibenzo-*p*-dioxins	1.19 ± 0.03
Total Tetrachlorodibenzofurans	2.15 ± 0.03
Total Pentachlorodibenzofurans	2.21 ± 0.07
Total Hexachlorodibenzofurans	1.70 ± 0.02
Total Heptachlorodibenzofurans	0.89 ± 0.01
Total Dibenzo-*p*-dioxins[c]	5.96 ± 0.11
Total Dibenzofurans[c]	7.33 ± 0.14

(a) Non-certified values are the means of results obtained by Environment Canada using one analytical technique. The expanded uncertainty, U, is calculated as $U = ku_c$, where u_c is one standard uncertainty of the analyte mean, and the coverage factor, k, is determined from the Student's t-distribution corresponding to the associated degrees of freedom (df = 3) and 95 % confidence level for each analyte.
(b) Confirmation using a 50 % cyanopropylphenylpolysiloxane phase column.
(c) Total of tetra- through octa-chlorinated congeners.

Table 10. Non-Certified Mass Fraction Values for Three HBCD Isomers in SRM 2786 (Dry-Mass Basis)

	Mass Fraction[a] (µg/kg)
alpha-HBCD[b]	150
beta-HBCD[b]	61
gamma-HBCD[b]	350

(a) The non-certified value is a weighted mean of the results from two analytical methods [10,11].
(b) Methods LC/MS/MS IIId (electrospray ionization) and LC/MS/MS IIIe (atmospheric pressurized photoionization).

Table 11. Non-Certified Mass Fraction Values for Inorganic Constituents in SRM 2786 (Dry-Mass Basis)

	Mass Fraction[(a)] (mg/kg)
Hafnium	4.1
Magnesium	8300
Scandium	7.2
Thorium	5.8

(a) The non-certified values are based on the results from the INAA study.

Table 12. Non-Certified Values for Particle Size Characteristics for SRM 2786[(a)]

	Size (µm)
Mean Particle Diameter, d(0.5)[(b)]	2.8
Particle Diameter, d(0.1)[(c)]	0.91
Particle Diameter, d(0.9)[(d)]	6.9
Volume Weighted Mean[(e)]	3.6

(a) Figure 1 is an instrument-specific measurement of SRM 2786 dispersed in water with 1 h sonication.
(b) d(0.5) is a parameter for the particle-size distribution indicating the particle size below which 50 % of the volume is present.
(c) d(0.1) is a parameter for the particle-size distribution indicating the particle size below which 10 % of the volume is present.
(d) d(0.9) is a parameter for the particle-size distribution indicating the particle size below which 90 % of the volume is present.
(e) The volume weighted mean is the particle size in a uniform distribution.

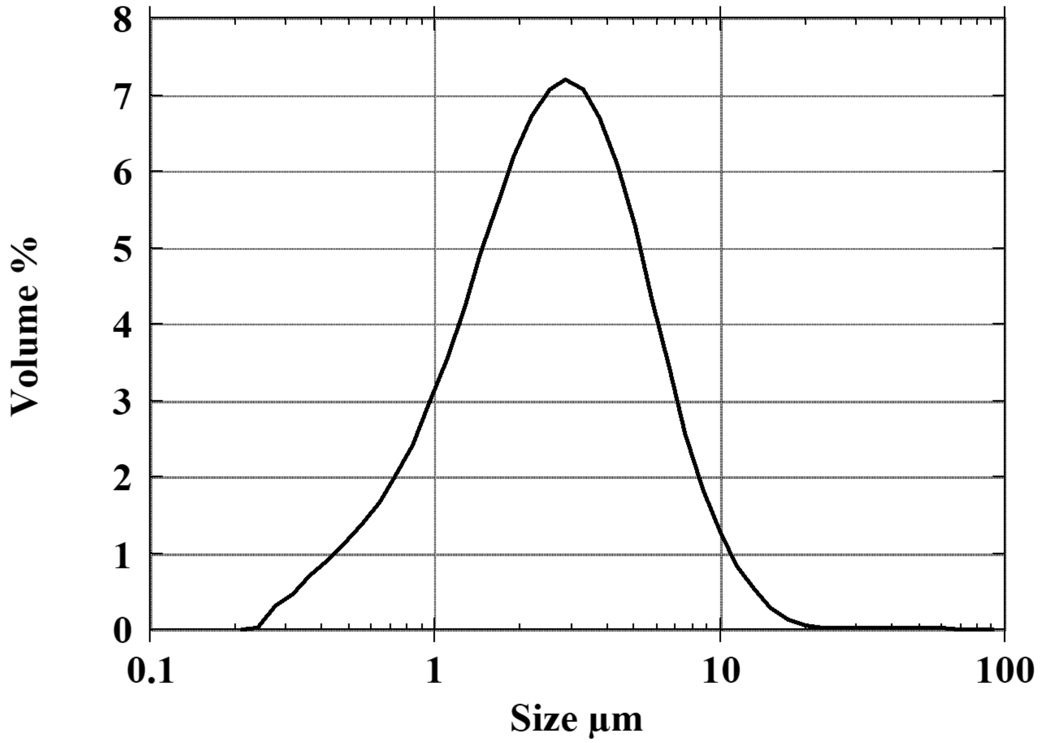

Figure 1. Particle size distribution for SRM 2786 after 1 h. The solid line represents the volume in percentage.

REFERENCES

[1] Beauchamp, C.R.; Camara, J.E.; Carney, J.; Choquette, S.J.; Cole, K.D.; DeRose, P.C.; Duewer, D.L.; Epstein, M.S.; Kline, M.C.; Lippa, K.A.; Lucon, E.; Phinney, K.W.; Polakoski, M.; Possolo, A.; Sharpless, K.E.; Sieber, J.R.; Toman, B.; Winchester, M.R.; Windover, D.; *Metrological Tools for the Reference Materials and Reference Instruments of the NIST Material Measurement Laboratory*; NIST Special Publication 260-136; U.S. Government Printing Office: Washington, DC (2020); available at https://nvlpubs.nist.gov/nistpubs/SpecialPublications/NIST.SP.260-136-2020.pdf (accessed Jul 2021).

[2] Wise, S.A.; Poster, D.L.; Kucklick, J.R.; Keller, J.M.; Vander Pol, S.S.; Sander, L.C.; Schantz, M.M.; *Standard Reference Materials (SRMs) for Determination of Organic Contaminants in Environmental Samples*; Anal. Bioanal. Chem., Vol. 386, pp. 1153–1190 (2006).

[3] Schantz, M.M.; McGaw, E.; Wise, S.A.; *Pressurized Liquid Extraction of Diesel and Air Particulate Standard Reference Materials: Effect of Extraction Temperature and Pressure*; Anal. Chem., Vol. 84, pp. 8222–8231 (2012).

[4] Bergvall, C.; Westerholm, R.; *Determination of 252–302 Da and Tentative Identification of 316–376 Da Polycyclic Aromatic Hydrocarbons in Standard Reference Materials 1649a Urban Dust and 1650b and 2975 Diesel Particulate Matter by Accelerated Solvent Extration – HPLC-GC-MS*; Anal. Bioanal. Chem., Vol. 391, pp. 2235–2248 (2008).

[5] Stoeppler, M.; Kurfürst, U.; Grobecker, K.H.; *Untersuchungen über die Schwermetallanalyse in Feststoffen mit der direkten Zeeman-Atomabsorptions-Spektroskopie*; Fresenius Z.; Anal. Chem., Vol. 322, pp. 687–691 (1985).

[6] Christopher, S.J.; Long, S.E.; Rearick, M.S.; Fassett, J.D.; *Development of Isotope Dilution Cold Vapor Inductively Coupled Plasma Mass Spectrometry and Its Application to the Certification of Mercury in NIST Standard Reference Materials*; Anal. Chem., Vol. 73, pp. 2190–2199 (2001).

[7] Long, S.E.; Kelly, W.R.; *Accurate Determination of Mercury in Coal by Isotope Dilution Cold-Vapor Generation Inductively Cloupled Plasma Mass Spectrometry*; Anal. Chem., Vol. 74, pp. 1477–1483 (2002).

[8] Kelly, W.R.; Long, S.E.; Mann, J.L.; *Determination of Mercury in SRM Crude Oils and Refined Products by Isotope Dilution Cold Vapor ICP-MS Using Closed-System Combustion*; Anal. Bioanal. Chem., Vol. 376, pp. 753-758 (2003).

[9] Louchouarn, P.; Kuo, L-J; Wade, T.L.; Schantz, M.M.; *Determination of Levoglucosan and its Isomers in Size Fractions of Aerosol Standard Reference Materials; Atmospheric Environment*, Vol. 43, pp. 5630–5636 (2009).

[10] DerSimonian, R.; Laird, N.; *Meta-Analysis in Clinical Trials*; Control Clin. Trials, Vol. 7, pp. 177–188 (1986).

[11] Rukhin, A.L.; *Weighted Means Statistics in Interlaboratory Studies*; Metrologia, Vol. 46, pp. 323–331 (2009).

[12] Horn, S.D.; Horn, R.A.; Duncan, D.B.; *Estimating Hetroscedastic Variances in Linear Models*; J. Am. Stat. Assoc., Vol. 70, pp. 380–385 (1975).

[13] JCGM 100:2008; *Evaluation of Measurement Data — Guide to the Expression of Uncertainty in Measurement* (GUM 1995 with Minor Corrections); Joint Committee for Guides in Metrology (2008); available at https://www.bipm.org/en/publications/guides (accessed Jul 2021); see also Taylor, B.N.; Kuyatt, C.E.; *Guidelines for Evaluating and Expressing the Uncertainty of NIST Measurement Results*; NIST Technical Note 1297; U.S. Government Printing Office: Washington, DC (1994); available at https://www.nist.gov/pml/nist-technical-note-1297 (accessed Jul 2021).

[14] JCGM 101:2008; *Evaluation of Measurement Data — Supplement 1 to the "Guide to the Expression of Uncertainty in Measurement" — Propagation of Distributions Using a Monte Carlo Method*; Joint Committee for Guides in Metrology (JCGM) (2008); available at https://www.bipm.org/en/publications/guides (accessed Jul 2021).

Certificate Revision History: 02 July 2021 (Change of expiration date; change of nitro-PAHs certified values to non-certified values due to incomplete stability testing from lack of appropriate calibrants; all other measurands previously labeled as reference or information values were converted to non-certified values; editorial changes); **01 December 2016** (Editorial changes); **13 August 2013** (Extension of certification period; addition of certified values for cadmium and lead; addition of reference value for nickel; change of 9-nitroanthracene certified value to reference value; Table 5 modified to include the reference values previously listed in the footnotes; editorial changes); **15 June 2011** (Original certificate date).

Users of this SRM should ensure that the Certificate of Analysis in their possession is current. This can be accomplished by contacting the SRM Program: telephone (301) 975-2200; e-mail srminfo@nist.gov; or via the Internet at https://www.nist.gov/srm.
