# Supplementary material for: Co-exposure to urban particulate matter and aircraft noise adversely impacts the cerebro-pulmonary-cardiovascular axis in mice
Source: Redox Biol. 2022 Dec 18;59:102580. doi: 10.1016/j.redox.2022.102580 (PMC9804249; doi:10.1016/j.redox.2022.102580)
Supplement: Multimedia component 4 [file mmc4.pdf]

Online supplement

Co-exposure to urban particulate matter and aircraft noise adversely impacts the cerebro-pulmonary-cardiovascular axis in mice

Marin Kuntic[1]*, Ivana Kuntic[1]*, Roopesh Krishnankutty[2]*, Adrian Gericke[3], Matthias Oelze[1], Tristan Junglas[1], Maria Teresa Bayo Jimenez[1], Paul Stamm[1,4], Margaret Nandudu[1], Omar Hahad[1,4], Karin Keppeler[1], Steffen Daub[1], Ksenija Vujacic-Mirski[1], Sanela Rajlic[1,5], Lea Strohm[1], Henning Ubbens[1], Qi Tang[3], Subao Jiang[3], Yue Ruan[3], Kenneth G. Macleod[2], Sebastian Steven[1], Thomas Berkemeier[6], Ulrich Pöschl[6], Jos Lelieveld[7], Hartmut Kleinert[8], Alex von Kriegsheim[2]*, Andreas Daiber[1,4]*, Thomas Münzel[1,4]*

[1] University Medical Center Mainz, Department for Cardiology 1, Molecular Cardiology, Langenbeckstr. 1, 55131 Mainz

[2] Institute of Genetics and Cancer, University of Edinburgh, UK

[3] Department of Ophthalmology, University Medical Center of the Johannes Gutenberg-University Mainz, Langenbeckstr. 1, 55131 Mainz, Germany

[4] German Center for Cardiovascular Research (DZHK), Partner Site Rhine-Main, Mainz, Germany

[5] Department of Cardiothoracic and Vascular Surgery, University Medical Center of the Johannes Gutenberg-University Mainz, Langenbeckstr. 1, 55131 Mainz, Germany

[6] Max Planck Institute for Chemistry, Multiphase Chemistry Department, Mainz, Germany

[7] Max Planck Institute for Chemistry, Atmospheric Chemistry Department, Mainz, Germany

[8] University Medical Center Mainz, Department for Pharmacology, Langenbeckstr. 1, 55131 Mainz, Germany

* These authors contributed equally and should be considered joint first or senior authors.

[#] Addresses for correspondence:

Prof. Dr. Thomas Münzel and Prof. Dr. Andreas Daiber, University Medical Center Mainz, Department of Cardiology, Cardiology I, Geb. 605, Langenbeckstraße. 1, 55131 Mainz, Germany, E-mails: tmuenzel@uni-mainz.de; daiber@uni-mainz.de

Extended Methods

Particulate matter and noise exposure system

The image of the PM and noise exposure system is shown in **suppl. Figure S1**. The system was acquired from TSE Systems GmbH (Hochtaunuskreis, Germany), and consists of a PM source (collision nebulizer), a large drying column, an exposure chamber, a PM monitoring device and a lot of filters and mass flow controllers. The exposure chamber is equipped with two loudspeakers for noise exposure. The system is operated through two separate proprietary software programs installed on a Windows PC, where one operates the PM monitoring device and the other operates the airflow through the system. The most important parts of the system are described in more detail:

1. Collision nebulizer with the drying column – the collision nebulizer is a device used to produce air suspended PM from a water suspended PM. It consists of two main parts, a metal rod through which pressurised air and water suspended PM flow, and a glass jar. High-pressure air flows through the metal rod and sucks in the water suspended PM. The water suspended PM is then carried through the rod and expelled at a right angle towards the wall of the glass jar. The impact of the water suspended PM with the glass jar wall creates a fine mist. The mist droplets are then carried through the drying column where the water phase is absorbed by the silica gel and the PM phase passes through the exposure chamber. The collision nebulizer will handle any PM that can form a stable suspension in water (some stabilizing agents could be added to improve stability). When set up correctly, the collision nebulizer with the drying column is a reliable source of PM.
2. Exposure chamber – the exposure chamber has a volume of 50 L and is constructed from stainless steel. The chamber can hold two standard size mouse cages for a total of 10 mice. The cages have to be made from metal wire and not solid plastic, in order to not interfere with the airflow. The chamber has an inlet port on the top, and an outlet port on the bottom, with many more ports for specific needs. The chamber is equipped with an air humidity and temperature probe, a sampling outlet (sample air can be drawn from the chamber and PM can be caught on a filter for further analysis), a PM monitoring outlet (sample air is drawn continually into the PM monitoring device), a pressure sensor, and many pressure release valves. The chamber is also equipped with a stereo speaker system. Two loudspeakers are located on the top of the chamber and can be used for simultaneous exposure to PM and noise. The inside walls of the chamber are padded with noise insulating material. The chamber can be completely disassembled for cleaning.
3. The PM monitoring device – the NanoSpectroPan is a combination of two spectrometers that can detect PM in size range of 10 nm to 35 µm with 41 increments. The spectrometer used to detect smaller particles in the size range of 10 to 200 nm is based on the electric field mobility principle. The particles entering the detector are charged. The charge, z, roughly corresponds to the surface of the particle. After being charged, the particles migrate through an electric field. The voltage of the electric field is increased in increments from -2 to -320 V. Particles with small m/z (mass divided by charge) will be influenced by the lower voltage electric fields and will not pass

through it. Particles with large m/z will pass through and be recorded as current in a Faraday Cup electrometer. With each incremental increase of the voltage, less and less particles will pass to the electrometer. The resulting currents can then be subtracted to reveal the number of particles in each voltage range. Since the particle mass is proportional to the volume of the particle (radius cubed), and the particle charge is proportional to the surface area (radius squared), the detected m/z is proportional to the radius of the particle. The spectrometer used to detect larger particles in the range from 250 nm to 35 µm is based on the light scattering principle. The particles flow through a tube and pass through a laser light beam. The scattered light is detected at a right angle in relation to the incident light beam. The dilution of particles in air is large enough that almost all particles are detected individually. The intensity of scattered light at the right angle is proportional to the diameter of the particle. The number of scattering events is proportional to the particle concentration. The NanoSpectroPan detector also reports on the total mass concertation of the PM in the exposure chamber based on the mass density of the used PM.

4. Rest of the system – the other notable parts of the system are the mass flow controllers that allow fine tuning of the airflow inside the exposure chamber, filters that collect all of the PM, so it does not escape the system, and the vacuum pump that maintains the negative pressure needed for airflow through the system.

General animal handling and tissue preparation

All animals were treated in accordance with the Guide for the Care and Use of Laboratory Animals as adopted by the U.S. National Institutes of Health and approval was granted by the Ethics Committee of the University Medical Center Mainz and the Landesuntersuchungsamt Rheinland-Pfalz (Koblenz, Germany; permit number: 23 177-07/G 16-1-055 and 20-1-055). During the rest periods mice were housed under a 12-hour light/dark cycle in the ventilated animal cabinet with unlimited access to standard diet and water. After the exposure mice were sacrificed under deep ketamine/xylazine anaesthesia. While under anaesthesia, mice were cut open longitudinally from the pubis to the chin. Ribs and sternum were cut, and the pericardium was opened. Approximately 600 µL of blood were withdrawn from the circulation using a syringe, causing death by exsanguination. Blood was transferred to EDTA containing polypropylene tubes and centrifuged for 10 minutes at 2000 x g. Plasma was collected and stored at -80°C. Next, organs were excised in order: heart, lung, adrenal glands, kidneys, liver, aorta and brain. Organs were transferred to ice-cold Krebs-Hepes buffer. Aortas were cleaned from perivascular adipose tissue under a stereo microscope and cut into pieces for further processing. Other organs were either snap frozen in liquid nitrogen for use in further experiments, fixed in paraformaldehyde for immunohistochemical analysis, embedded in TissueTek for cryo-sectioning or processed immediately.

Particulate matter

In a pilot study, three types of commercially available PM were used for the exposure of mice. All three types of PM were acquired from the National Institute of Standards and

Technology (NIST - US). All of the PM belong to standard reference materials (SRM) and are characterized in detail and supplied with a certificate of analysis. The benefit of having a SRM PM is that they can be purchased many times with a guarantee that the composition is always the same.

NIST 1 (SRM1650b) represents diesel PM and was collected from a heat exchangers of a dilution tube facility following 200 hours of diesel engine run time. The engines were direct injection four-cycle diesel engines, operating at varying conditions, and thus the collected PM is representative of PM produced by heavy-duty diesel engines. The SRM1650b PM was characterised for the presence of PAHs and nitro-substituted PAHs. In addition to PAHs, particle size distribution was analysed and presented as a dependence of total particle volume to the particle size (mass distribution). No metals or other inorganic compounds were certified for the SRM1650b PM. The detailed certificate of analysis can be found on the NIST website: https://www-s.nist.gov/srmors/view_cert.cfm?srm=1650B.

NIST 2 (SRM1648a) represents urban PM that was collected in St. Louis, Missouri during a period of 12 months in 1976 and 1977. The SRM1648a PM was then homogenised and subjected to chemical analysis or inorganic and organic constituents. The PM was characterized for the presence of 37 different elements, selected polycyclic aromatic hydrocarbons (PAHs), nitro-substituted PAHs, polychlorinated biphenyls (PCBs) and chlorinated pesticides. Mass fraction of organic and inorganic carbon are also supplied. The certificate of analysis also comes with the size distribution measurement that is presented as a dependence of total particle volume to the particle size (mass distribution). The detailed certificate of analysis can be found on the NIST website: https://www-s.nist.gov/srmors/view_cert.cfm?srm=1648A.

NIST 3 (SRM2786) represents fine atmospheric PM and was collected in 2005 from an air intake filtration system of a major exhibition centre in Prague, Czech Republic. After the collection, the SRM2786 PM was re-suspended in air and fractionated by size, to be homogenised again later. The SRM2786 PM was characterised for the presence of PAHs, nitro-substituted PAHs, polybrominated diphenyl ethers (PBDEs), hexabromocyclododecane (HBCD) isomers, select sugars (markers of pyrolysis from combustion of organic matter), polychlorinated dibenzi-p-dioxin (PCCD) and dibenzofuran (PCDF) congeners and select inorganic constituents. In addition to composition, particle size distribution was analysed and presented as a dependence of total particle volume to the particle size (mass distribution). The detailed certificate of analysis can be found on the NIST website: https://www-s.nist.gov/srmors/view_cert.cfm?srm=2786.

It is unclear to us at this time why only one of the PM preparations used showed a stable increase in the various measured parameters, although also for NIST3 trends of adverse effects are obvious in most parameters, although not reaching significance. This could be due to composition, as the PM preparations can differ greatly (see very detailed information in the datasheets provided in the online supplement). Particle size could be the reason, but less likely, because all of the PM preparations possess a wide size distribution. An important factor is the time scale of the experiment, as not all components of PM may induce effects during acute exposure. What is important to keep in mind is that we were looking for PM preparation that would show some effect during the acute exposure, so that we could discern

the effects of individual exposures and the combined exposure. The NIST2 preparation notably has the highest load of heavy metals and especially redox-active transition metals (compare the detailed datasheets that were also uploaded as supplemental files), which have often been linked to adverse health effects [1-3]. NIST1 had the weakest effects, which would fit to the fact that these were a kind of "synthetic" particles from industrial diesel engines and the presence of heavy metals or transition metals was not reported for the NIST1 preparation. Because NIST1 were not collected from the environment, as opposed to the urban NIST2 particles and atmospheric NIST3 particles, the lack of atmospheric aging of these soot particles might explain their comparatively low toxicity [4] and the loading with bacterial endotoxins or fungal pyrogens must have been low in these particles as well.

Particulate matter and noise exposure protocol

A total of 172 male C57BL/6J mice were exposed to either PM only, noise only, combination of PM and noise or to fresh air and no noise. In the pilot study comparing the different NIST particle mixtures, a total of 34 mice was used. While the mice were in the exposure chamber, being exposed to PM only or PM and noise, they had access to water, but not to food, as ingestion of PM was not desired. For the exposure with NIST particles only, the mice were exposed for 6 hours per day for 3 days. The actual concentration of NIST2 particles in the chamber was 220 ± 10 μg/m^3. The PM concentration was chosen because 220 ± 10 μg/m^3 is a peak concentration reached in the most polluted cities [5, 6], therefore it is a good concentration to produce reliable acute effects. In addition, since the mice are exposed for only 6 hours per day, the daily concentration is only ¼ of the exposure concentration, which matches the exposures previously used in animal models [7, 8]. Consideration of the different respiratory rats in humans and mice was as follows. Based on the respiratory rate of mice (80-230 min^{-1} reported in https://web.jhu.edu/animalcare/procedures/mouse.html) and tidal volume (0.2 mL reported in [9]), the total mass of PM that passes through the lung of a mouse during a 6 hour exposure session at 200 μg/m^3 is 1.15-3.31 μg. If we assume 100 % retention, that is the total mass of PM that the mouse accumulates. If the average mass of the mouse is 25 g, the total exposure will be 46-132 μg/kg during one 6 hours session. Since the mice are inhaling clean air during the other ¾ of the day, the total daily exposure is still 46-132 μg/kg/day. For humans, the respiratory rate is 10-20 min^{-1} and the tidal volume is 0.5 L. Taking the same 200 μg/m^3 exposure and assuming a body mass of 60 kg, a human would inhale 6-12 μg/kg of PM during a 6 hour interval. Since humans are generally exposed to PM during the whole 24 hour period, this would be extrapolated to 24-48 μg/kg/day. Since mice are exposed during their sleeping phase, we can assume lower range of the respiratory rate, and this would then roughly correspond to the exposure in humans inhaling 200 μg/m^3 during the whole day. These PM concentrations in the air are common in large polluted cities during peak pollution [6].

During the noise only exposure, mice were given the same conditions as the mice exposed to PM only. The aircraft noise was a 2 h long noise pattern of 69 aircraft noise events with a duration of 43 s each and a maximum sound pressure level of 85 dB(A) and an average sound pressure level of 72 dB(A), that was recorded at the Düsseldorf airport and previously used in human [10-12] and animal studies [13-17]. Mice were exposed to this noise recording on a

loop for 9 ± 3 hours per day for 3 days. For a simultaneous exposure of mice to PM and noise, a combined protocol was used for 6 hours per day, for 3 days. All of the exposures were done during the day – mouse sleeping phase.

Blood pressure measurement

Blood pressure was measured by a non-invasive method, using tail-cuff plethysmography. The used blood pressure measurement instrument was CODA (Kent Scientific, Torrington, CT). Before the measurement mice were restrained inside of a plastic tube and placed on a preheated plate (32 °C). After a 15 minute rest two cuffs (occlusion cuff and volume pressure recording cuff) were placed on the tail of each the mouse. The instrument performs 5 iterations of blood pressure measurements for animals to get acclimated to the stress, after which 10 more measurement iterations are performed and recorded. The mean value of the 10 measurements was used. The accuracy of the tail-cuff method was previously confirmed in relation to the radiotelemetry [18]. Before the baseline blood pressure measurement was recorded, mice were trained without recording the results for at least two separate occasions. The final, experimental value of blood pressure (T1) was recorded after the last exposure to PM, noise or PM and noise.

Isometric tension studies

Aortic ring segments from the thoracic part of the aorta, 4 mm in length, were cleaned from the perivascular adipose tissue and suspended from the force transducers in an organ bath. The force exerted by the aortic rings was measured in the presence of varying concentrations of vasoconstrictors and vasodilators. Endothelial function was determined by the addition of endothelium-dependent vasodilator, acetylcholine (Ach). Ach was titrated in the range of 10^{-9} to $10^{-5.5}$ M, to the aortic rings pre-constricted with prostaglandin $F_{2\alpha}$ (yielding approximately 80 % of the maximal tone induced by KCl bolus). Endothelium-independent vasodilation was assessed by titrating the pre-constricted aortic rings with nitro-glycerine (GTN) in the range of 10^{-9} to $10^{-4.5}$ M. A constant temperature of 37 °C and flow of carbogen gas (95% oxygen, 5% CO_2 v/v) was maintained in the organ chamber throughout the experiment. The cyclooxygenase inhibitor indomethacin (10 µM) was added to the buffer to prevent the production of prostaglandins and other vasoactive eicosanoids that might interfere with the measurement [19].

Cerebral and retinal arteriole function measurement

Isolation of cerebral arterioles was recently described by us in detail [20]. Briefly, following isolation of the brain, a vascular tree of the middle cerebral artery (MCA) was isolated using fine-point-tweezers and microscissors. Next, the MCA including its branches was transferred to a pressure myograph. The MCA was then cannulated with a micropipette, and the pipette tip was guided into a branching arteriole by gently pulling the cannulated end of the MCA further on the pipette and by pushing the free end of the MCA against the pipette tip. Once the

arteriole was cannulated, the free end of the MCA was tied to the micropipette. Subsequently, the non-cannulated end of the arteriole was tied to an opposite micropipette, and the pipette tips were gently moved apart to stretch the arteriole. After, the pressure myograph was placed on an inverted microscope, the arteriole was pressurized to 40 mmHg via the indwelling micropipette and visualized by a digital camera. After an equilibration time of 30 minutes, the arteriole was ready for measurement. Preparation of retinal arterioles for measurement has also been previously described in detail by our group [21, 22]. First, the eye was enucleated together with the retrobulbar tissue. Next, the ophthalmic artery and its orbital branches were cleaned from surrounding tissue by using fine-point-tweezers and microscissors and were ligated at their proximal ends with 10-0 nylon monofilament suture. Subsequently, the retrobulbar vasculature was deactivated by immersing the intact eye globe with the attached vasculature for 10 seconds into 70% ethanol. Next, the eye globe was punctured at the limbus with a needle and the cornea, uvea, sclera, and lens were excised apart from a piece of sclera around the optic nerve, which was remained to prevent injury and torsion of the ophthalmic artery. Then, the preparation was placed into an organ chamber, and the ophthalmic artery was cannulated with a micropipette and secured with 10-0 nylon suture. After this procedure, the retina was placed onto a transparent plastic platform, and four radial incisions were made, enabling spreading out the retina and fixing it to the bottom by placing a steel ring on it. Next, retinal vessels were pressurized via the micropipette located in the ophthalmic artery to a level corresponding to 50 mm Hg, equilibrated for 30 minutes before measurement, and imaged under brightfield conditions.

After equilibration, the thromboxane mimetic, U46619 (10^{-11} to 10^{-6} M; Cayman Chemical, Ann Arbor, MI), was cumulatively applied to both cerebral and retinal vascular preparations. After completion of the concentration-response curve, U46619 was washed out and the respective arteriole was preconstricted to 50 –70% of the initial diameter by titration of U46619. Next, responses to the endothelium-dependent vasodilator, acetylcholine, and to the nitric oxide (NO) donor, sodium nitroprusside, (both 10^{-9} to 10^{-4} M; Sigma-Aldrich, Munich, Germany) were tested by cumulative application of the respective substances into the organ chamber. A constant temperature of 37 °C and flow of carbogen gas (95% oxygen, 5% CO2 v/v) was maintained in the organ chamber throughout the experiment.

Dihydroethidium fluorescence microtopography

Aortic segments from the thoracic part of the aorta, 4 mm in length, lung and cortex pieces were embedded in polymeric resin (TissueTek). The embedded tissue pieces were flesh frozen in liquid nitrogen and stored at -80 °C. The frozen blocks containing tissue pieces were cut on a cryo-microtome at -25 °C in order to produce 8 µm thick sections. The sections were transferred onto SuperFrost® microscopy slides and stored at -80 °C for further use. Oxidative stress in the aorta, lung and brain was assessed using dihydroethidium (DHE) - dependent fluorescence microtopography [23]. The tissue-containing slides were incubated with 1 µM DHE for 30 minutes at 37 °C, washed twice with PBS, protected with a cover slide, and imaged under a fluorescence microscope (Axiovert 40CFL with Axiocam MRm, Zeiss, Germany). Selected samples were co-incubated with the NOX inhibitor GSK2795039 (50 µM) or the mitochondrial ROS scavenger mitoTEMPO (2.5 µM). The excitation

wavelength was set to 510 – 520 nm, and red fluorescence was recorded (emission: 580 – 610 nm). The fluorescence images were quantified as the mean pixel intensity obtained from the area of interest (endothelium and media for the aorta, whole vascular wall for cerebral and retinal arterioles, and whole image for lung and cortex), using ImageJ software.

Western blot analysis

Protein expression in tissues of interest was determined by a standard western blot analysis [13]. In brief, the tissue was homogenized in liquid nitrogen using a mortar and a pistil, after which proteins were extracted with the Hg buffer (20 mM Tris-HCl, 250 mM sucrose, 3 mM EGTA, 20 mM EDTA, 0.5 mM PMSF, 1 % (v/v) protease inhibitor cocktail, 1 % (v/v) phosphatase inhibitor cocktail, 1 % (v/v) TritonX-100). Tissue debris was separated from the extracted proteins by centrifugation at 10 000 x g for 10 minutes and 4 °C. Total protein concentration was determined using a Bradford assay. Depending on the tissue, 15 to 35 µg of protein (in reducing conditions: Laemmli buffer with 2-mercaptoehanol and heating to 95 °C for 5 minutes) were loaded on a 10 % polyacrylamide gel and subjected to a SDS-PAGE electrophoresis for 2 hours at 120 V. The proteins were then blotted to a nitrocellulose membrane (Amersham™ Protran® 0.45µm, GE Healthcare) at 120 mA for 2 hours. Position and kDa weight of the proteins was determined using the benchmark unstained protein ladder (10-kDa ladder). Proteins were visualized on the membrane using Ponceau-S staining before the membrane was cut in desired sections. The membrane stripes were blocked with either 3 or 5 % bovine serum albumin or 5 % milk powder for one hour at room temperature and subsequently incubated with the respective primary antibodies overnight at 4 °C. After the incubation with the primary antibody the membranes were washed with either PBS-T or TBS-T. Detection and quantification were performed with peroxidase conjugated anti-mouse/rabbit/goat secondary antibodies (incubated at room temperature for 1-2 hours) and enhanced chemiluminescence kit (Pierce™ ECL Western Blotting Substrate, Thermo Scientific). Densitometric quantification of antibody-specific bands was performed with a ChemiLux Imager and Gel-Pro Analyzer software. Primary antibodies used for detection of proteins of interest were: NOX-2 (BD Bioscience, 611415, 1:500 in 3% BSA/TBS, anti-mouse), HO-1 (Abcam, ab68477, 1:2000 in 5% BSA/TBS, anti-rabbit), ACE-2 (Abcam, ab108252, 1:1000 in 5% Milk/TBS, anti-rabbit), P-MARCKS (Sigma, SAB4300233, 1:500 in 3% BSA/TBS, anti-rabbit), NOX-1 (Abcam, ab131088, 1:2000 in 5 % BSA/PBS, anti-rabbit), Cu/Zn-SOD (Millipore, 07-403, 1:1000 in 3% BSA/TBS, anti-rabbit), and Mn-SOD (Upstate, 06-984, 1:1000 in 3% BSA/TBS, anti-rabbit). Secondary antibodies used for detection were: anti-mouse (Vector, PI-2000, 1:10000 in the same buffer as the primary antibody) and anti-rabbit (Vector, PI-1000, 1:10000 in the same buffer as the primary antibody).

Quantitative reverse transcription real-time PCR (qRT-PCR)

Total mRNA was isolated from the mouse lung and cortex using the standard phenol extraction method. In brief, the tissues were homogenized in a Tissue Lyser (QIAGEN,

Hilden, Germany) using guanidine thiocyanate (GIT) buffer and total mRNA was extracted using phenol/chloroform/isoamyl alcohol. After centrifugation for 20 min at 14,000 x g and 4°C and separation of the aqueous phase, total mRNA was precipitated in 2-propanol at -20 °C overnight. The mRNA was centrifuged for 20 min at 14,000 g and 4°C and washed with ice-cold 80% ethanol. After washing, RNA was dissolved in RNase free water and total concentration was determined by a photometer and purity was checked by measuring the ratio of absorbance are 260 and 280 nm. Total mRNA was diluted to 0,0625 µg/µL, and 2 µL were used per reaction well. The quantitative reverse transcription real-time PCR (qRT-PCR) analysis was done using QuantiTect Probe RT-PCR kit (QIAGEN) [14]. The primer-probe-mixes were purchased from Applied Biosystems (Foster City, CA, USA). The primer-probe-mix is a TaqMan® dual-labeled fluorescence resonance energy transfer (FRET) probe. The specific primers used in this study were: Cybb (Nox2, Mm00432775_m1), Nmox1 (HO-1, Mm00516004_m1), Ace2 (Mm01159003_m1), Ccl2 (MCP-1, Mm00441243_g1) and CD68 (Mm00839636_m1)

ELISA for hormones

Plasma concentrations of hormones adrenaline and noradrenaline was determined using a commercially available enzyme-linked immunosorbent assay (ELISA) kit. The TriCat kit (catalogue # RE59395) was purchased from IBL (Gunma, Japan). The ELISA was performed following the instructions of the vendor that were included with the kit.

Immunohistochemistry

Aortic segments were fixed in 4% paraformaldehyde and embedded in paraffin. Paraffin-embedded aortic samples were cut on a microtome into 10 µm sections and stained with primary antibodies as described [14]. ET-1 staining was performed using a specific antibody (abcam #117757, 1:800). 3-NT staining was performed using a specific antibody (Millipore, Berlington, MA #06-284, 1:200). NOX-2 staining was performed using specific antibody (LSBio, Seattle, WA, #B12365, 1:200). All primary antibodies were used per manufacturers' instructions. A biotinylated secondary antibody (Thermo Fisher Scientific, Waltham, MA) was used at a dilution specified by the manufacturer. For immunochemical detection ABC reagent (Vector, Burlingame, CA, US) and then DAB reagent (peroxidase substrate Kit, Vector) were used as substrates. Images were taken with an inverted bright-field microscope (Olympus, Tokyo, Japan). Quantification was performed using Image ProPlus 7.0 software (Media Cybernetics, Rockville, MD, US), calculating the percentage of the aortic area that was stained. A threshold was selected for the specific staining colour and all area that had greater intensity in that specific brown colour than the defined threshold was included in the calculation process with the software. The calculated stained area was normalized to the total area of the aortic tissue in a respective image yielding a percentage of stained area.

Cytokine array

The cytokine assay was carried out in microarray format using validated capture/detection antibody pairs (R&D Systems). For each sample, a selected panel of 64 capture antibodies was printed as four-replicate sub-array sets on a single nitrocellulose-coated glass slide (Supernova Grace Biolabs) using a Quanterix-2470-microarrayer and 185uM printing pins. Each sample was incubated overnight with a 64 sub-array slide. After washing and blocking steps each slide was clamped onto a multi-chamber gasket and each sub-array was incubated with the appropriate biotin-labelled detection antibody. A final incubation with fluorescently labelled streptavidin followed by slide scanning using an InnoScan710IR scanner (Innopsys) generated array images. Images were analyzed and signals quantified using Mapix software (Innopsys).

Transcriptomics

RNA sequencing was carried out at Novogene Bioinformatics Technology Co., Ltd., in Cambridge (UK). According to the manufacturer's protocol, aortic mRNA was isolated using the RNeasy Mini kit (Qiagen, Hilden, Germany). RNA sample quality control, mRNA library preparation (poly A enrichment), NovaSeq PE150 sequencing (9 G raw data per sample), data quality control and basic data analysis were performed by Novogene Bioinformatics Technology Co., Ltd., in Cambridge (UK). All analyses of RNA-Seq data were performed with CLC genomic workbench 21.0.5 from Qiagen (Hilden, Germany; using the parameters provided by the manufacturer. The data (fastq.gz) were imported in the CLC data format (.clc). Then the reads were trimmed using the parameter provided by the manufacturer (quality score: 0.05; maximum number of ambiguities: 2). These trimmed RNA-Seq data were mapped to the murine genome (Mus-musculus; GRCm39) using the parameters provided by the manufacturer (Mismatch cost: 2; Insertion cost: 3; Deletion cost: 3; Length fraction: 0.8; Similarity fraction: 0.8; Maximum number of hits for a read: 10). Also, the parameters used for the calculation of gene expression were used as provided by the program (Strand setting: Both; Library type setting: Bulk) and rpkm data [24] were calculated. The rpkm data were used for calculation of the fold enhancement of the mRNA expression. The differentially expressed genes between groups from single or combined exposures were visually represented as volcano plots and the online tool VolcaNoseR [25] was used to generate the plots (**suppl. Figure S6, S7 and S8**).

Bioinformatical analysis of transcriptomic data

To generate a visual heatmap representation of the clustered matrix the hierarchical clustering function in perseus [26] was used. The clusters of highly correlated genes contributing to the distinct and combined effects were identified for further analysis. The protein-protein interaction (PPI) network was built using an online tool STRING (http://string-db.org/) and visualized via Cytoscape software (version 3.9.1; http://cytoscape.org/). KEGG (Kyoto Encyclopedia of Genes and Genomes) pathway enrichment analysis was performed using the Cytoscape Bingo plugin and MCODE plugin was used for clustering the network. $P < 0.05$ was considered statistically significant for KEGG analysis.

Statistical analysis

Where possible, the results are presented as either bar graphs with individual values or bar graphs showing mean ± SEM. Two-way ANOVA (with Tukey's correction for comparison of multiple means) was used for comparisons of concentration-relaxation curves. One-way ANOVA (with Tukey's post-hock analysis for comparison of multiple means) or, where appropriate, equivalent non-parametric Kruskal-Wallis test (Dunn multiple comparison) was used for comparisons of all other data where more than 2 groups were present. T-test was used for comparison if only 2 groups were used. All statistical analysis was performed in Prism for Windows, version 9. The numerical value of the p-value is either used directly or a star signifies a $p\text{-value} < 0.05$, that was considered as statistically significant. The number of replicates in the different assays may vary since not all animals were used in all assays.

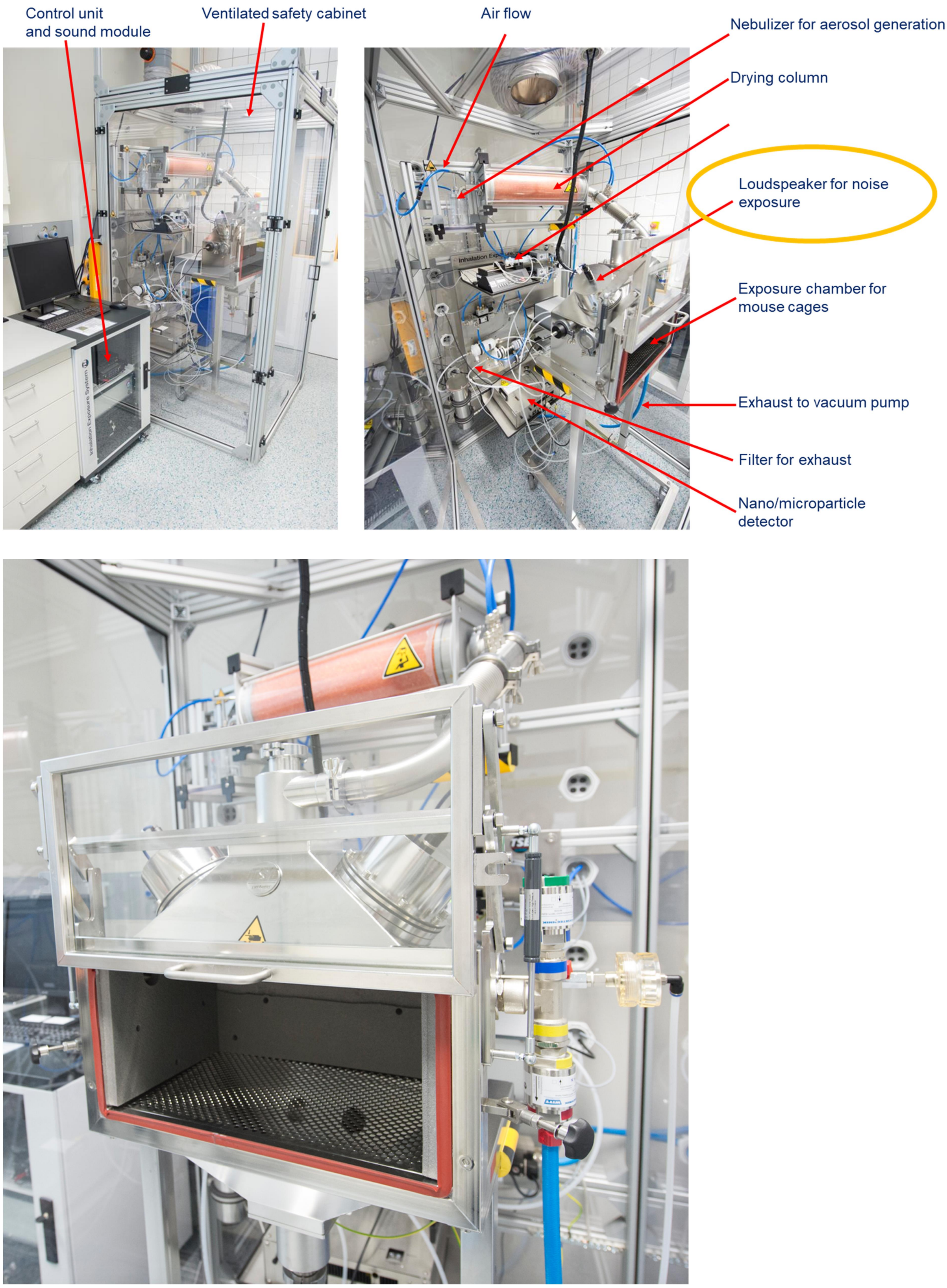

Figure S11. The PM and Noise exposure system. Photograph of the PM and noise exposure system with the different parts labelled. Zoom photograph of the exposure chamber.

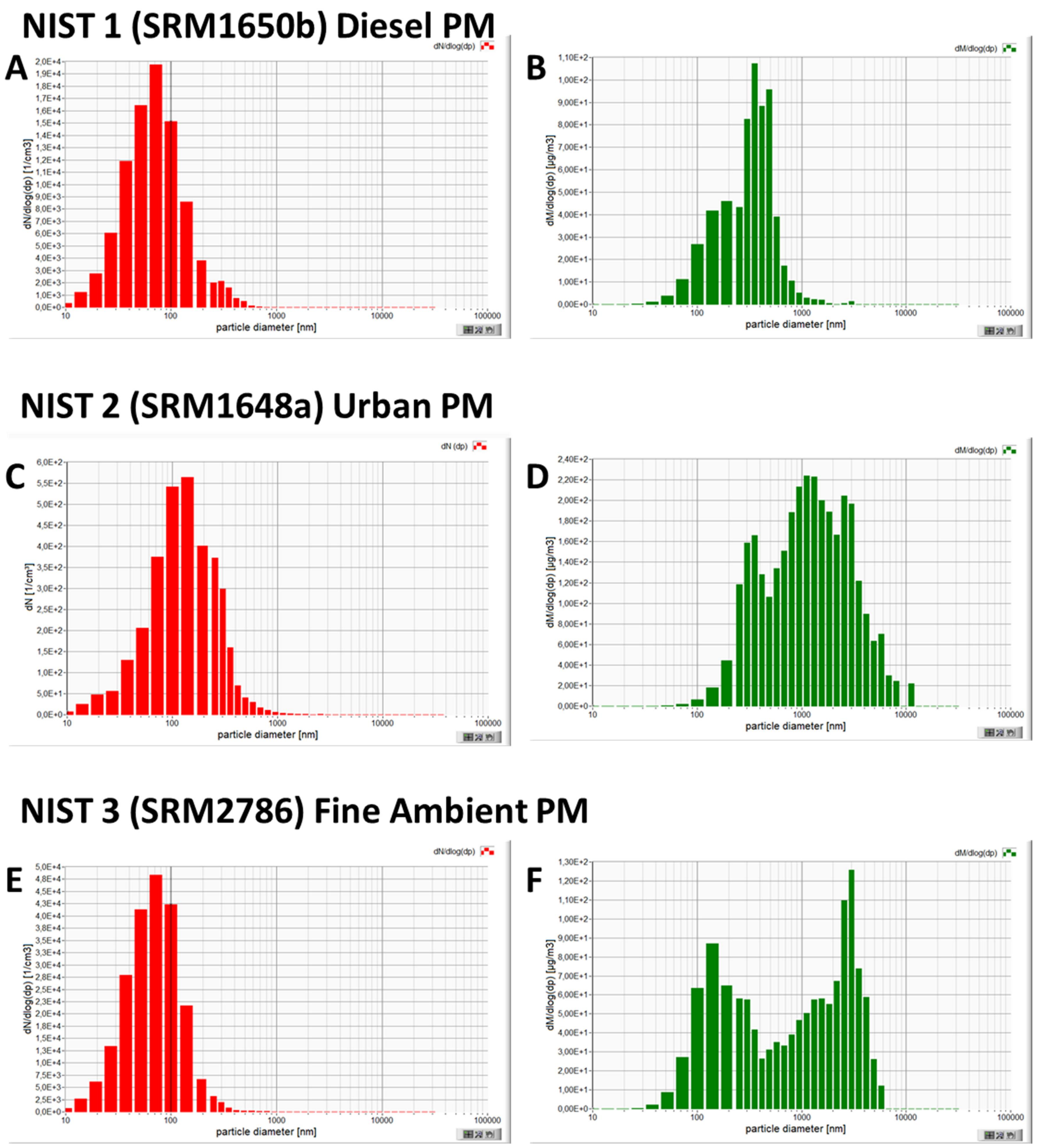

Figure S2. The size distribution of different NIST PM preparations used within this work. For each PM preparation, two distributions are shown. The first one is the number distribution, where the number of particles are shown on the y-axes and the particle diameter is shown on the x-axes. The second one is the mass distribution, where the mass of particles is shown on the y-axes and the particle diameter is shown on the x-axes. NIST 1 (SRM 1650b) number distribution (A), and mass distribution (B). NIST 2 (SRM 1648a) number distribution (C), and mass distribution (D). NIST 3 (SRM 2786) number distribution (E), and mass distribution (F). The certificates of analysis found on the NIST website, describe the volume distribution (mass distribution of particles) for each preparation. Only the certificate for NIST1 PM preparation states that it has a bimodal distribution, while the other two PM preparations do not. The clearly multimodal distributions seen in B, D and F are an artefact of low sampling rate and of the different sensitivities of the two spectrometers (described below) at their respective upper and lower diameter limits. All three preparations could be considered $PM_{2.5}$.

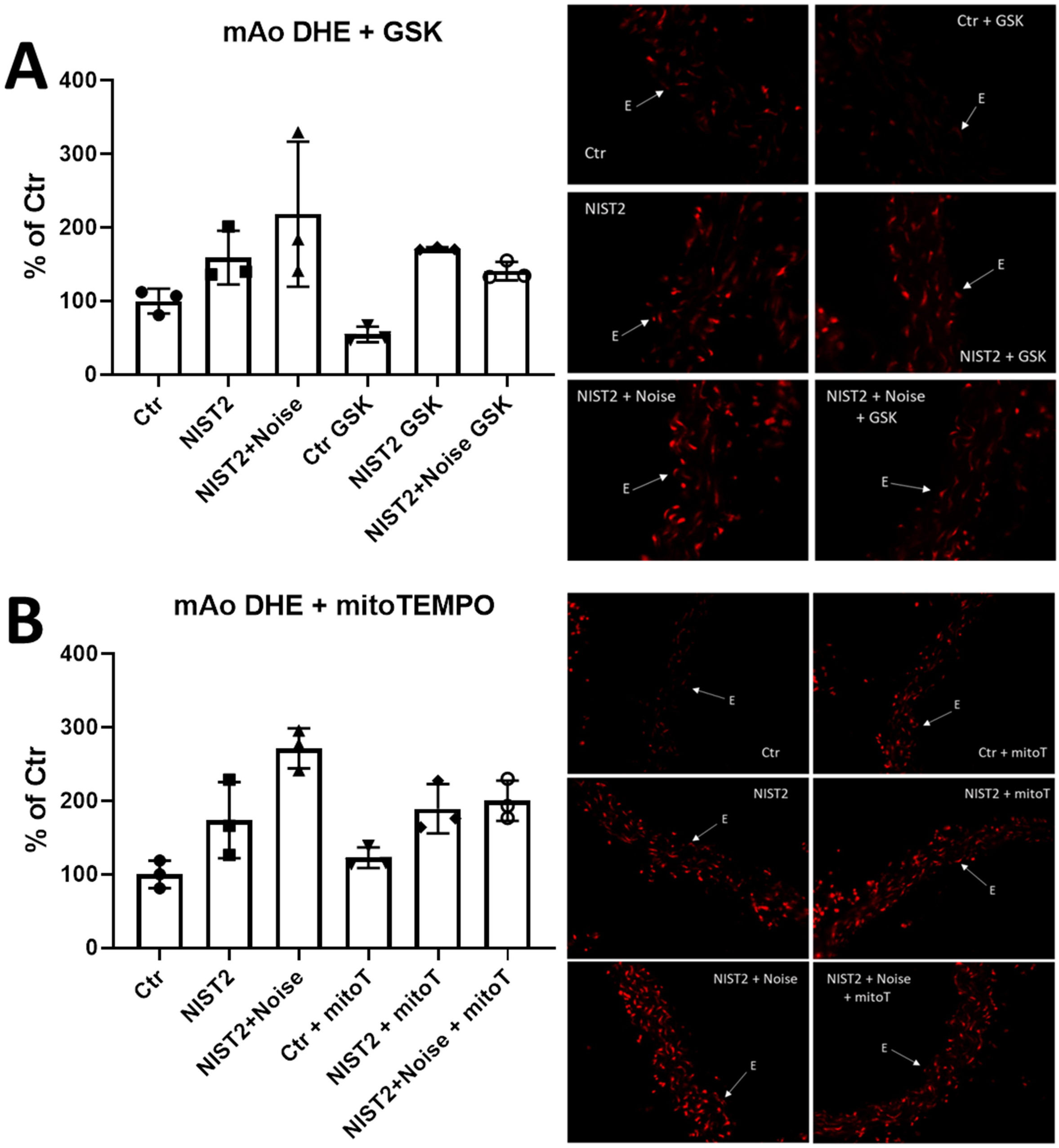

Figure S3. Effects of the combined PM and noise exposure on the vascular oxidative stress. Vascular ROS was assessed by dihydroethidium (DHE, 1 μM) fluorescence microtopography in aortic cryo-sections. (A) Effect of the NOX inhibitor GSK2795039. (B) Effect of the mitochondrial ROS scavenger mitoTEMPO. Representative images show the red fluorescence originating from oxidized DHE products. “E” indicates the endothelium.

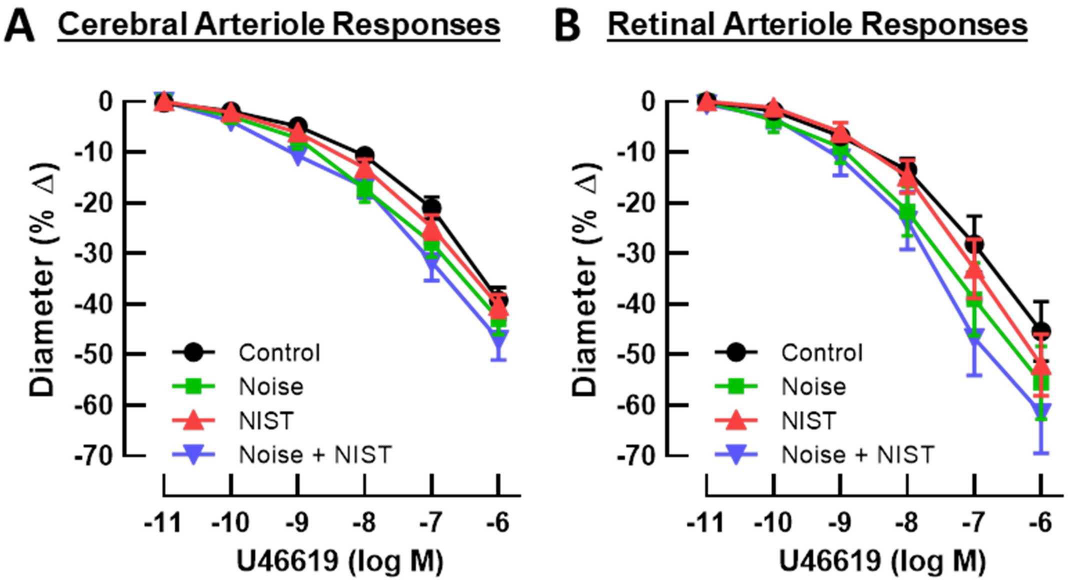

Figure S4. Effects of the combined PM and noise exposure on the vascular function of cerebral and retinal microvessels. Vascular function was assessed in cerebral arterioles (A) and in retinal arterioles (B). The arteriole vasoconstriction was assessed in the presence of U46619 (a synthetic analogue of prostaglandin H_2 and activator of the thromboxane A2 receptor). The endothelium-independent vasoreactivity to U46619 was not affected in any exposure group. The statistics was performed from n = 8 (A) and 6 (B).

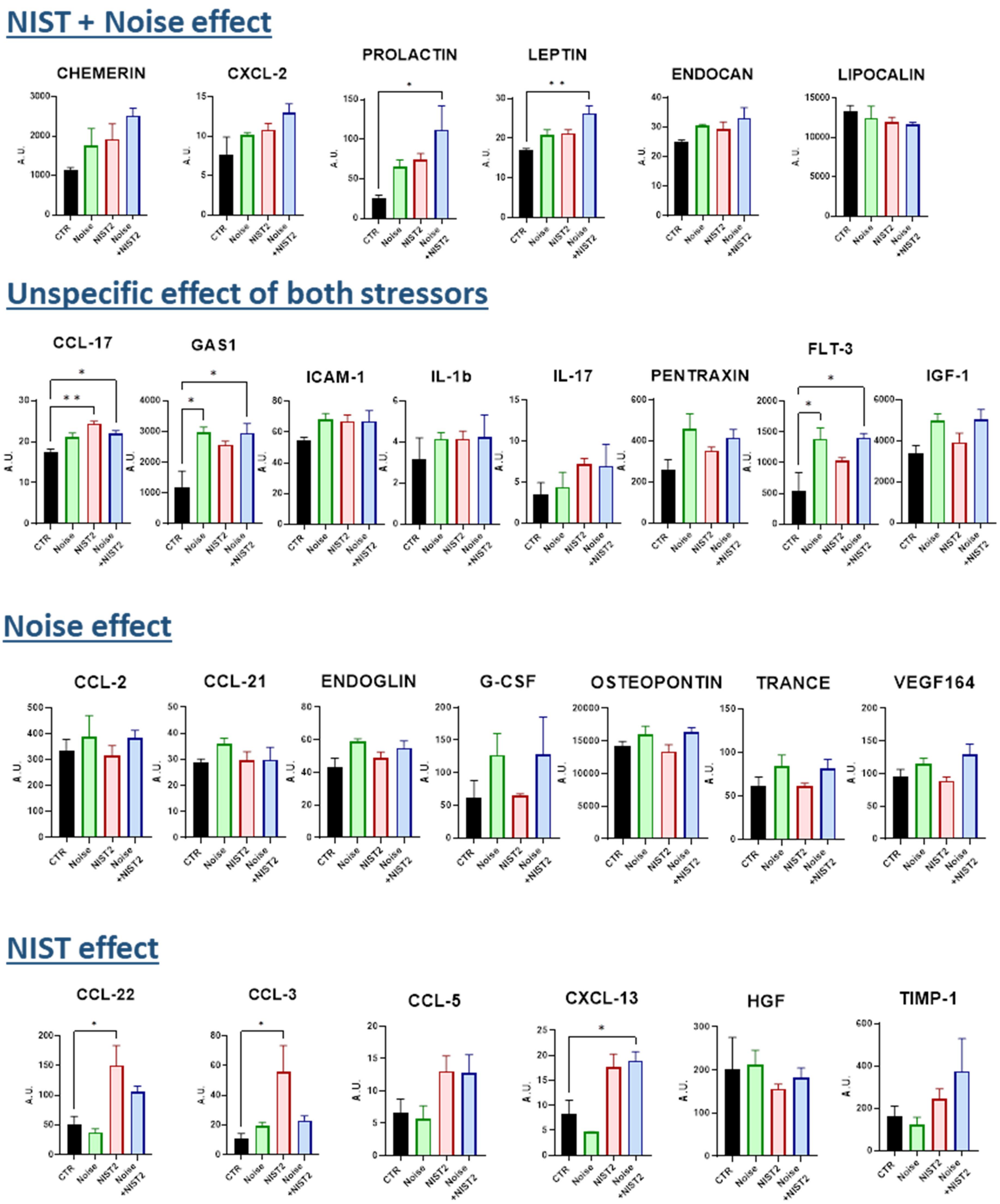

Figure S5. Detailed effects of noise, NIST2 and combined noise+NIST2 on the expression levels of various cytokines and chemokines. Discrimination between additive effects of both stressors and pure noise or NIST2 effects. Data are mean ± SEM, n = 3.

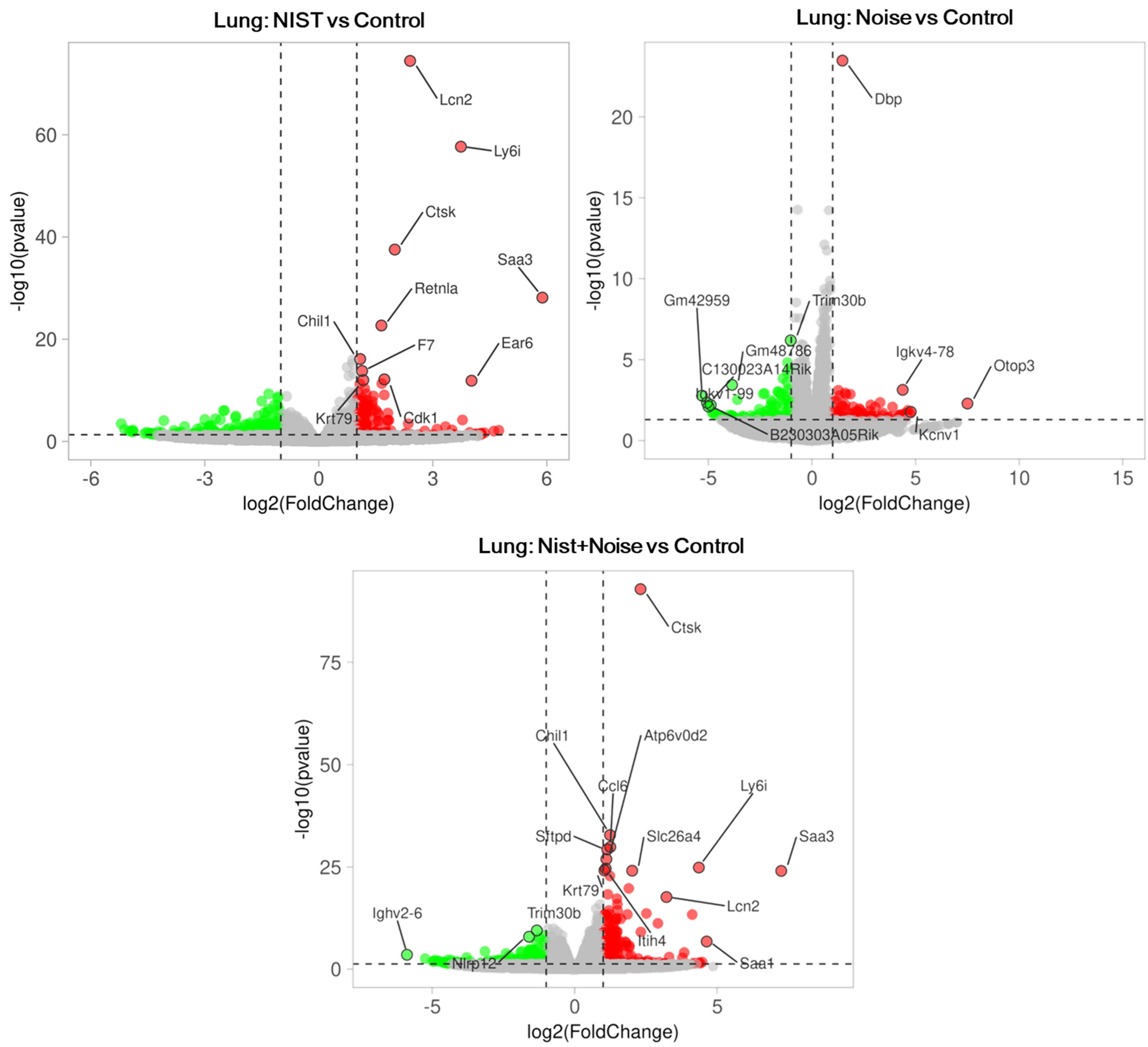

Figure S6. Volcano plots of transcriptomic data in the lung. Effects of PM (NIST2)+noise, PM and noise alone are shown. The log2(FoldChange) indicates the mean expression level for each gene. Each dot represents one gene; The green dots denote down-regulated gene expression, the red dots denote up-regulated gene expression, and the grey dots denote gene expression that are not significant. [log2(FoldChange) threshold = ±1, significance p-value threshold = 0.5].

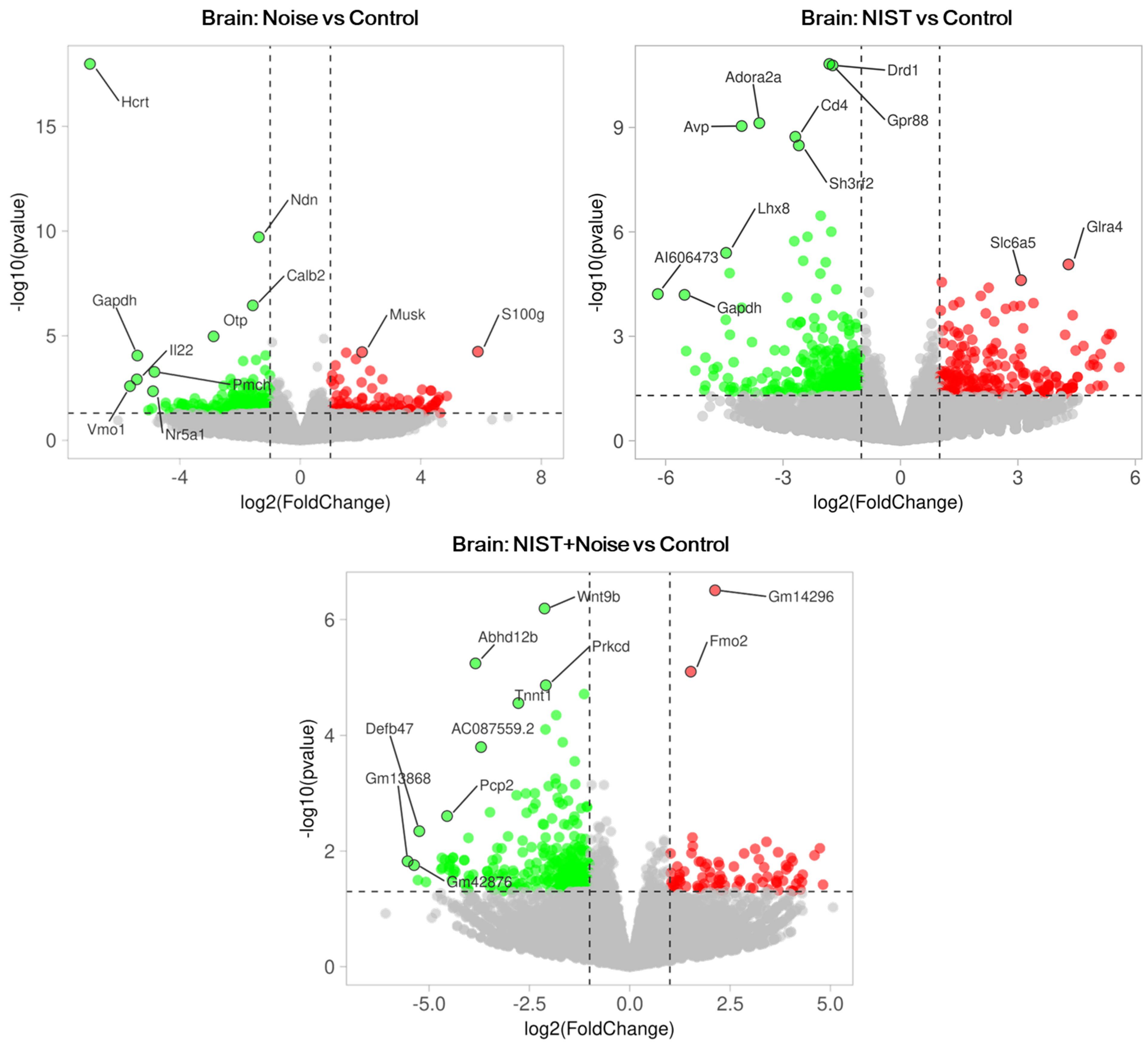

Figure S7. Volcano plots of transcriptomic data in the brain. Effects of PM (NIST2)+noise, PM and noise alone are shown. The log2(FoldChange) indicates the mean expression level for each gene. Each dot represents one gene; The green dots denote down-regulated gene expression, the red dots denote up-regulated gene expression, and the grey dots denote gene expression that are not significant. [log2(FoldChange) threshold = ±1, significance p-value threshold = 0.5].

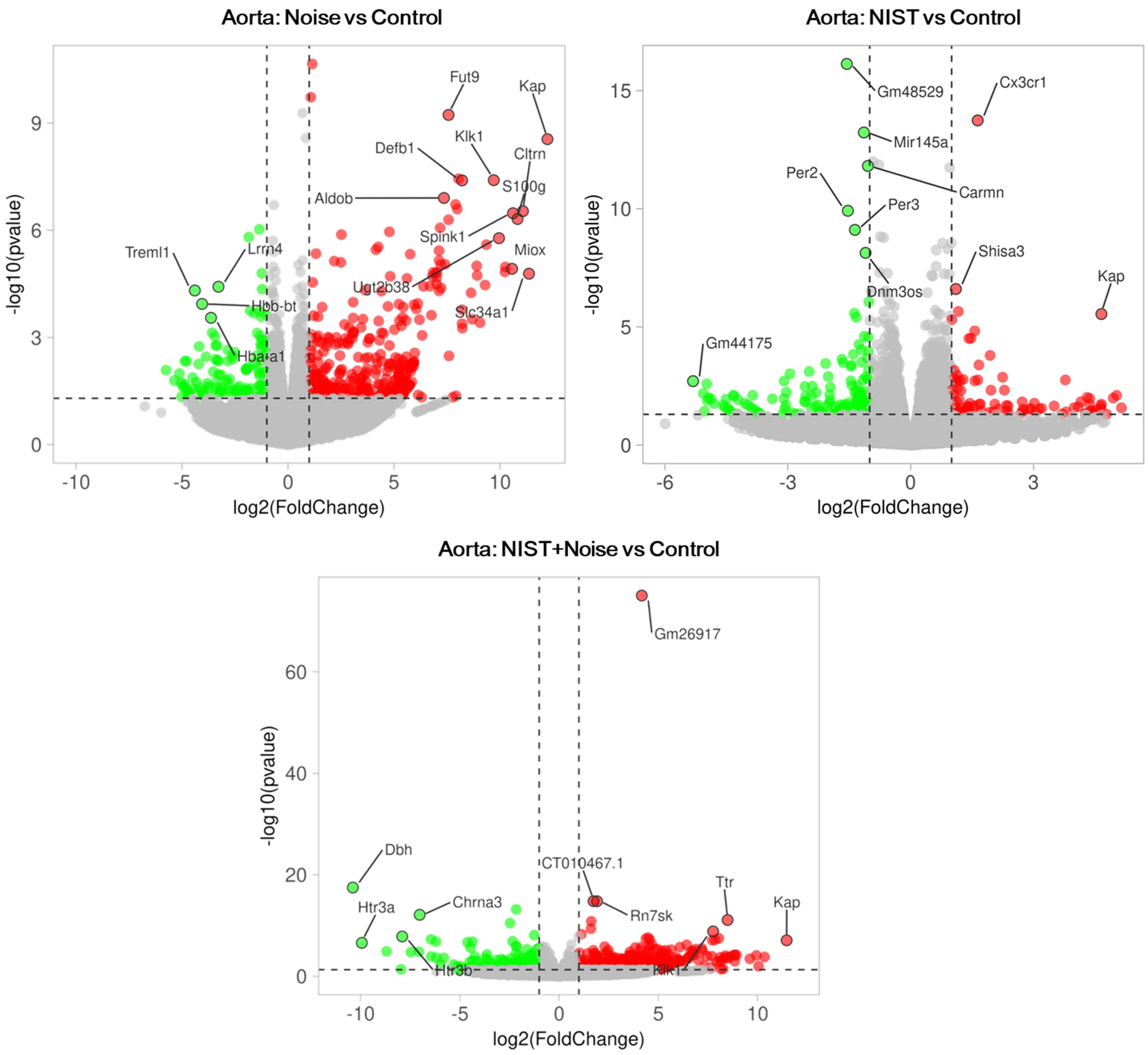

Figure S8. Volcano plots of transcriptomic data in the aorta. Effects of PM (NIST2)+noise, PM and noise alone are shown. The log2(FoldChange) indicates the mean expression level for each gene. Each dot represents one gene; The green dots denote down-regulated gene expression, the red dots denote up-regulated gene expression, and the grey dots denote gene expression that are not significant. [log2(FoldChange) threshold = ±1, significance p-value threshold = 0.5].

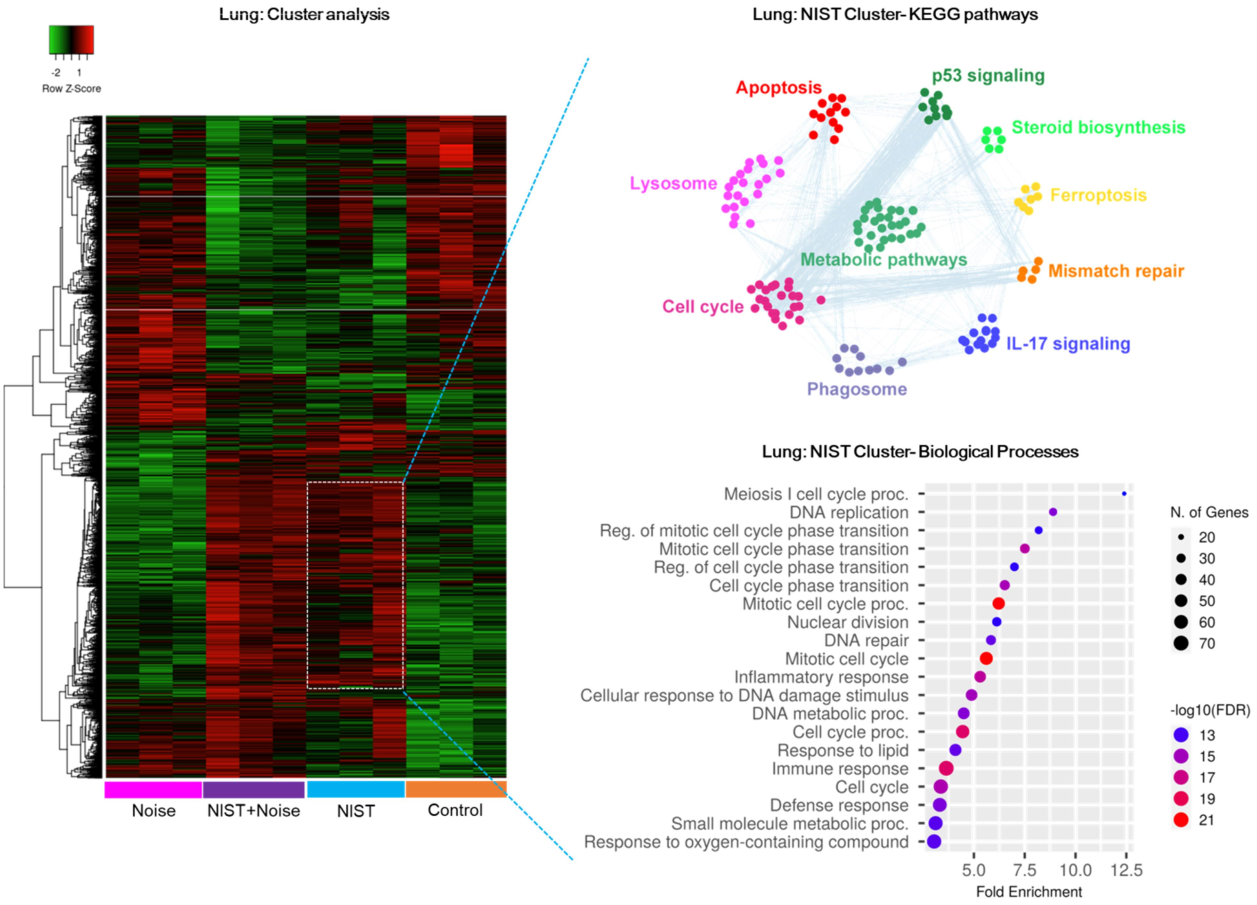

Figure S9. Heat map of cluster analysis in the lung with detailed KEGG pathways and biological processes of selected clusters. Effects of PM (NIST2) alone are shown. Heatmap colours indicate scaled data for the defined conditions (branching of the cluster dendrogram). The intensities were z-scored and are displayed as colours ranging from green to red as shown in the key (n=3). The most enriched KEGG (Kyoto Encyclopedia of Genes and Genomes) pathways identified by functional enrichment analysis from an increased cluster with the effect of NIST in lung tissue (B), n=3. The protein-protein interaction (PPI) network was constructed by the STRING Protein−Protein Interaction Network and visualized via Cytoscape software. KEGG pathway enrichment analysis was performed using the Cytoscape Bingo plugin and the protein-protein interaction network (PPI) network was grouped into relevant protein clusters using the Cytoscape MCODE plugin. $P < 0.05$ was considered statistically significant for KEGG analysis. Each node (coloured dots) represents one gene, the edge (lines) indicates the interaction relationship. Significantly enriched biological processes were identified for the cluster using Biological Networks Gene Ontology with Benjamini and Hochberg multiple testing correction (P<0.05).

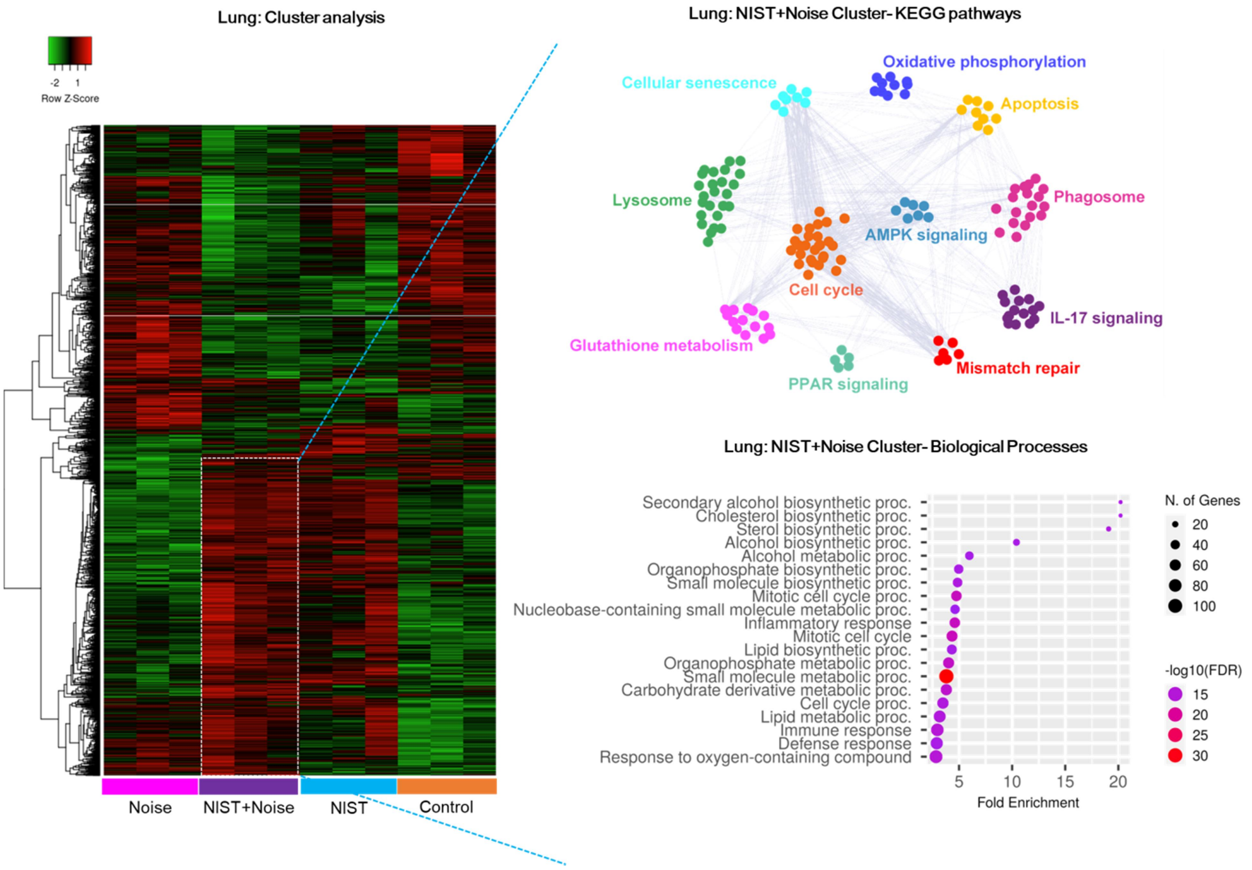

Figure S10. Heat map of cluster analysis in the lung with detailed KEGG pathways and biological processes of selected clusters. Effects of noise+PM (NIST2) are shown. Heatmap colours indicate scaled data for the defined conditions (branching of the cluster dendrogram). The intensities were z-scored and are displayed as colours ranging from green to red as shown in the key (n=3). The most enriched KEGG (Kyoto Encyclopedia of Genes and Genomes) pathways identified by functional enrichment analysis from an increased cluster with the combined effect of NIST+Noise in lung tissue (B), n=3. The protein-protein interaction (PPI) network was constructed by the STRING Protein−Protein Interaction Network and visualized via Cytoscape software. KEGG pathway enrichment analysis was performed using the Cytoscape Bingo plugin and the protein-protein interaction network (PPI) network was grouped into relevant protein clusters using the Cytoscape MCODE plugin. $P < 0.05$ was considered statistically significant for KEGG analysis. Each node (coloured dots) represents one gene, the edge (lines) indicates the interaction relationship. Significantly enriched biological processes were identified for the cluster using Biological Networks Gene Ontology with Benjamini and Hochberg multiple testing correction (P<0.05).

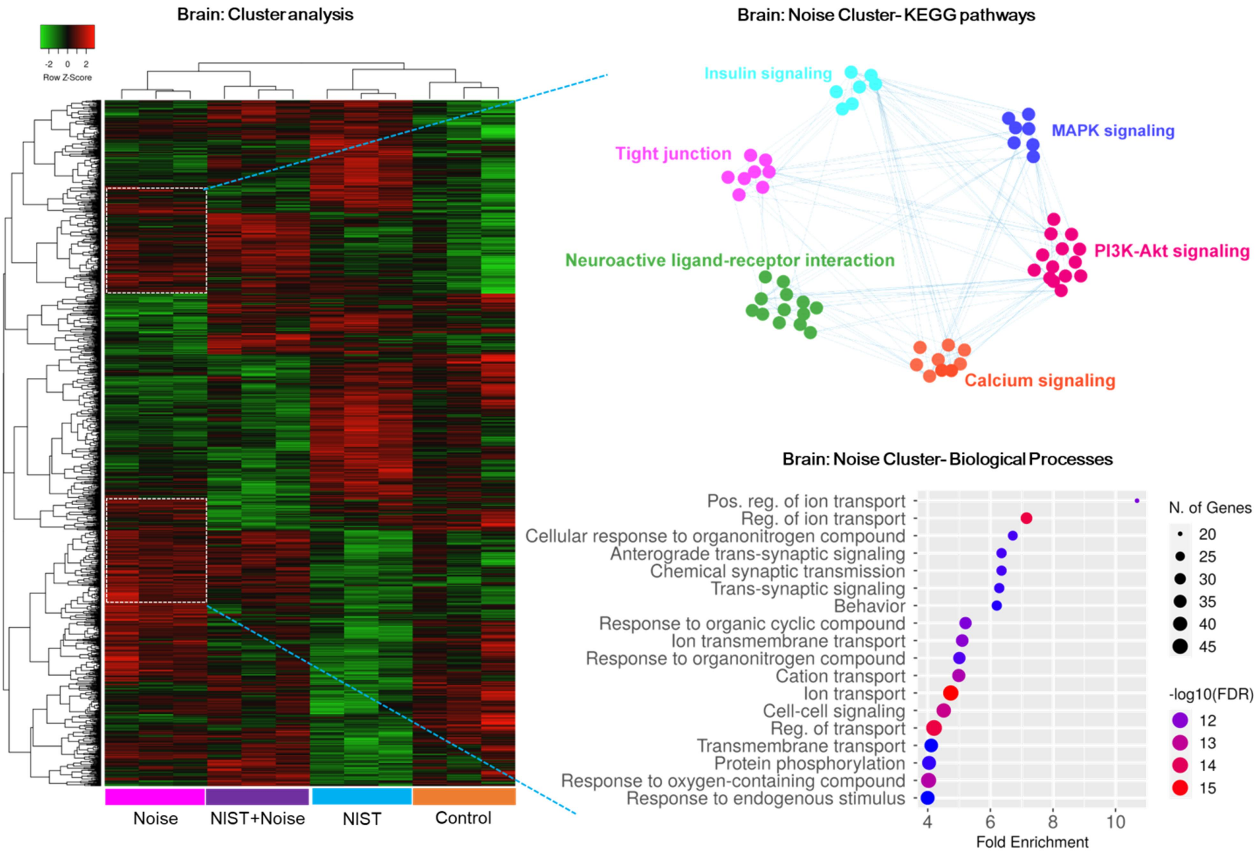

Figure S11. Heat map of cluster analysis in the brain with detailed KEGG pathways and biological processes of selected clusters. Effects of noise alone are shown. Heatmap colours indicate scaled data for the defined conditions (branching of the cluster dendrogram). The intensities were z-scored and are displayed as colours ranging from green to red as shown in the key (n=3). The most enriched KEGG (Kyoto Encyclopedia of Genes and Genomes) pathways identified by functional enrichment analysis from an increased cluster with the effect of Noise in brain tissue (B), n=3. The protein-protein interaction (PPI) network was constructed by the STRING Protein−Protein Interaction Network and visualized via Cytoscape software. KEGG pathway enrichment analysis was performed using the Cytoscape Bingo plugin and the protein-protein interaction network (PPI) network was grouped into relevant protein clusters using the Cytoscape MCODE plugin. $P < 0.05$ was considered statistically significant for KEGG analysis. Each node (coloured dots) represents one gene, the edge (lines) indicates the interaction relationship. Significantly enriched biological processes were identified for the cluster using Biological Networks Gene Ontology with Benjamini and Hochberg multiple testing correction ($P<0.05$).

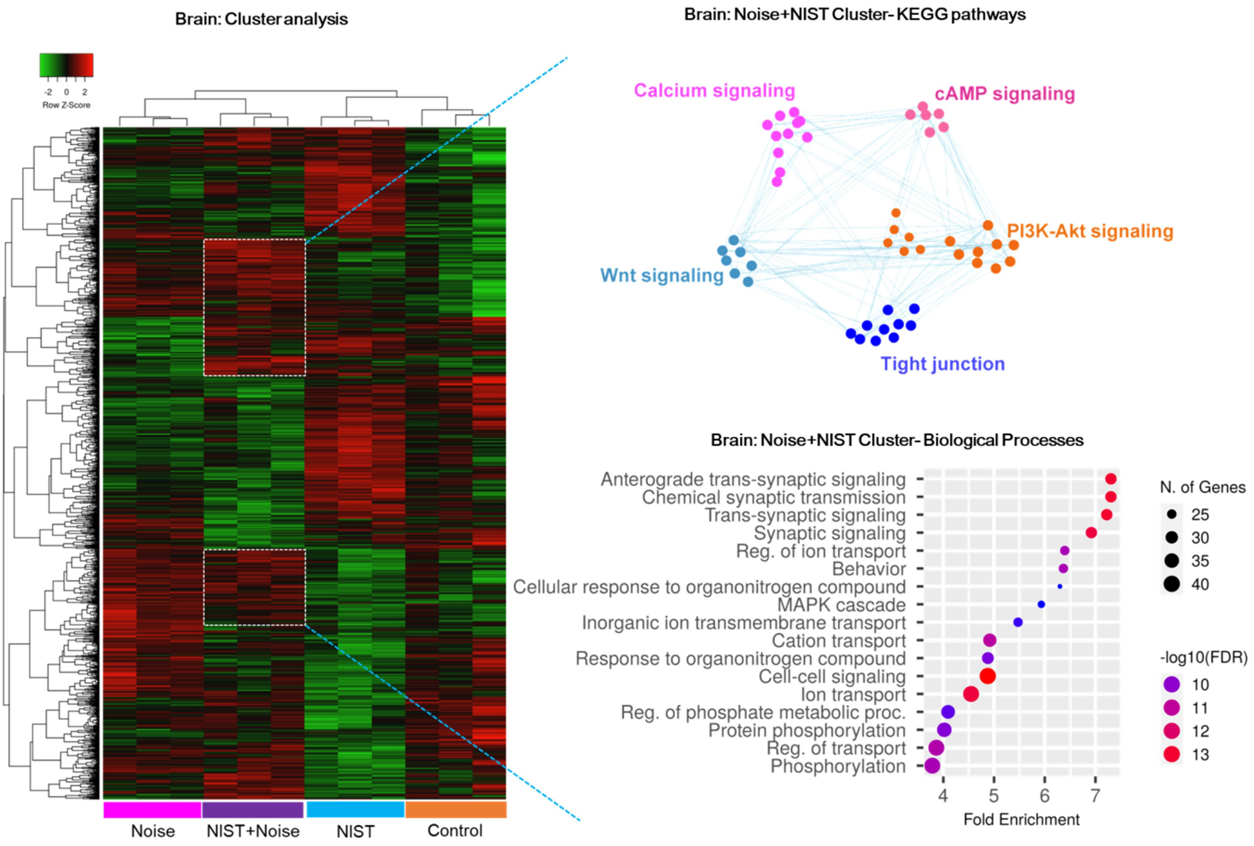

Figure S12. Heat map of cluster analysis in the brain with detailed KEGG pathways and biological processes of selected clusters. Effects of noise+PM (NIST2) are shown. Heatmap colours indicate scaled data for the defined conditions (branching of the cluster dendrogram). The intensities were z-scored and are displayed as colours ranging from green to red as shown in the key (n=3). The most enriched KEGG (Kyoto Encyclopedia of Genes and Genomes) pathways identified by functional enrichment analysis from an increased cluster with the combined effect of Noise+NIST in brain tissue (B), n=3. The protein-protein interaction (PPI) network was constructed by the STRING Protein−Protein Interaction Network and visualized via Cytoscape software. KEGG pathway enrichment analysis was performed using the Cytoscape Bingo plugin and the protein-protein interaction network (PPI) network was grouped into relevant protein clusters using the Cytoscape MCODE plugin. $P < 0.05$ was considered statistically significant for KEGG analysis. Each node (coloured dots) represents one gene, the edge (lines) indicates the interaction relationship. Significantly enriched biological processes were identified for the cluster using Biological Networks Gene Ontology with Benjamini and Hochberg multiple testing correction (P<0.05).

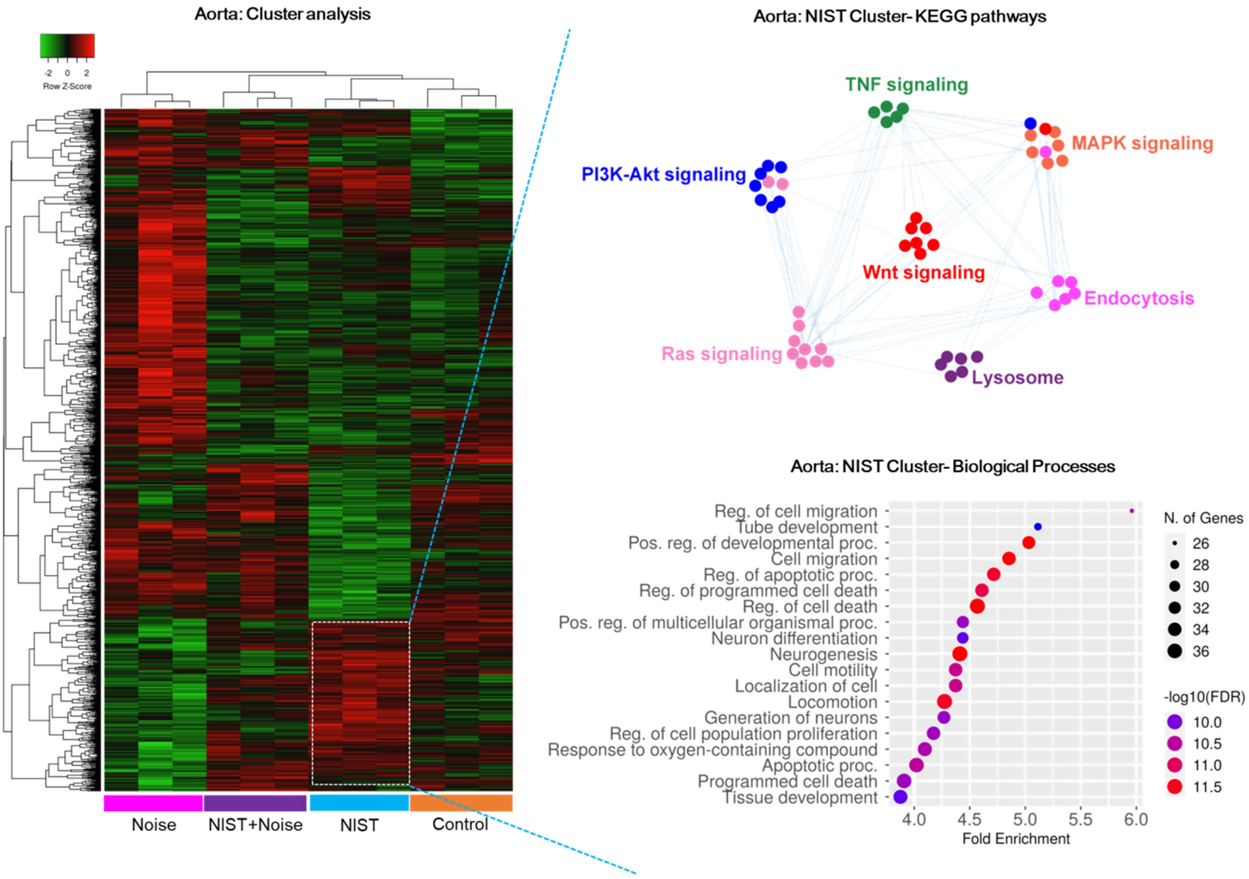

Figure S13. Heat map of cluster analysis in the aorta with detailed KEGG pathways and biological processes of selected clusters. Effects of PM (NIST2) alone are shown. Heatmap colours indicate scaled data for the defined conditions (branching of the cluster dendrogram). The intensities were z-scored and are displayed as colours ranging from green to red as shown in the key (n=3). The most enriched KEGG (Kyoto Encyclopedia of Genes and Genomes) pathways identified by functional enrichment analysis from an increased cluster with the effect of NIST in aorta tissue (B), n=3. The protein-protein interaction (PPI) network was constructed by the STRING Protein−Protein Interaction Network and visualized via Cytoscape software. KEGG pathway enrichment analysis was performed using the Cytoscape Bingo plugin and the protein-protein interaction network (PPI) network was grouped into relevant protein clusters using the Cytoscape MCODE plugin. $P < 0.05$ was considered statistically significant for KEGG analysis. Each node (coloured dots) represents one gene, the edge (lines) indicates the interaction relationship. Significantly enriched biological processes were identified for the cluster using Biological Networks Gene Ontology with Benjamini and Hochberg multiple testing correction (P<0.05).

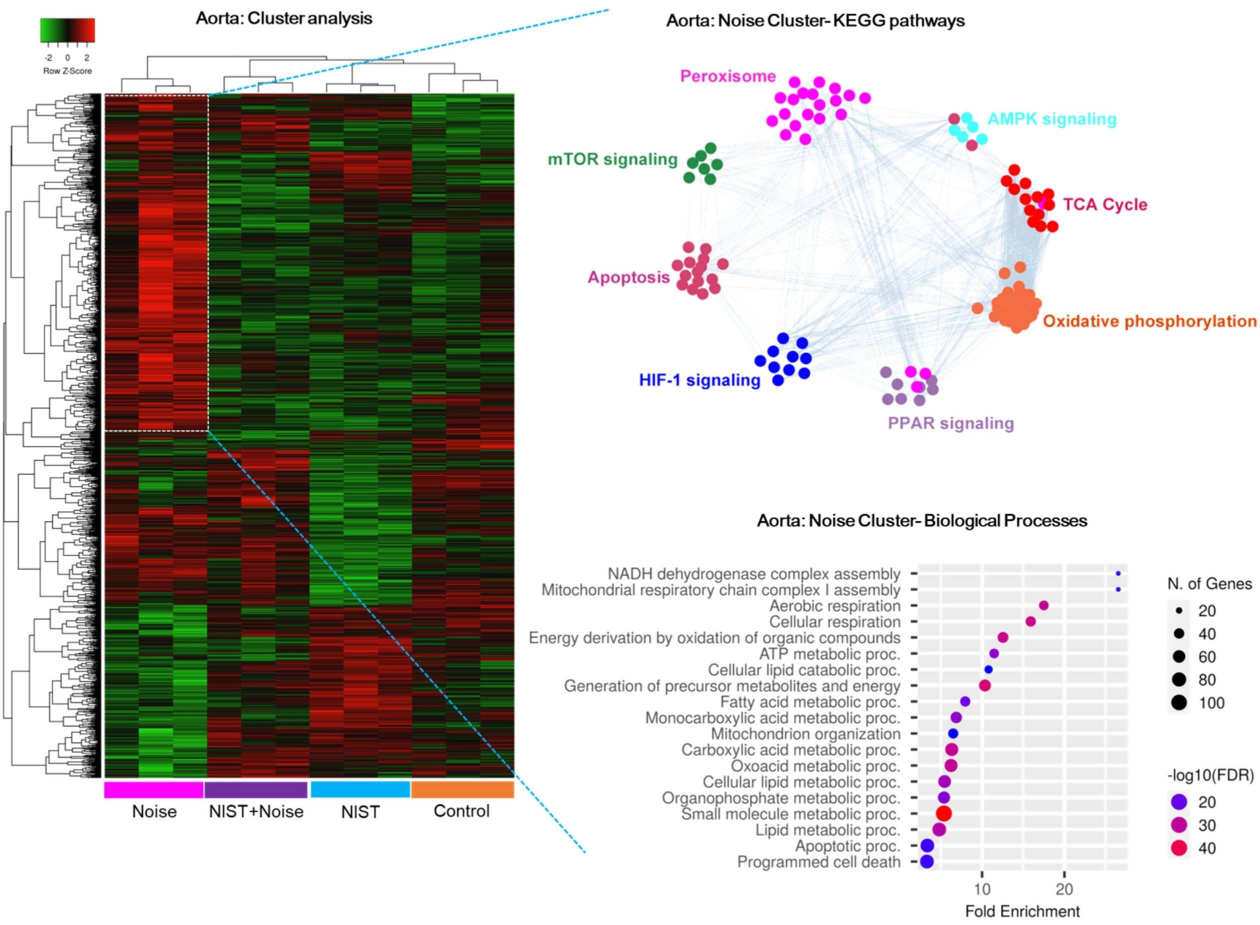

Figure S14. Heat map of cluster analysis in the aorta with detailed KEGG pathways and biological processes of selected clusters. Effects of noise alone are shown. Heatmap colours indicate scaled data for the defined conditions (branching of the cluster dendrogram). The intensities were z-scored and are displayed as colours ranging from green to red as shown in the key (n=3). The most enriched KEGG (Kyoto Encyclopedia of Genes and Genomes) pathways identified by functional enrichment analysis from an increased cluster with the effect of Noise in aorta tissue (B), n=3. The protein-protein interaction (PPI) network was constructed by the STRING Protein−Protein Interaction Network and visualized via Cytoscape software. KEGG pathway enrichment analysis was performed using the Cytoscape Bingo plugin and the protein-protein interaction network (PPI) network was grouped into relevant protein clusters using the Cytoscape MCODE plugin. $P < 0.05$ was considered statistically significant for KEGG analysis. Each node (coloured dots) represents one gene, the edge (lines) indicates the interaction relationship. Significantly enriched biological processes were identified for the cluster using Biological Networks Gene Ontology with Benjamini and Hochberg multiple testing correction (P<0.05).

Extended cited literature

[1] D.L. Costa, K.L. Dreher. Bioavailable transition metals in particulate matter mediate cardiopulmonary injury in healthy and compromised animal models. Environ Health Perspect 105 Suppl 5 (1997) 1053-1060, https://doi.org/10.1289/ehp.97105s51053.

[2] S. Lelieveld, J. Wilson, E. Dovrou, A. Mishra, P.S.J. Lakey, M. Shiraiwa, U. Poschl, T. Berkemeier. Hydroxyl Radical Production by Air Pollutants in Epithelial Lining Fluid Governed by Interconversion and Scavenging of Reactive Oxygen Species. Environ Sci Technol 55 (2021) 14069-14079, https://doi.org/10.1021/acs.est.1c03875.

[3] J.G. Charrier, C. Anastasio. Rates of Hydroxyl Radical Production from Transition Metals and Quinones in a Surrogate Lung Fluid. Environ Sci Technol 49 (2015) 9317-9325, https://doi.org/10.1021/acs.est.5b01606.

[4] S. Offer, E. Hartner, S. Di Bucchianico, C. Bisig, S. Bauer, J. Pantzke, E.J. Zimmermann, X. Cao, S. Binder, E. Kuhn, A. Huber, S. Jeong, U. Kafer, P. Martens, A. Mesceriakovas, J. Bendl, R. Brejcha, A. Buchholz, D. Gat, T. Hohaus, N. Rastak, G. Jakobi, M. Kalberer, T. Kanashova, Y. Hu, C. Ogris, A. Marsico, F. Theis, M. Pardo, T. Groger, S. Oeder, J. Orasche, A. Paul, T. Ziehm, Z.H. Zhang, T. Adam, O. Sippula, M. Sklorz, J. Schnelle-Kreis, H. Czech, A. Kiendler-Scharr, Y. Rudich, R. Zimmermann. Effect of Atmospheric Aging on Soot Particle Toxicity in Lung Cell Models at the Air-Liquid Interface: Differential Toxicological Impacts of Biogenic and Anthropogenic Secondary Organic Aerosols (SOAs). Environ Health Perspect 130 (2022) 27003, https://doi.org/10.1289/EHP9413.

[5] M. Michal Krzyzanowski, J.S. Apte, S.P. Bonjour, M. Brauer, A.J. Cohen, A.M. Pruss-Ustun. Air Pollution in the Mega-cities. Global Environmental Health and Sustainability 1 (2014) 185–191, https://doi.org/10.1007/s40572-014-0019-7.

[6] B. Lv, J. Cai, B. Xu, Y. Bai. Understanding the Rising Phase of the PM(2.5) Concentration Evolution in Large China Cities. Sci Rep 7 (2017) 46456, https://doi.org/10.1038/srep46456.

[7] A. Haghani, R. Johnson, N. Safi, H. Zhang, M. Thorwald, A. Mousavi, N.C. Woodward, F. Shirmohammadi, V. Coussa, J.P. Wise, Jr., H.J. Forman, C. Sioutas, H. Allayee, T.E. Morgan, C.E. Finch. Toxicity of urban air pollution particulate matter in developing and adult mouse brain: Comparison of total and filter-eluted nanoparticles. Environ Int 136 (2020) 105510, https://doi.org/10.1016/j.envint.2020.105510.

[8] D. Li, R. Zhang, L. Cui, C. Chu, H. Zhang, H. Sun, J. Luo, L. Zhou, L. Chen, J. Cui, S. Chen, B. Mai, S. Chen, J. Yu, Z. Cai, J. Zhang, Y. Jiang, M. Aschner, R. Chen, Y. Zheng, W. Chen. Multiple organ injury in male C57BL/6J mice exposed to ambient particulate matter in a real-ambient PM exposure system in Shijiazhuang, China. Environ Pollut 248 (2019) 874-887, https://doi.org/10.1016/j.envpol.2019.02.097.

[9] C.G. Tankersley, R.S. Fitzgerald, R.C. Levitt, W.A. Mitzner, S.L. Ewart, S.R. Kleeberger. Genetic control of differential baseline breathing pattern. J Appl Physiol (1985) 82 (1997) 874-881, https://doi.org/10.1152/jappl.1997.82.3.874.

[10] F.P. Schmidt, M. Basner, G. Kroger, S. Weck, B. Schnorbus, A. Muttray, M. Sariyar, H. Binder, T. Gori, A. Warnholtz, T. Munzel. Effect of nighttime aircraft noise exposure on endothelial function and stress hormone release in healthy adults. Eur Heart J 34 (2013) 3508-3514a, https://doi.org/10.1093/eurheartj/eht269.

[11] F. Schmidt, K. Kolle, K. Kreuder, B. Schnorbus, P. Wild, M. Hechtner, H. Binder, T. Gori, T. Munzel. Nighttime aircraft noise impairs endothelial function and increases blood pressure in patients with or at high risk for coronary artery disease. Clin Res Cardiol 104 (2015) 23-30, https://doi.org/10.1007/s00392-014-0751-x.

[12] F.P. Schmidt, J. Herzog, B. Schnorbus, M.A. Ostad, L. Lasetzki, O. Hahad, G. Schafers, T. Gori, M. Sorensen, A. Daiber, T. Munzel. The impact of aircraft noise on vascular

and cardiac function in relation to noise event number: a randomized trial. Cardiovasc Res 117 (2021) 1382-1390, https://doi.org/10.1093/cvr/cvaa204.
[13] T. Munzel, A. Daiber, S. Steven, L.P. Tran, E. Ullmann, S. Kossmann, F.P. Schmidt, M. Oelze, N. Xia, H. Li, A. Pinto, P. Wild, K. Pies, E.R. Schmidt, S. Rapp, S. Kroller-Schon. Effects of noise on vascular function, oxidative stress, and inflammation: mechanistic insight from studies in mice. Eur Heart J 38 (2017) 2838-2849, https://doi.org/10.1093/eurheartj/ehx081.
[14] S. Kroller-Schon, A. Daiber, S. Steven, M. Oelze, K. Frenis, S. Kalinovic, A. Heimann, F.P. Schmidt, A. Pinto, M. Kvandova, K. Vujacic-Mirski, K. Filippou, M. Dudek, M. Bosmann, M. Klein, T. Bopp, O. Hahad, P.S. Wild, K. Frauenknecht, A. Methner, E.R. Schmidt, S. Rapp, H. Mollnau, T. Munzel. Crucial role for Nox2 and sleep deprivation in aircraft noise-induced vascular and cerebral oxidative stress, inflammation, and gene regulation. Eur Heart J 39 (2018) 3528-3539, https://doi.org/10.1093/eurheartj/ehy333.
[15] S. Steven, K. Frenis, S. Kalinovic, M. Kvandova, M. Oelze, J. Helmstadter, O. Hahad, K. Filippou, K. Kus, C. Trevisan, K.D. Schluter, K. Boengler, S. Chlopicki, K. Frauenknecht, R. Schulz, M. Sorensen, A. Daiber, S. Kroller-Schon, T. Munzel. Exacerbation of adverse cardiovascular effects of aircraft noise in an animal model of arterial hypertension. Redox Biol 34 (2020) 101515, https://doi.org/10.1016/j.redox.2020.101515.
[16] J. Eckrich, K. Frenis, G. Rodriguez-Blanco, Y. Ruan, S. Jiang, M.T. Bayo Jimenez, M. Kuntic, M. Oelze, O. Hahad, H. Li, A. Gericke, S. Steven, S. Strieth, A. von Kriegsheim, T. Munzel, B.P. Ernst, A. Daiber. Aircraft noise exposure drives the activation of white blood cells and induces microvascular dysfunction in mice. Redox Biol 46 (2021) 102063, https://doi.org/10.1016/j.redox.2021.102063.
[17] K. Frenis, J. Helmstadter, Y. Ruan, E. Schramm, S. Kalinovic, S. Kroller-Schon, M.T. Bayo Jimenez, O. Hahad, M. Oelze, S. Jiang, P. Wenzel, C.J. Sommer, K.B.M. Frauenknecht, A. Waisman, A. Gericke, A. Daiber, T. Munzel, S. Steven. Ablation of lysozyme M-positive cells prevents aircraft noise-induced vascular damage without improving cerebral side effects. Basic Res Cardiol 116 (2021) 31, https://doi.org/10.1007/s00395-021-00869-5.
[18] M. Feng, S. Whitesall, Y. Zhang, M. Beibel, L. D'Alecy, K. DiPetrillo. Validation of volume-pressure recording tail-cuff blood pressure measurements. Am J Hypertens 21 (2008) 1288-1291, https://doi.org/10.1038/ajh.2008.301.
[19] T. Munzel, A. Giaid, S. Kurz, D.J. Stewart, D.G. Harrison. Evidence for a role of endothelin 1 and protein kinase C in nitroglycerin tolerance. Proc Natl Acad Sci U S A 92 (1995) 5244-5248, https://doi.org/10.1073/pnas.92.11.5244.
[20] J. Eckrich, Y. Ruan, S. Jiang, K. Frenis, G. Rodriguez-Blanco, A.P. Maas, M.T.B. Jimenez, M. Kuntic, M. Oelze, O. Hahad, H. Li, S. Steven, S. Strieth, A. von Kriegsheim, T. Munzel, A. Daiber, A. Gericke, B.P. Ernst. In vivo analysis of noise dependent activation of white blood cells and microvascular dysfunction in mice. MethodsX 8 (2021) 101540, https://doi.org/10.1016/j.mex.2021.101540.
[21] A. Gericke, E. Goloborodko, N. Pfeiffer, C. Manicam. Preparation Steps for Measurement of Reactivity in Mouse Retinal Arterioles Ex Vivo. J Vis Exp (2018), https://doi.org/10.3791/56199.
[22] A. Gericke, J.J. Sniatecki, E. Goloborodko, A. Steege, O. Zavaritskaya, J.M. Vetter, F.H. Grus, A. Patzak, J. Wess, N. Pfeiffer. Identification of the muscarinic acetylcholine receptor subtype mediating cholinergic vasodilation in murine retinal arterioles. Invest Ophthalmol Vis Sci 52 (2011) 7479-7484, https://doi.org/10.1167/iovs.11-7370.
[23] P. Wenzel, E. Schulz, M. Oelze, J. Muller, S. Schuhmacher, M.S. Alhamdani, J. Debrezion, M. Hortmann, K. Reifenberg, I. Fleming, T. Munzel, A. Daiber. AT1-receptor blockade by telmisartan upregulates GTP-cyclohydrolase I and protects eNOS

in diabetic rats. Free Radic Biol Med 45 (2008) 619-626, https://doi.org/10.1016/j.freeradbiomed.2008.05.009.

[24] A. Mortazavi, B.A. Williams, K. McCue, L. Schaeffer, B. Wold. Mapping and quantifying mammalian transcriptomes by RNA-Seq. Nat Methods 5 (2008) 621-628, https://doi.org/10.1038/nmeth.1226.

[25] J. Goedhart, M.S. Luijsterburg. VolcaNoseR is a web app for creating, exploring, labeling and sharing volcano plots. Sci Rep 10 (2020) 20560, https://doi.org/10.1038/s41598-020-76603-3.

[26] S. Tyanova, T. Temu, P. Sinitcyn, A. Carlson, M.Y. Hein, T. Geiger, M. Mann, J. Cox. The Perseus computational platform for comprehensive analysis of (prote)omics data. Nat Methods 13 (2016) 731-740, https://doi.org/10.1038/nmeth.3901.
